# FGF19 promotes cell autophagy and cisplatin chemoresistance by activating MAPK signaling in ovarian cancer

**DOI:** 10.7717/peerj.14827

**Published:** 2023-02-02

**Authors:** Wei Zhu, Meiyuan Huang, Abhimanyu Thakur, Yuanliang Yan, Xiaoying Wu

**Affiliations:** 1 Department of Pathology, Xiangya Hospital, Central South University, Changsha, China; 2 Department of Pathology, School of Basic Medical Science, Central South University, Changsha, China; 3 Department of Pathology, Zhuzhou Hospital Affiliated to Xiangya School of Medicine, Central South University, Zhuzhou, China; 4 Pritzker School of Molecular Engineering, Ben May Department for Cancer Research, University of Chicago, Chicago, Illinois, USA; 5 Department of Pharmacy, Xiangya Hospital, Central South University, Changsha, China; 6 National Clinical Research Center for Geriatric Disorders, Xiangya Hospital, Central South University, Changsha, China

**Keywords:** Ovarian cancer, Autophagy, FGF19, MAPK, Chemoresistance

## Abstract

**Background:**

Chemotherapy is one of the primary treatments for ovarian cancer patients. Autophagy has been linked to chemotherapy resistance in tumor cells. Recent studies have suggested that fibroblast growth factor 19 (FGF19) may be involved in the onset and progression of malignancies. However, the relationship between FGF19 and autophagy in ovarian cancer is still unknown.

**Methods:**

Next-generation sequencing (NGS) was conducted to analyze gene mutation profiles of 62 cases of high grade serous ovarian cancer (HGSOC). Fluorescence *in situ* hybridization (FISH) was performed to validate the amplification of FGF19 in HGSOC tissues. Quantitative PCR (qPCR) and immunohistochemistry (IHC) were used to analyze the difference of FGF19 in mRNA and protein expression. Meanwhile, bioinformatics techniques were used to analyze the expression profiles of FGF19 and the correlation with prognosis. Besides, immunofluorescence, transmission electron microscopy and Cell Counting Kit 8 (CCK-8) were used to investigate the potential mechanisms.

**Results:**

In this study, we found that FGF19 promotes cisplatin resistance in ovarian cancer cells by inducing autophagy. NGS analysis of 62 HGSOC cases identified a significantly amplified gene, FGF19. In addition, the expression level of FGF19 in ovarian cancer samples was higher than that in normal samples. FISH results showed a positive correlation between amplification and expression of FGF19. Knockdown of FGF19 inhibited the cell autophagy through decrease in the expression of LC3 and Beclin 1, and increase in the expression of SQSTM1/p62. Furthermore, we observed that p38 MAPK phosphorylation was down-regulated after FGF19 knockdown. IFN-γ, a potential p38 MAPK activator, counteracted the inhibition of cell autophagy and the anti-proliferation effect of cisplatin induced by FGF19 knockdown in ovarian cancer cells.

**Conclusion:**

FGF19 increases autophagy and chemoresistance in ovarian cancer by activating the p38 MAPK pathway. These results could point to FGF19 being a potential therapeutic target for ovarian cancer.

## Introduction

High grade serous ovarian cancers (HGSOC) are the most common type of ovarian cancer and are responsible for the majority of ovarian cancer deaths. This is largely due to the fact that they are often diagnosed at a later stage, resulting in a poor prognosis (Andrikopoulou et al., 2021; Suh-Burgmann et al., 2021). Even after clinical treatment, many HGSOC patients are still prone to relapse and chemotherapy resistance (Chandrasekaran & Elias, 2021; Matthews, Bowden & Wong-Brown, 2021). Therefore, it is important to investigate the underlying mechanisms of chemoresistance in order to improve the survival and prognosis of HGSOC patients.

Autophagy is a process by which cellular material is delivered to lysosomes or vacuoles for degradation and recycling (Chen et al., 2021b). Cell autophagy has important regulatory roles on the cancer pathogenesis and therapeutic response (Liu et al., 2021b). For instance, *Fusobacterium nucleatum* has been found to confer chemoresistance in oesophageal squamous cell carcinoma cells by inducing autophagy (Liu et al., 2021a). In gastric cancer, Zinc oxide nanoparticle (ZnO-NP) alleviates the chemoresistance by inhibiting autophagy (Miao et al., 2021). Abnormally expressed SH3BGRL could drive the chemotherapy resistance through enhancing cell autophagy in breast cancer (Zhang et al., 2022b).

The fibroblast growth factor family (FGF) is involved in multiple biological processes, including embryogenesis, angiogenesis, tissue homeostasis, and cancer progression (Presta et al., 2017). FGF19, one of the hormone-like FGFs, is frequently overexpressed and amplified in many cancers. Upon endoplasmic reticulum stress, amplified FGF19 promotes tumor cell proliferation through activating fibroblast growth factor (FGF) receptor 4 (FGFR4)-glycogen synthase kinase-3beta (GSK3β)-nuclear factor erythroid-2-related factor-2 (Nrf2) signaling in hepatocellular carcinoma (Teng et al., 2017). Moreover, the upregulated FGF19 can promote ovarian cancer proliferation and invasion by activating mitogen-activated protein kinase (MAPK) signaling pathway (Hu & Cong, 2015). However, the association between FGF19 and autophagy in ovarian cancer has not been investigated.

In this study, we investigated the roles of FGF19 in the regulation of autophagy and chemotherapy response in ovarian cancer. We found that FGF19 was amplified and overexpressed in ovarian cancer cells. FGF19 knockdown inhibited autophagy and decreased cisplatin resistance in ovarian cancer cells by decreasing phosphorylation of p38 MAPK.

## Materials and Methods

### Patients

A cohort of 157 HGSOC specimens, 26 normal ovaries and 22 normal fallopian tubes was obtained from Xiangya Hospital of Central South University from September 2016 to September 2021. Among these, 118 HGSOC, 26 paired normal ovaries and 22 normal fallopian tube were used for immunohistochemistry (IHC). A total of 62 diagnosis HGSOC samples were used for next-generation sequencing (NGS). A total of 41 HGSOC samples were used for fluorescence *in situ* hybridization (FISH). Specimens were collected in accordance with the ethical standards of Xiangya Hospital of Central South University. The Ethical Committee of Xiangya Hospital of Central South University approved this study (Approval No. 202201021).

### Cells culture

Human ovarian cancer cells, OVCAR3, HO8910, HO8910pm, SKOV3, SKOV3-IP, A2780 and normal ovarian epithelial cells IOSE were obtained from School of Basic Medical Science, Central South University. All the cells were cultured in Dulbecco’s Modified Eagle Medium (DMEM) medium (Bi, Israel Beit Haemek Ltd., Beit Haemek, Israel) containing 10% fetal bovine serum (FBS) at 37 °C under an atmosphere with 5% CO_2_.

### Chemicals and reagents

BIRB796 and cisplatin were purchased from Good Laboratory Practice Bioscience. IFN-γ was purchased from MedChemExpress. FGF19 and p62 antibodies were obtained from Santa. Antibodies against LC3 and Beclin 1, along with secondary antibodies, were purchased from Cell Signaling Technology. Antibodies against p38 MAPK, phospho-p38 MAPK (p-p38 MAPK) and GAPDH were purchased from Abclonal.

### IHC

After the tissue sections were dewaxed in turpentine oil and hydrated in gradient alcohol (95%, 85%, 75%, 50%), the exposure of tissue antigen was repaired with citrate buffer for 3 min. Endogenous peroxidase activity was blocked by using 3% H_2_O_2_ for 20 min. The sections were incubated with FGF19 antibody at 37 °C for 1h, and incubated with secondary antibody for 30 min. After then, color reaction was analyzed with 3,3′-diaminobenzitine (DAB) solution.

### FISH

According to the manufacturer’s recommendations, FISH was performed on tissue using two probes targeting FGF19-CCND1 and CEP11 (Abnova, Walnut, CA, USA). The slides were examined using a 100× objective lens and an Olympus BX51 fluorescence microscope (Olympus, Tokyo, Japan). Each image is obtained by Olympus cellsens software.

### Western blot

The same amount of celluar protein in each sample was separated by SDS-PAGE, and then imprinted on PVDF membrane (Millipore, Burlington, MA, USA; Merck, Rahway, NJ, USA). After blocking with 5% milk, the membrane was incubated with primary antibodies overnight at 4 °C, and then incubated with HRP-conjugated secondary antibodies at room temperature for 2 h. Then, the Chemiluminescence imaging system was used to detect the immune response.

### RNA extraction and qPCR

The total RNA was extracted by TRIzol reagent (Invitrogen, Waltham, MA, USA), reverse transcribed into cDNA using PrimeScript RT Kit (Abclonal, Wuhan, China) according to the manufacturer’s instructions. qRT-PCR was performed by iTaqTM Universal SYBR green Supermix (Abclonal, Wuhan, China). GAPDH was used as internal control. The forward and reverse primers were used as follows: FGF19: 5′-CGGAGGAAGACTGTGCTTTCG-3′ and 5′-CTCGGATCGGTACACATTGTAG-3′; GAPDH: 5′-CAGCAAGAGCACAAGAGGAA-3′ and 5′-TGGTACATGACAAGGTGCGG-3′. Relative expression levels were decided using the 2^−ΔΔCT^ method.

### Immunofluorescence

HO8910pm and SKOV3-IP cells were seeded into 6-well plates. After transfection and/or adding BIRB796, the cells were fixed with 4% formaldehyde for 20 min at room temperature. After staining with 2-(4-Amidinophenyl)-6-indolecarbamidine dihydrochloride (DAPI) and sealing the slide, the formation of GFP-LC3 puncta in cells were analyzed by confocal microscope.

### CCK-8 assays

Cells (1 × 10^3^ cells/well) were inoculated in 96 well plates after treatment with cisplatin. Then, after adding CCK-8 test solution (b34304, Bimake, Houston, TX, USA), the cells were cultured at 37 °C in 5% CO_2_ for 1 h. Subsequently, the optical density (OD) of the sample was measured at 450 nm using a Microplate Reader (Biotech, Camarillo, CA, USA).

### Statistical analysis

SPSS 22.0 and GraphPad Prism 8.0 were used for all statistical analyses. Data have been expressed as means ± Standard Error of Mean. The two groups were compared by Student’s t-test. The correlations between the FGF19 expression and FGF19 amplification were analyzed by Spearman’s correlation analysis. All experiments were performed at least three times and are reported as mean ± SD. Statistical significance is shown as with *p-value < 0.05, **p-value < 0.01, ***p-value < 0.001 and ****p-value < 0.0001.

## Results

### Identification of FGF19 amplification in ovarian cancer

**Figure 1:**
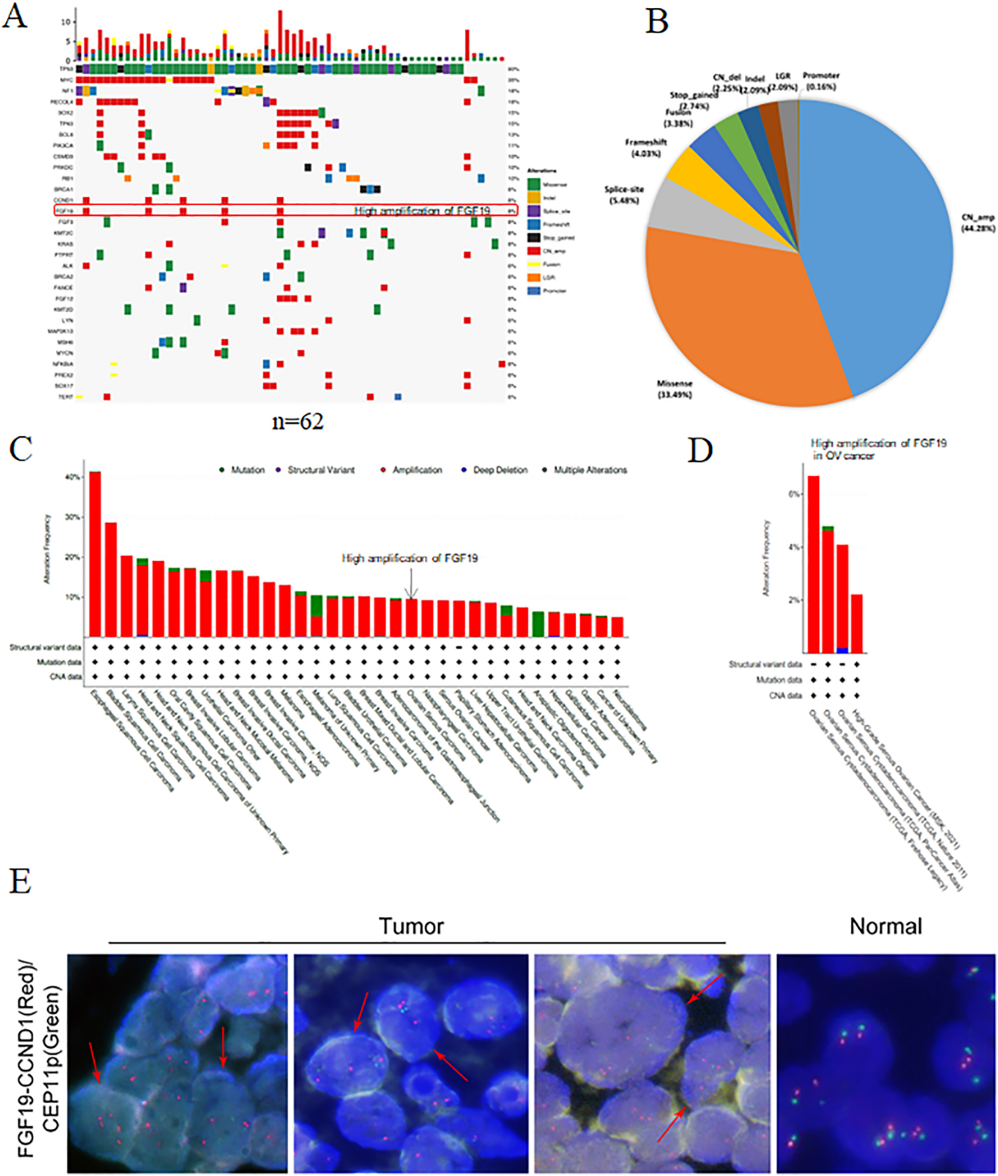
The gene mutation profiles revealed the FGF19 amplification in ovarian cancer. (A) Distribution and frequency of genetic alterations in 62 HGSOC patients. Mutation types are marked with different colors. (B) Pie chart showing the percentage of different types of somatic mutations in HGSOC. (C and D) The mutation frequency of FGF19 analyzed by cBioPortal database in pan-cancer and ovarian cancer. (E) FISH assay was performed to detect FGF19 amplification (red arrows) in OC tissues.

Using NGS technology, we detected and analyzed the gene mutation profiles in HGSOC patients (Table S1). Overall, 621 mutations and 273 mutated genes were detected. Among them, the most frequently mutated genes were TP53 (90%), followed by Myc (35%), neurofibromatosis type 1 (NF1, 18%) and RecQ like helicase 4 (RECQL4, 18%) (Fig. 1A). The mutation types were classified into eight categories: missense mutation, deletion mutation, splicing mutation, frameshift mutation, stop-gained variation, copy number variation, gene fusion, large genomic rearrangement and promoter mutation. Among them, copy number amplification was the most type (44.28%), followed by missense mutation (33.49%), splicing mutation (5.48%) and frameshift mutation (4.03%) (Fig. 1B).

Studies have shown that aberrant FGF19 can be a driver of malignant behavior, contributing to the oncogenesis and progression of human cancers (Kanzaki et al., 2021). In our analysis of 62 paraffin-embedded ovarian cancer tissues, we identified FGF19 amplification (Fig. 1A). Additionally, the cBioPortal database revealed FGF19 amplification in a variety of cancers (Fig. 1C) and four ovarian cancer datasets (Fig. 1D). Furthermore, FISH analysis was conducted to validate the amplified FGF19 in HGSOC tissues (Fig. 1E). This showed that FGF19 is highly amplified in ovarian cancer.

### FGF19 was overexpressed in ovarian cancer and correlated with poor prognosis

**Figure 2:**
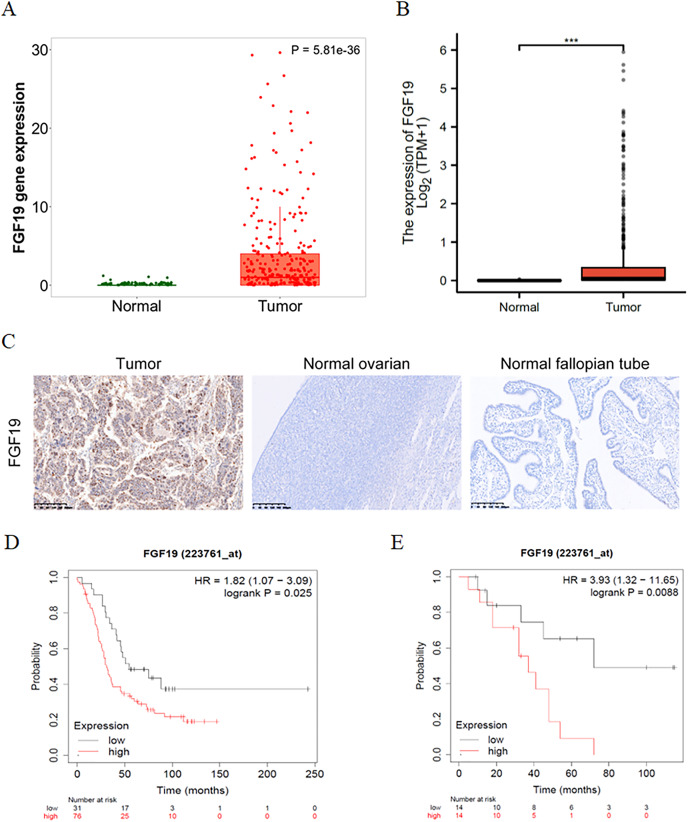
FGF19 was overexpressed in OC tissues and correlated with the poor prognosis. (A and B) The expression of FGF19 was analyzed by the (A) TNM plot and (B) TCGA. (C) IHC staining for FGF19 in OC sample (*n* = 118), normal ovarian (*n* = 26) and normal fallopian tube (*n* = 22). (D and E) The relationship between FGF19 expression and overall survival (OS) analyzed by Kaplan–Meier Plotter in GSE26193 (D) and GSE19829 (E). ***p < 0.001.

The expression profiles of FGF19 were analyzed by several bioinformatics databases. First, the analysis results from TNMplot database showed that the expression of FGF19 mRNA in ovarian cancer tissues was higher than that in normal tissues (Fig. 2A). From TCGA-OC, we found that the expression of FGF19 was significantly upregulated in ovarian cancer tissues (Fig. 2B). Next, we analyzed the association between FGF19 expression and patients’ clinical characteristics. IHC was performed to explore the FGF19 level in 118 ovarian cancer tissues, 22 normal ovarian and 26 normal fallopian tube. FGF19 was expressed in both nucleus and cytoplasm, and the expression of FGF19 in tumor tissues was higher than that in normal tissues (Fig. 2C). Next, we studied the correlation between FGF19 expression and the clinical characteristics, such as age, clinical stage and lymphatic metastasis (Table 1). The expression of FGF19 was related to lymphatic metastasis and abdominal and distant metastasis (*p* = 0.001, *p* = 0.028, respectively), and high expression of FGF19 was more prone to tumor metastasis. The Kaplan Meier plotter database suggested that the patients with high level of FGF19 displayed poor prognosis in GSE26193 (Fig. 2D) and GSE19829 (Fig. 2E). Collectively, the overexpressed FGF19 in ovarian cancer could be utilized as a potential biomarker for prognosis.

**Table 1:** Correlation of FGF19 expression with clinicopathological features.

Characteristics	Nunbers of case (%)	FGF19 expression		*p* value
Age (y)		Low	High	
≥50	79(66.9)	28	11	0.001
<50	39(33.1)	32	47	
Clinical stage				
I	14(11.9)	9	5	0.06
II	9(7.6)	8	1	
III	77(65.3)	35	42	
IV	18(15.3)	8	10	
Lymphatic metastasis				
Yes	49(41.5)	16	33	0.001
No	69(58.5)	44	25	
Abdominal and distant metastasis				
Yes	94(79.7)	43	51	0.028
No	24(20.3)	17	7	
Histological type				
Ovarian cancer	118	60	58	
Normal ovarian	26	25	1	<0.001
Normal fallopian tube	22	17	5	0.011

### FGF19 knockdown inhibited cell autophagy

**Figure 3:**
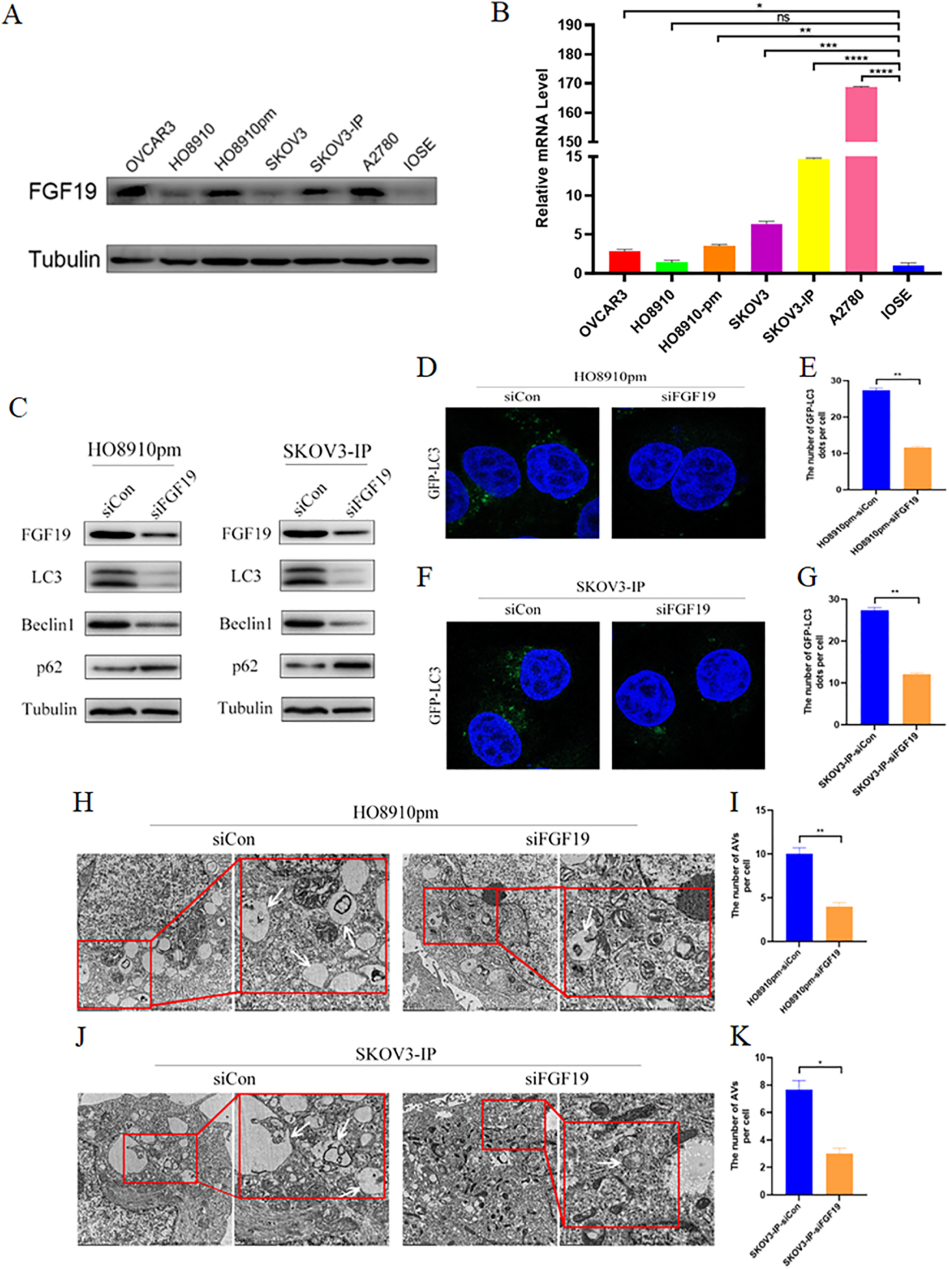
FGF19 modulates autophagy in OC cells. (A and B) Expression of FGF19 was analyzed by western blot and qRT-PCR. (C) Western blot analysis of LC3, Beclin 1, and p62 protein levels after FGF19 knockdown in HO8910pm and SKOV3-IP cells. (D–G) Representative images of GFP-LC3 after FGF19 knockdown in HO8910pm and SKOV3-IP cells. (H–K) Transmission electron microscopy indicated the decreased formation of autophagosomes (white arrows) after FGF19 knockdown in HO8910pm and SKOV3-IP cells. *p < 0.05, **p < 0.01, ***p < 0.001 and ****p < 0.0001, ns, not significant.

We screened the expression profiles of FGF19 in several ovarian cancer cells, and found that FGF19 were over-expressed in multiple ovarian cancer cells, including HO8910pm and SKOV3-IP (Figs. 3A and 3B). Recent study displayed that cell autophagy-associated signaling affects the clinical outcomes and therapeutic responses in ovarian cancer (Zhang et al., 2022a). To investigate the roles of FGF19 on cell autophagy, we knocked down FGF19 in HO8910pm and SKOV3-IP cells, and found that FGF19 knockdown down-regulated the expression of LC3 and Beclin 1 and up-regulated the expression of SQSTM1/p62 (Fig. 3C). Then, immunofluorescence results displayed that FGF19 knockdown reduced the GFP-LC3 puncta formation in HO8910pm and SKOV3-IP cells (Figs. 3D–3G). Furthermore, autophagy-associated biomarkers and GFP-LC3 puncta formation displayed the opposite results after overexpression of FGF19 in HO8910 and SKOV3 (Fig. S1). The transmission electron microscopy showed that downregulation of FGF19 inhibited the formation of autophagosomes (Figs. 3H–3K). The above-mentioned results suggested that abnormally overexpressed FGF19 could improve cell autophagy in ovarian cancer cells.

### FGF19 knockdown inhibited cell autophagy *via* downregulation MAPK signaling

**Figure 4:**
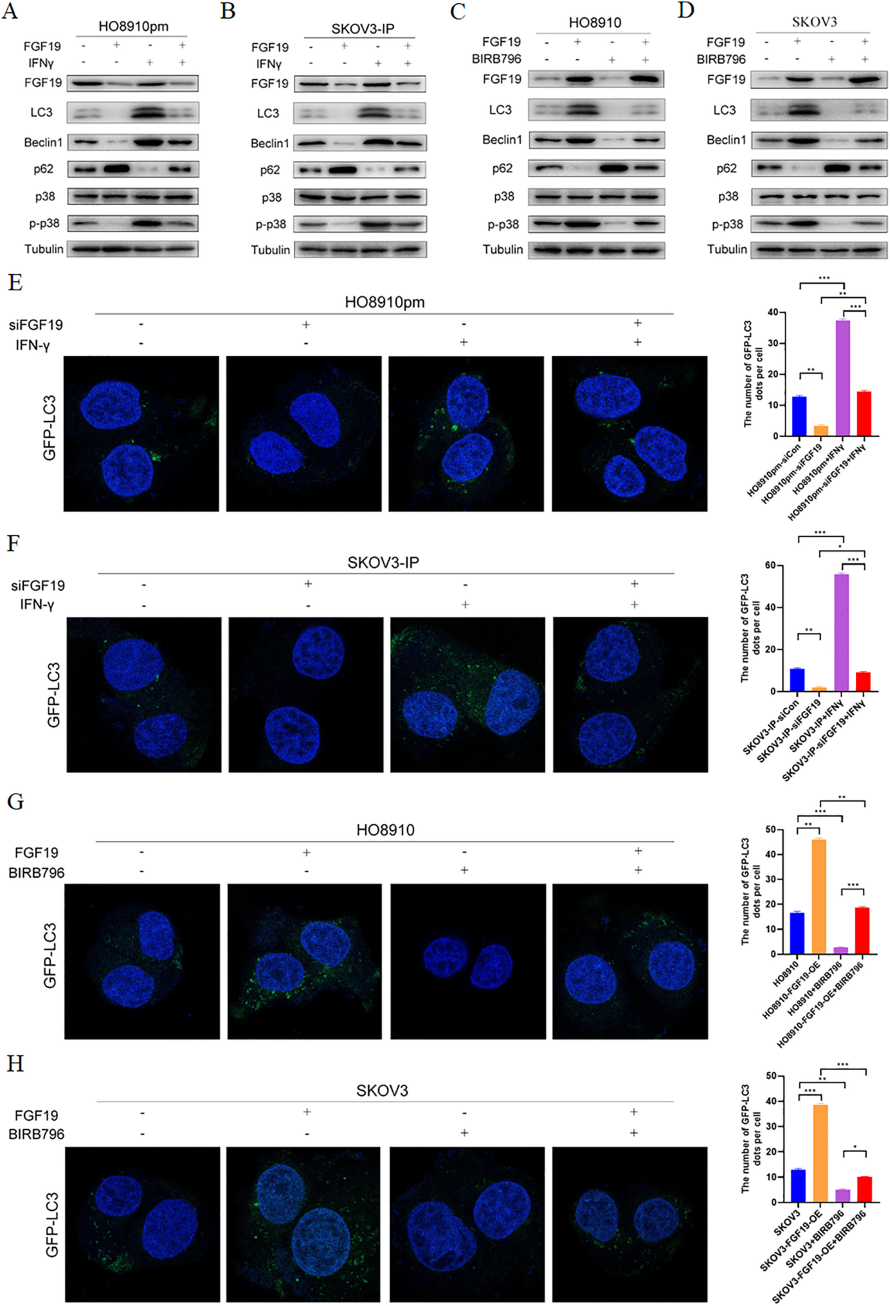
FGF19 promoted cell autophagy by activating p38 MAPK signaling. (A and B) Western blot analysis of FGF19, LC3, Beclin 1, p62, p38 MAPK and p-p38 MAPK after FGF19 knockdown and 200 ng/ml IFN-γ treatment in HO8910pm and SKOV3-IP cells. (C and D) Western blot analysis of FGF19, LC3, Beclin 1, p62, p38 MAPK and p-p38 MAPK after FGF19 overexpression and 400 nmol/l BIRB796 treatment in HO8910 and SKOV3 cells. (E and F) Representative confocal images of GFP-LC3 after FGF19 knockdown and IFN-γ treatment in HO8910pm and SKOV3-IP cells. (G and H) Representative confocal images of GFP-LC3 after FGF19 overexpression and BIRB796 treatment in HO8910 and SKOV3 cells. *p < 0.05, **p < 0.01 and ***p < 0.001.

MAPKs, in particular p38 MAPK, have dual roles in the regulation of cell autophagy (Webber & Tooze, 2010). Some studies have shown that FGF family members, such as FGF2, regulate cell autophagy by inhibiting MAPK signaling (Yuan et al., 2017). Here, we explored whether MAPK signaling participates in the regulatory effect of FGF19 on cell autophagy. Western blot showed the decreased LC3 and Beclin 1 and increased p62 after treatment with FGF19 knockdown, while increased LC3 and Beclin 1 and decreased p62 after treatment with IFN-γ, a potential p38 MAPK activator (Matsuzawa, Fujiwara & Washi, 2014), in HO8910pm and SKOV3-IP cells (Figs. 4A and 4B). Moreover, FGF19 knockdown also inhibited p38 MAPK phosphorylation, whereas IFN-γ did not affect the expression of FGF19, suggesting that p38 MAPK was the downstream target of FGF19. In contrast, FGF19 overexpression up-regulated the expression of LC3 and Beclin 1 and down-regulated the expression of p62, while treated with p38 MAPK inhibitor BIRB796 had the opposite effects in HO8910 and SKOV3 cells (Figs. 4C and 4D). We then transfected GFP-LC3 into ovarian cancers cells, and found that FGF19 downregulation and BIRB796 treatment decreased the formation of GFP-LC3 puncta, while FGF19 upregulation and IFN-γ treatment increased the formation of GFP-LC3 puncta (Figs. 4E–4H). Taken together, these findings suggested that FGF19 knockdown inhibited cell autophagy through deactivating p38 MAPK signaling pathway.

### FGF19 enhanced cisplatin resistance by activating p38 MAPK signaling

**Figure 5:**
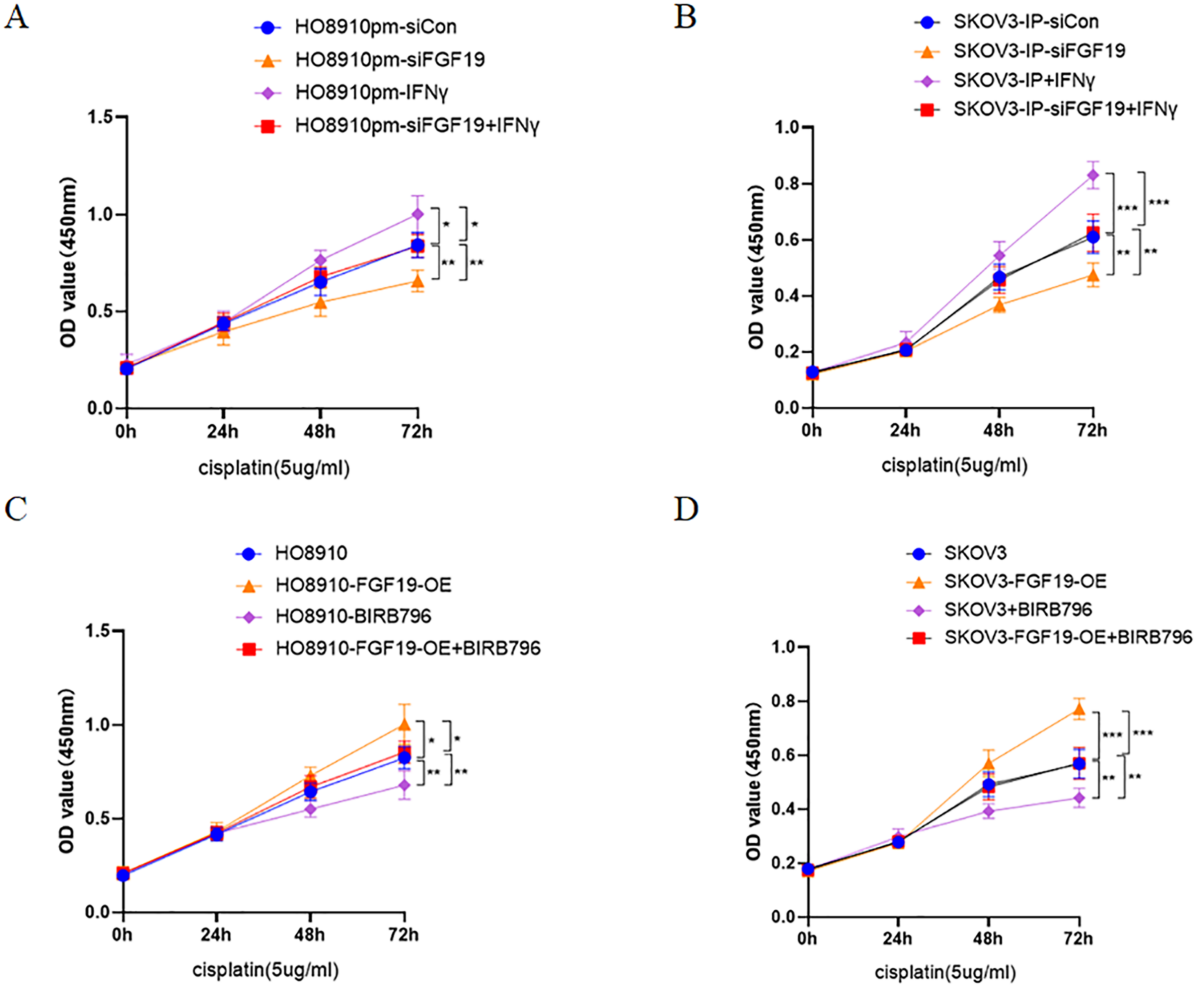
FGF19 promoted cisplatin resistance through activating p38 MAPK signaling. (A and B) After FGF19 knockdown and IFN-γ treatment, the anti-proliferation effect of cisplatin on HO8910pm (A) and SKOV3-IP (B) cells was detected by CCK-8 assay. (C and D) After FGF19 overexpression and BIRB796 treatment, the anti-proliferation effect of cisplatin on HO8910 (C) and SKOV3 (D) cells was detected by CCK8 assay. *p < 0.05, **p <0.01 and ***p < 0.001.

Multiple studies have demonstrated that activation of p38 MAPK is related to cisplatin resistance in a variety of cancers, including ovarian cancer (Chen et al., 2018; Li et al., 2021; Liao et al., 2020). Here, CCK8 was used to detect the potential roles of FGF19-p38 MAPK axis on the anti-proliferation effect of cisplatin in ovarian cancer cells. The results displayed that both FGF19 knockdown and BIRB796 treatment increased cisplatin-induced proliferation inhibition of OC cells; however, FGF19 overexpression and IFN-γ treatment reduced cisplatin-induced proliferation inhibition (Figs. 5A–5D). These results demonstrated that FGF19 might promote the cisplatin resistance of ovarian cancer cells by activating p38 MAPK signaling.

## Discussion

In this study, the roles of FGF19 in cell autophagy and therapeutic response of ovarian cancer cells were demonstrated. High level amplification and expression of FGF19 were observed in ovarian cancer. FGF19 might promote autophagy and cisplatin resistance of ovarian cancer cells by activating p38 MAPK pathway. In addition, patients with highly expressed FGF19 had shorter overall survival (OS). Thus, the results confirmed that FGF19 might be a prognostic and therapeutic target of ovarian cancer.

FGFs is a family composed of 22 different proteins, which is divided into seven different subfamilies, including FGF19 subfamily (Itoh & Ornitz, 2004; Presta et al., 2017). Studies have demonstrated that FGF19 is frequently amplified in human cancers (Peng et al., 2020). Moreover, over-expressed FGF19 could accelerate cancer progression and therapeutic resistance. FGF19 can promote the occurrence of colorectal cancer by regulating metabolic biological functions, such as bile acid biosynthesis and insulin resistance (Peng et al., 2020). The expression of FGF19 is significantly up-regulated in non-small cell lung cancer, and is associated with poor prognosis (Chen et al., 2021a). FGF19 stimulates cell proliferation and invasion by activating AKT-MAPK signaling pathway (Hu & Cong, 2015). Therefore, our findings are consistent with the current view that FGF19 might act as an oncogenic gene.

Autophagy is a lysosomal degradation pathway, which is important for survival, differentiation, development and cellular homeostasis. Autophagy plays major roles in various pathologies, including infection, neurodegeneration, heart disease and cancer (Levine & Kroemer, 2008). Autophagy is often induced as a “first response” to cancer therapy. Through autophagy, cells try to clear the damage caused by cancer treatment (Chaachouay et al., 2011). IL-6 activates autophagy through the upregulating JAK2 pathway and promotes chemotherapy resistance in colorectal cancer (Hu et al., 2021). Furthermore, as promising regulators of autophagy, the non-coding RNAs, such as lncRNAs and miRNAs, can regulate therapeutic resistance in cancer cells (Eng et al., 2021; Hou, Li & Dong, 2021; Xia et al., 2022). Moreover, increasing studies have confirmed that autophagy is involved in the progression and drug resistance of ovarian cancer cells (Zhao et al., 2021). In addition, FGF family members display important effects in the regulation of cell autophagy. FGF2 may suppress autophagy by activating the PI3K/Akt pathway, exhibiting a neuroprotective role (Wang et al., 2021). FGF21 exhibits protective effects against cardiomyocyte hypoxia/reoxygenation injury by promoting autophagy (Ren et al., 2019). However, the detailed roles of FGF19 in cell autophagy and chemoresistance in ovarian cancer have not been not fully clarified. Accordingly, our study confirmed that high levels of FGF19 could promote cell autophagy and cisplatin resistance in ovarian cancer cells.

MAPK signaling is one of the common pathways participating in different cytological functions, such as cell proliferation, inflammation, differentiation and apoptosis. MAPK pathway has three-level signal transmission processes: MAPK, MAPK kinase (MEK or MKK) and the kinase of MAPK kinase (MEKK or MKKK). These three kinases could be activated in turn, jointly regulating a variety of important physiological and pathological functions. Bruceine D induces apoptosis and autophagy of lung cancer cells through activating ROS/MAPK signaling pathway *in vivo* and *in vitro* (Fan et al., 2020). PNO1 regulates apoptosis and autophagy of hepatocellular carcinoma through MAPK signaling pathway, facilitating the cancer progression (Han et al., 2021). In recent years, emerging studies have demonstrated that MAPK pathway is related to cancer therapy. Magnoflorine induces apoptosis and autophagy by inhibiting AKT/mTOR and promoting p38 MAPK signaling pathways, and enhances the sensitivity of breast cancer cells to doxorubicin (DOX) (Wei, Xiaojun & Peilong, 2020). HIF-1α And HDAC4 may actively control cisplatin resistance of ovarian cancer through modulating p53/RAS-dependent autophagy (Zhang et al., 2019). Similarly, in present study, FGF19 enhances autophagy and cisplatin resistance in OC by activating MAPK pathway, which may be a useful therapeutic target.

In conclusion, our study illustrated that the underlying mechanisms of FGF19 in the regulation of cell autophagy and chemoresistance in ovarian cancer cells. FGF19 is highly amplified and overexpressed in ovarian cancer, and is associated with patients’ poor prognosis. Moreover, FGF19 enhances cell autophagy and cisplatin chemoresistance through activating p38 MAPK pathway.

## Supplemental Information

10.7717/peerj.14827/supp-1 Supplemental Information 1 The raw data of Western blot.

10.7717/peerj.14827/supp-2 Supplemental Information 2 The raw data of immunofluorescence.

10.7717/peerj.14827/supp-3 Supplemental Information 3 The raw data of immunohistochemical.

10.7717/peerj.14827/supp-4 Supplemental Information 4 The raw data of transmission electron microscopy in Figure 3H.

10.7717/peerj.14827/supp-5 Supplemental Information 5 The raw data of transmission electron microscopy in Figure 3J.

10.7717/peerj.14827/supp-6 Supplemental Information 6 The raw data of fluorescence *in situ* hybridization (FISH).

10.7717/peerj.14827/supp-7 Supplemental Information 7 The raw data of HO8910pm-CCK8 in Figure 5A.

10.7717/peerj.14827/supp-8 Supplemental Information 8 The raw data of statistical analysis in Table 1.

10.7717/peerj.14827/supp-9 Supplemental Information 9 The output file of statistical analysis in Table 1.

10.7717/peerj.14827/supp-10 Supplemental Information 10 Gene mutation profiles in HGSOC patients.

10.7717/peerj.14827/supp-11 Supplemental Information 11 The raw data for mRNA level in Figure 3B.

10.7717/peerj.14827/supp-12 Supplemental Information 12 The raw data of SKOV3-IP-CCK8 in Figure 5B.

10.7717/peerj.14827/supp-13 Supplemental Information 13 The raw data of FGF19 expression in Figure 3B.

10.7717/peerj.14827/supp-14 Supplemental Information 14 The data of bioinformation.

10.7717/peerj.14827/supp-15 Supplemental Information 15 FGF19 modulates autophagy in OC cells. (A-B) Western blot analysis of LC3, Beclin 1, and p62 protein levels after FGF19 overexpression in HO8910 and SKOV3 cells. (C-D) Representative images of GFP-LC3 after F FGF19 overexpression in HO8910 and SKOV3 cells. *p < 0.05, **p < 0.01, ***p < 0.001 and ****p < 0.0001, ns, not significant.

10.7717/peerj.14827/supp-16 Supplemental Information 16 The clinical data of IHC in SPSS.

10.7717/peerj.14827/supp-17 Supplemental Information 17 The codebook of numbers.

10.7717/peerj.14827/supp-18 Supplemental Information 18 Raw data for Figure 2B.

## Additional Information and Declarations

### DNA Deposition

The following information was supplied regarding the deposition of DNA sequences: The sequencing data are available in the Supplemental Table.

### Data Availability

The following information was supplied regarding data availability: The raw data are available in the Supplemental File.
