# Supplementary figures and images for: FGF19 promotes cell autophagy and cisplatin chemoresistance by activating MAPK signaling in ovarian cancer
Source: PeerJ. 2023 Feb 2;11:e14827. doi: 10.7717/peerj.14827 (PMC9899438; doi:10.7717/peerj.14827)

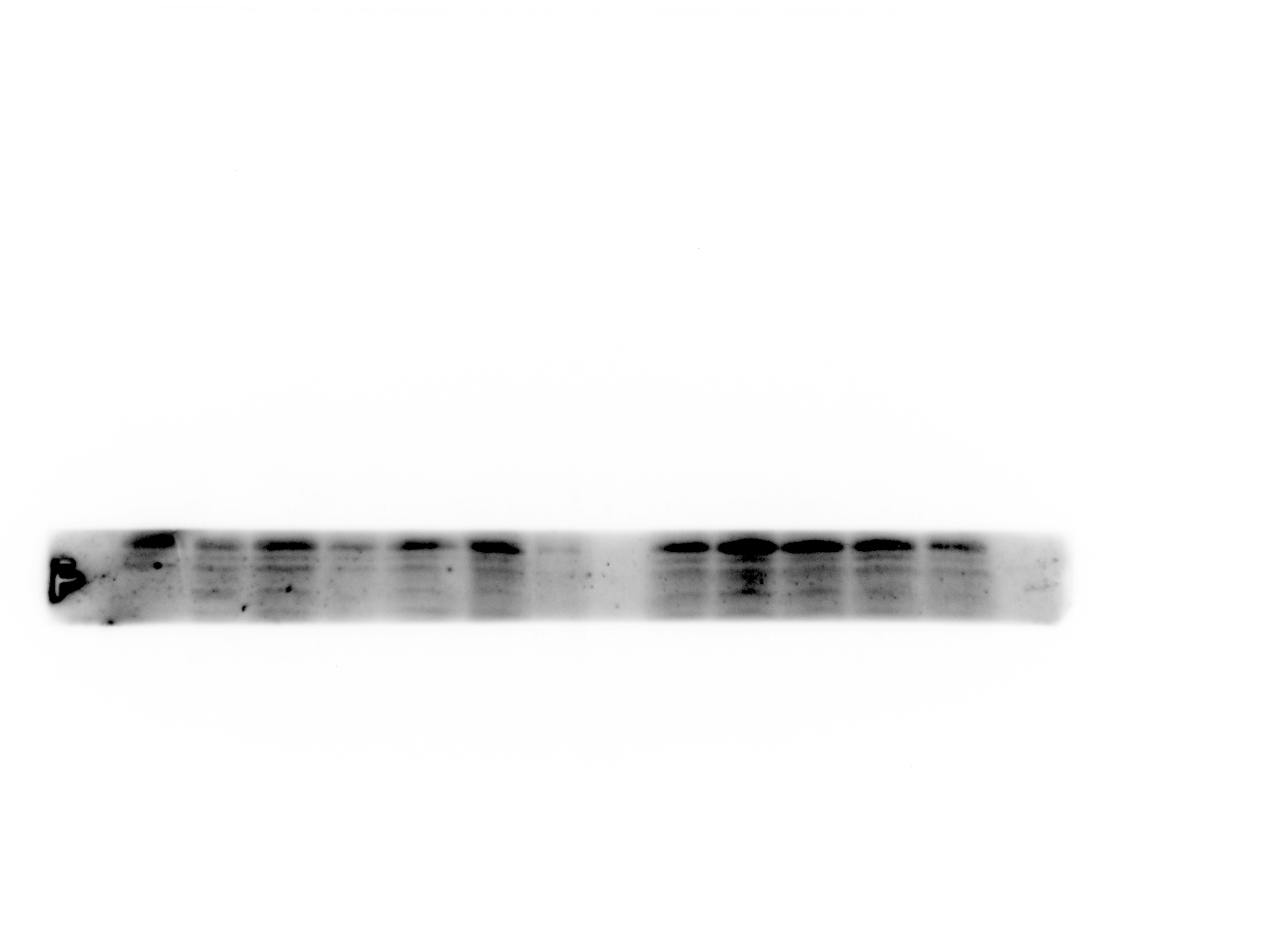

Supplement: Supplemental Information 1 [file peerj-11-14827-s001.zip › Western blot-original gels/Figure 3A-FGF19.tif]

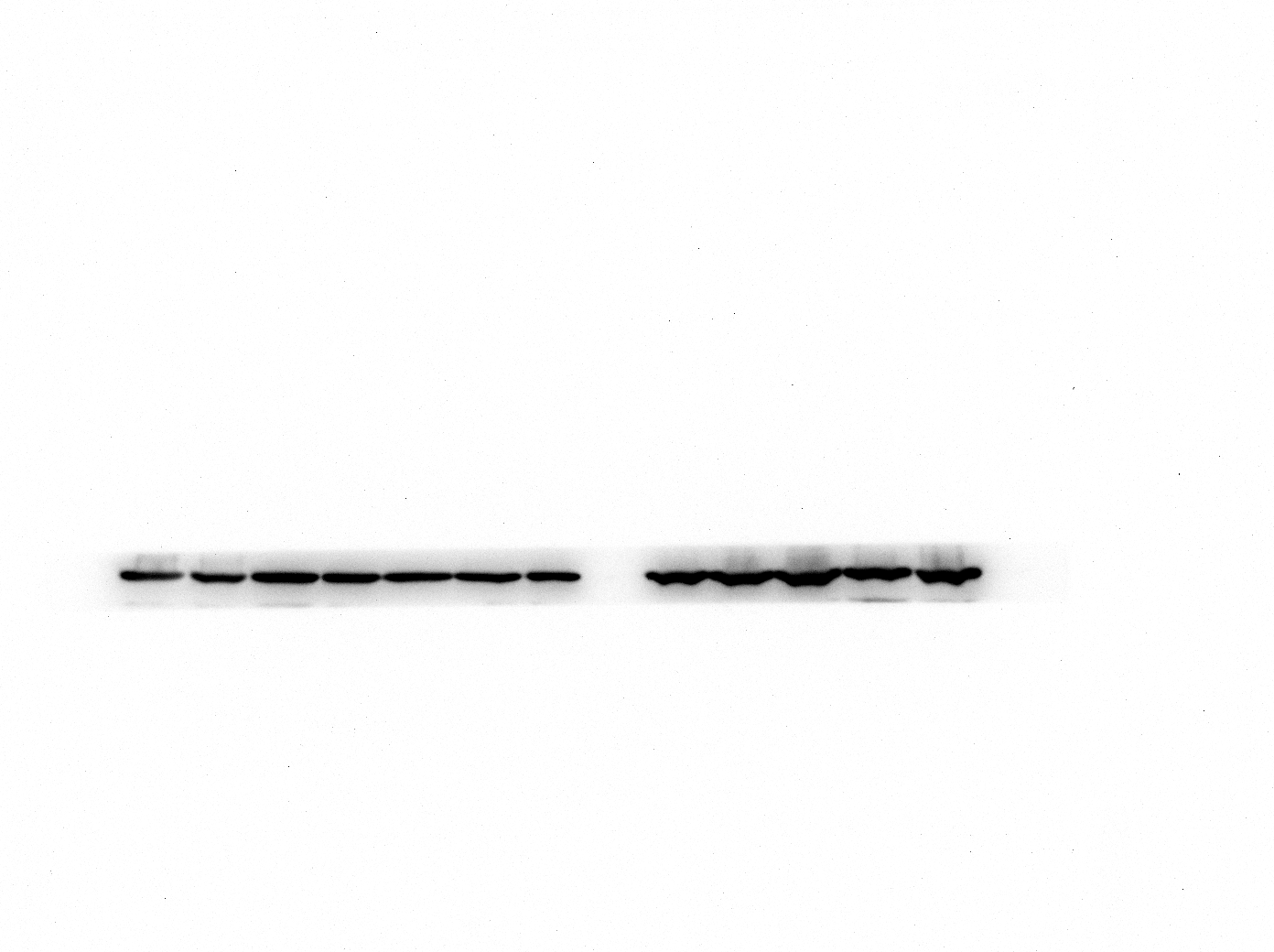

Supplement: Supplemental Information 1 [file peerj-11-14827-s001.zip › Western blot-original gels/Figure 3A-tubulin.tif]

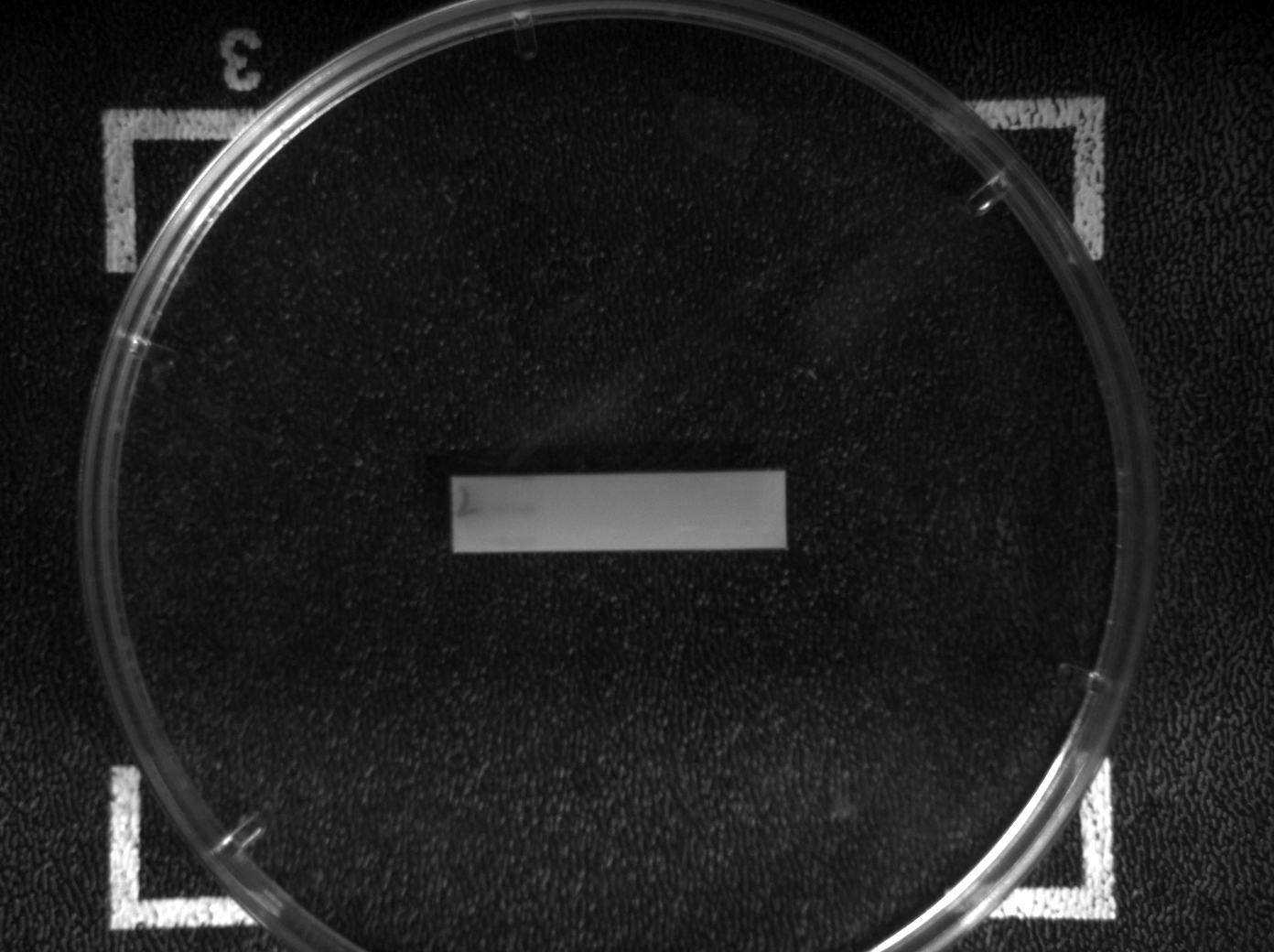

Supplement: Supplemental Information 1 [file peerj-11-14827-s001.zip › Western blot-original gels/Figure 3C-Beclin1-white light.tif]

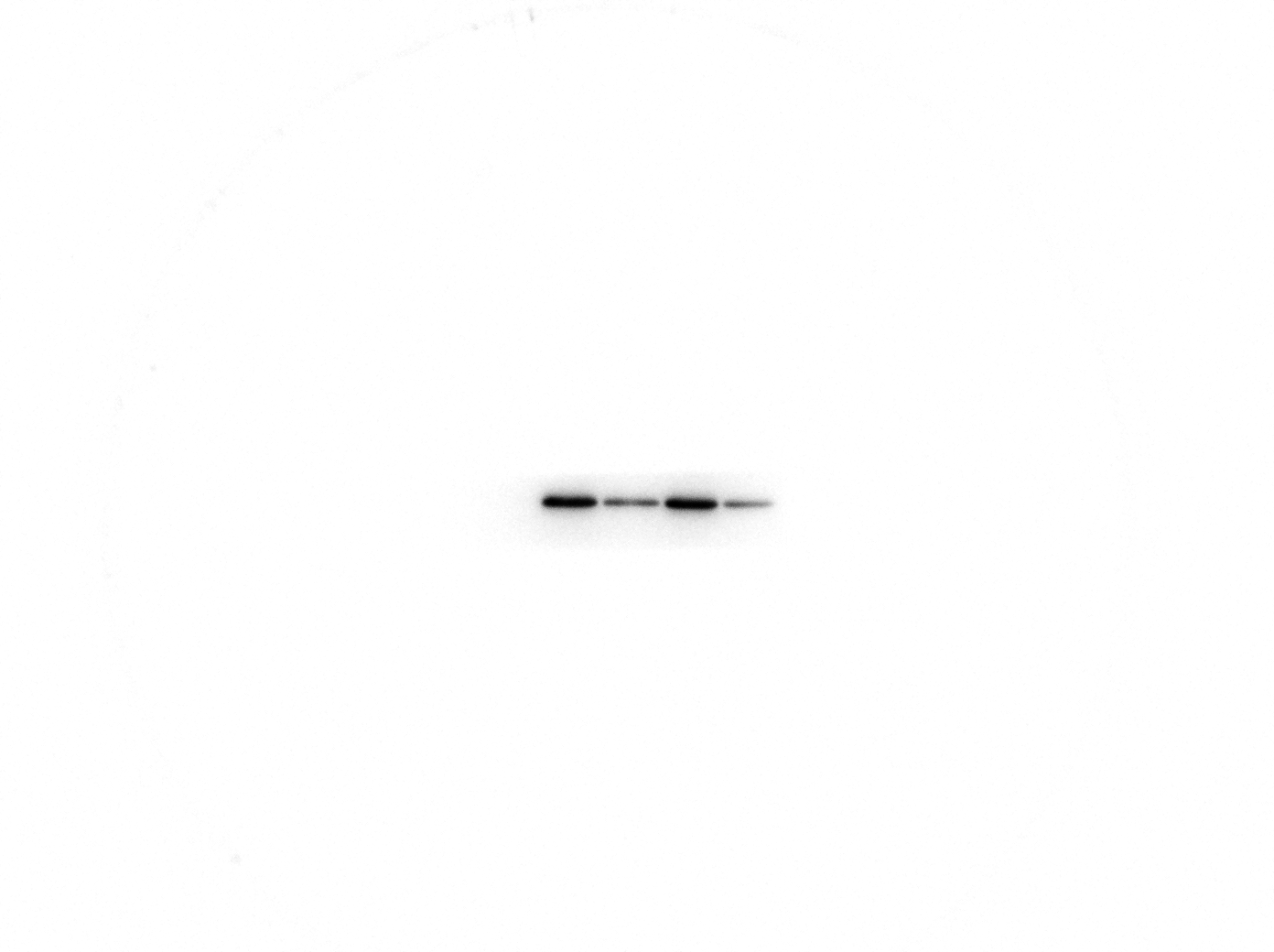

Supplement: Supplemental Information 1 [file peerj-11-14827-s001.zip › Western blot-original gels/Figure 3C-Beclin1.tif]

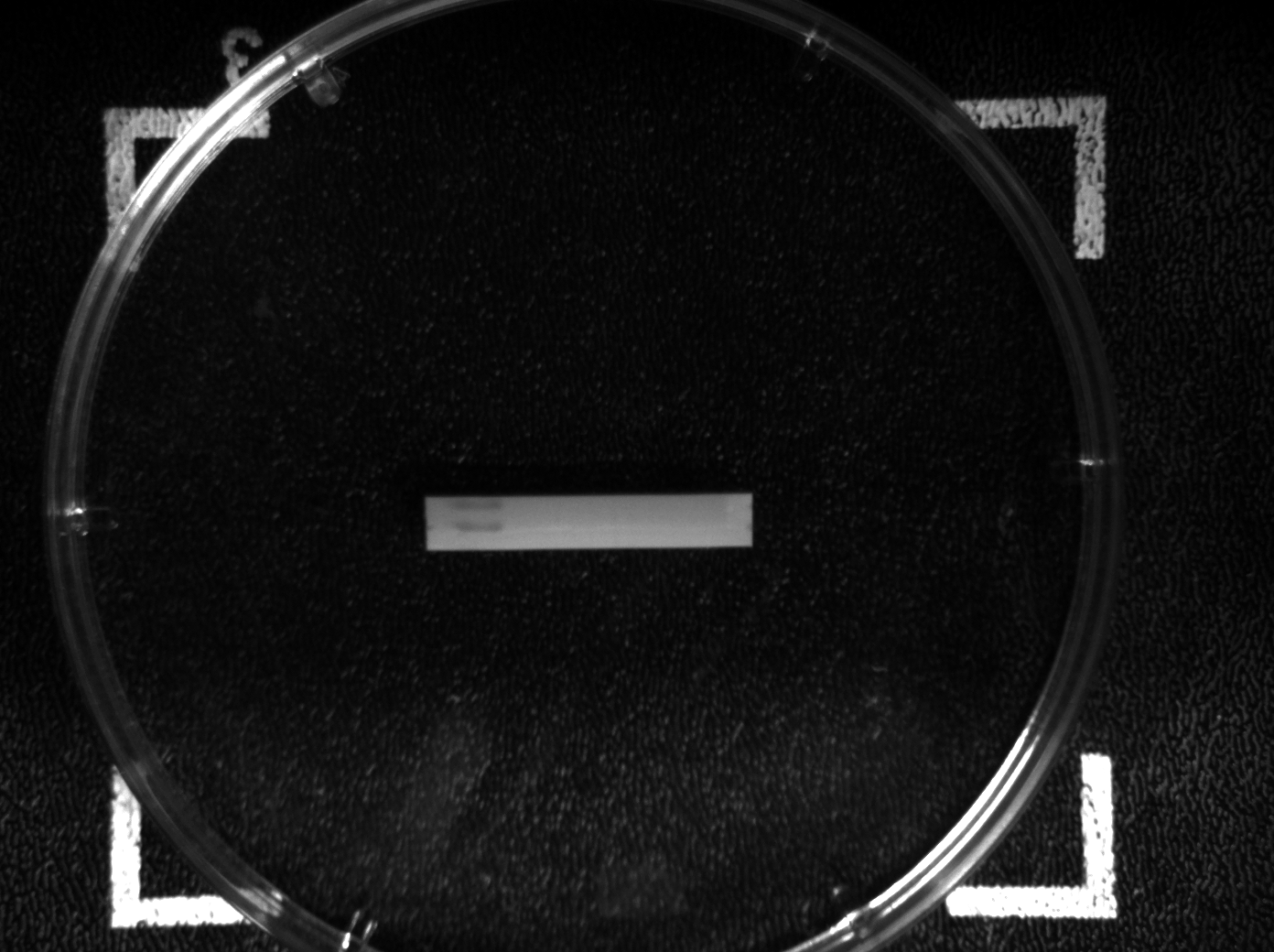

Supplement: Supplemental Information 1 [file peerj-11-14827-s001.zip › Western blot-original gels/Figure 3C-FGF19-white light.tif]

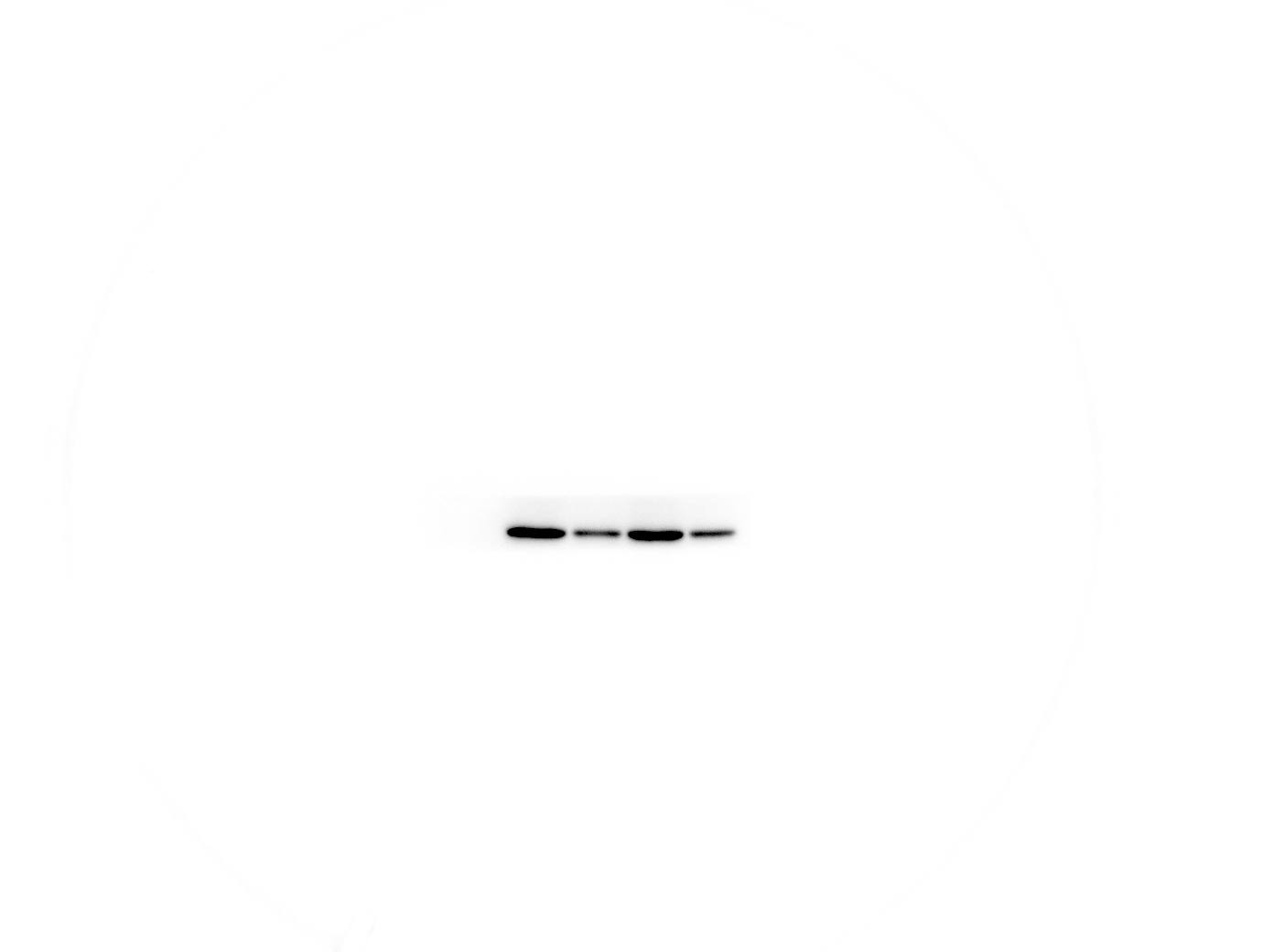

Supplement: Supplemental Information 1 [file peerj-11-14827-s001.zip › Western blot-original gels/Figure 3C-FGF19.tif]

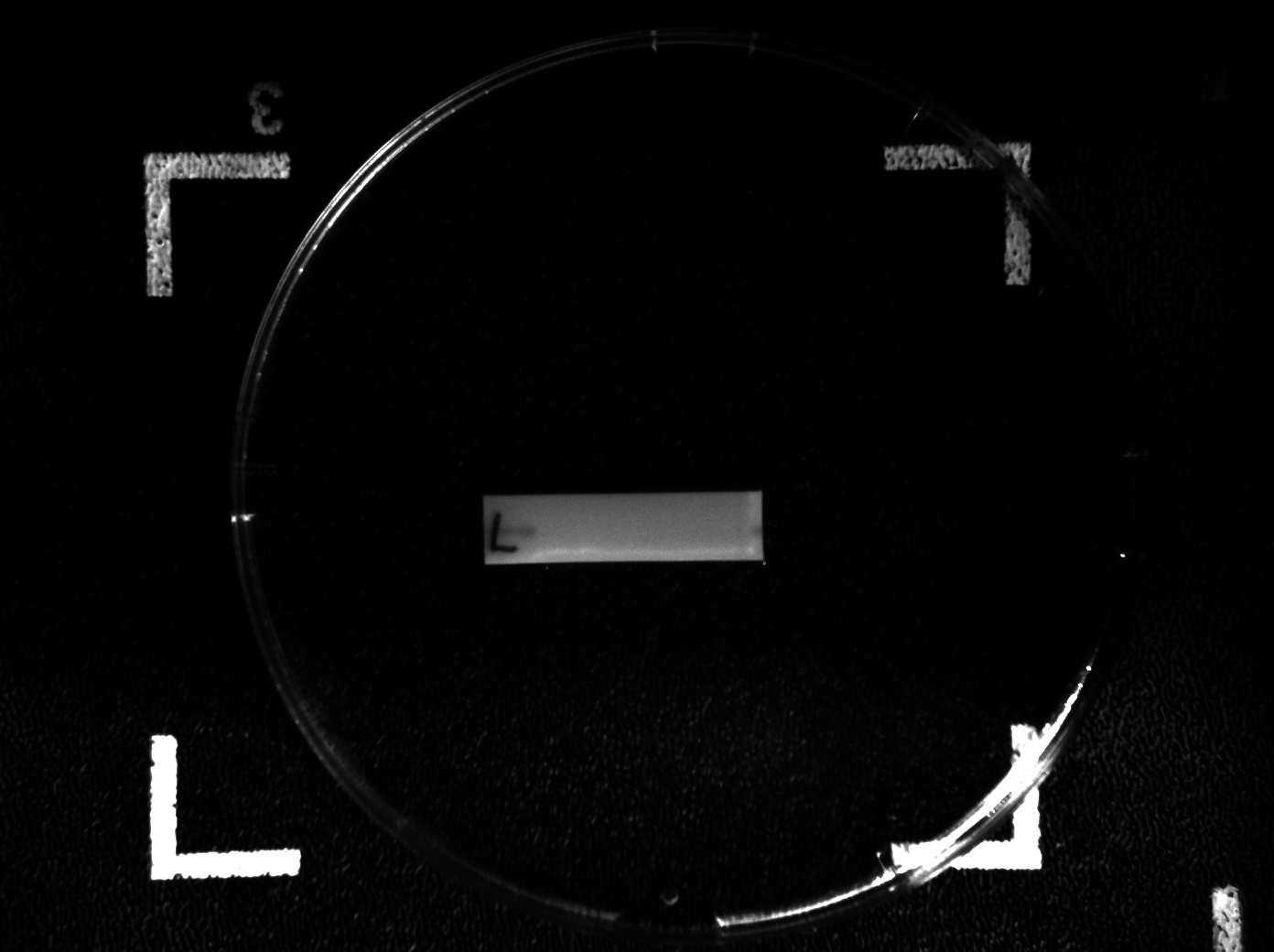

Supplement: Supplemental Information 1 [file peerj-11-14827-s001.zip › Western blot-original gels/Figure 3C-LC3-white light.tif]

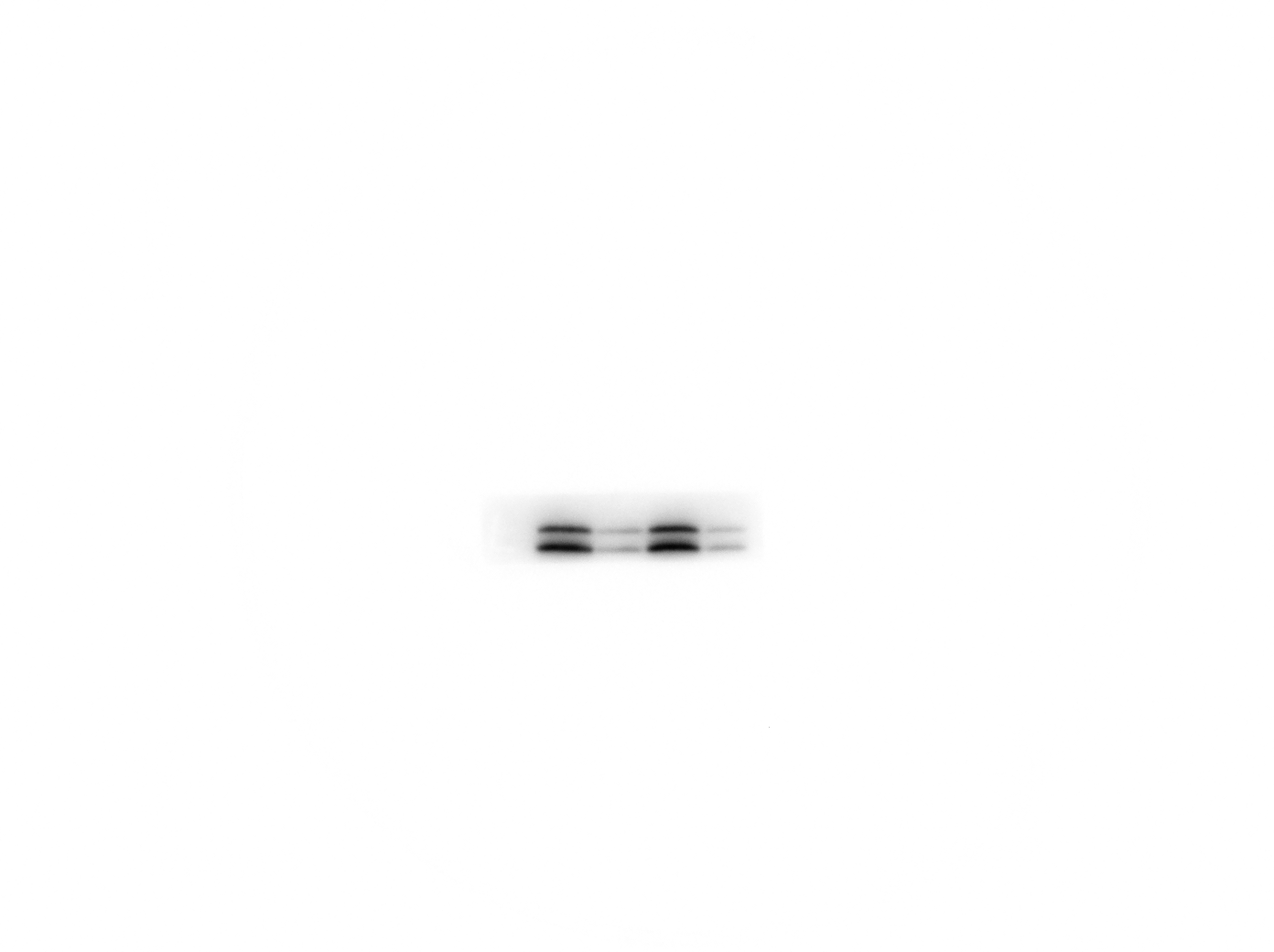

Supplement: Supplemental Information 1 [file peerj-11-14827-s001.zip › Western blot-original gels/Figure 3C-LC3.tif]

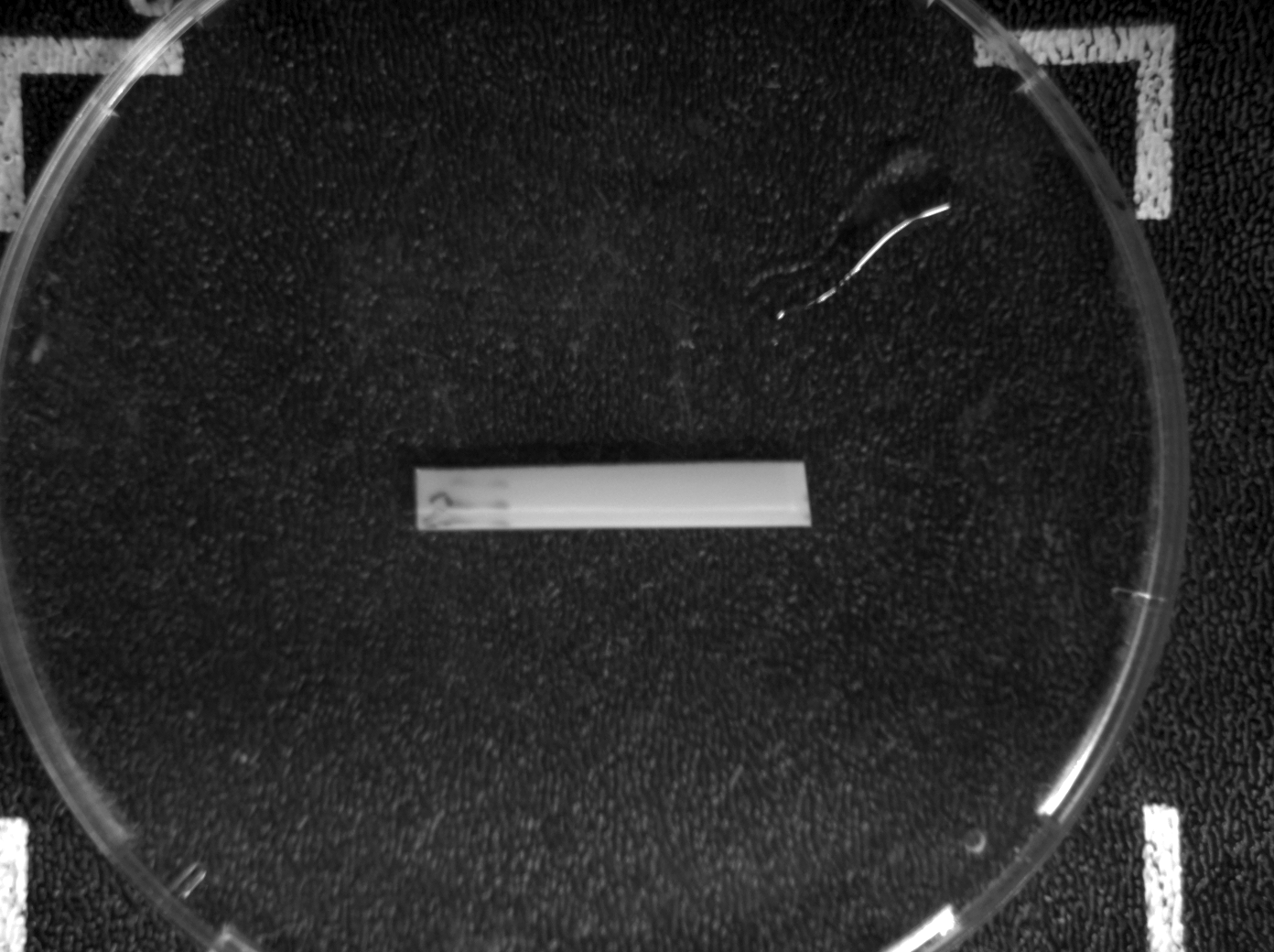

Supplement: Supplemental Information 1 [file peerj-11-14827-s001.zip › Western blot-original gels/Figure 3C-Tubulin-white light.tif]

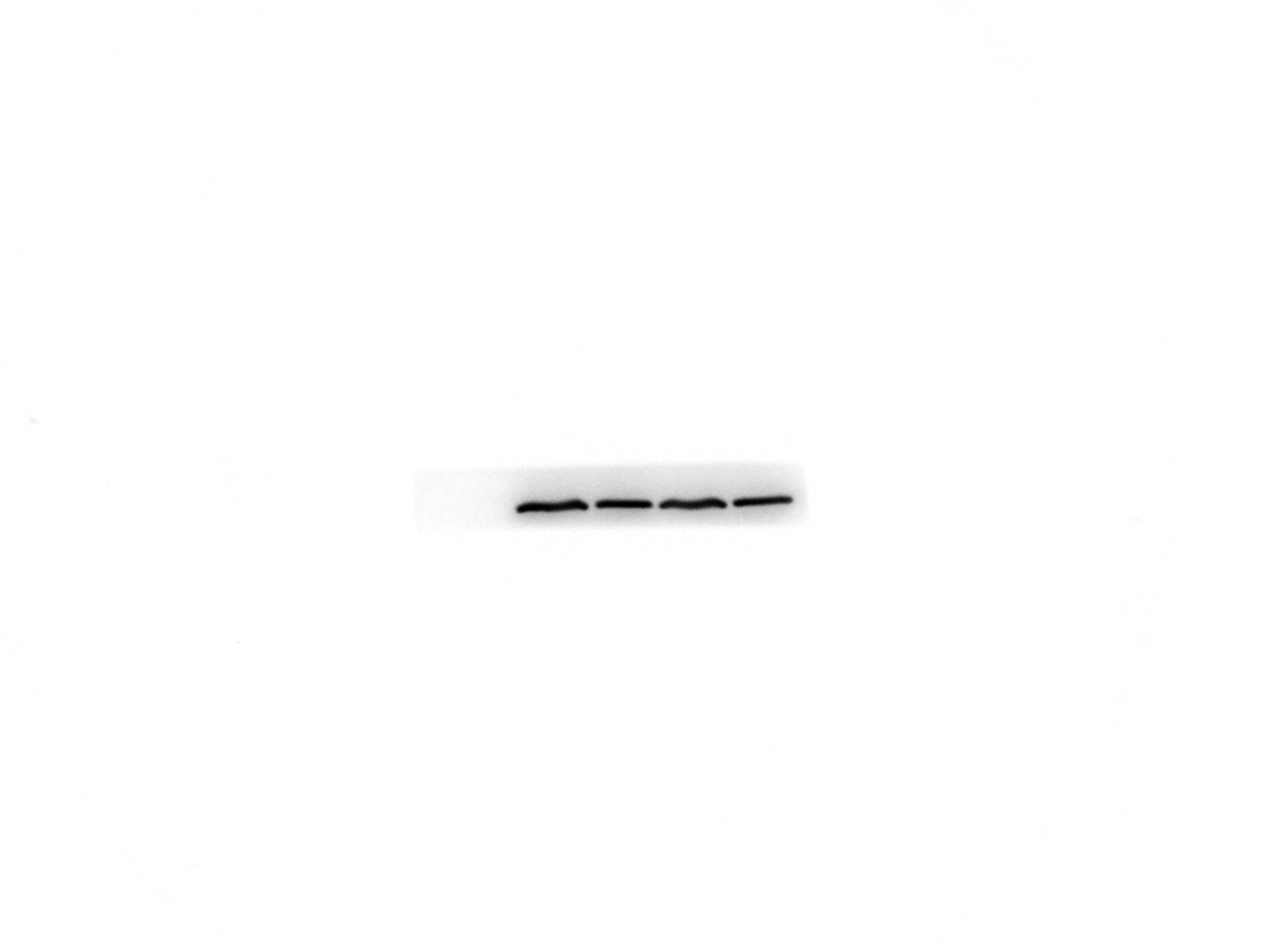

Supplement: Supplemental Information 1 [file peerj-11-14827-s001.zip › Western blot-original gels/Figure 3C-Tubulin.tif]

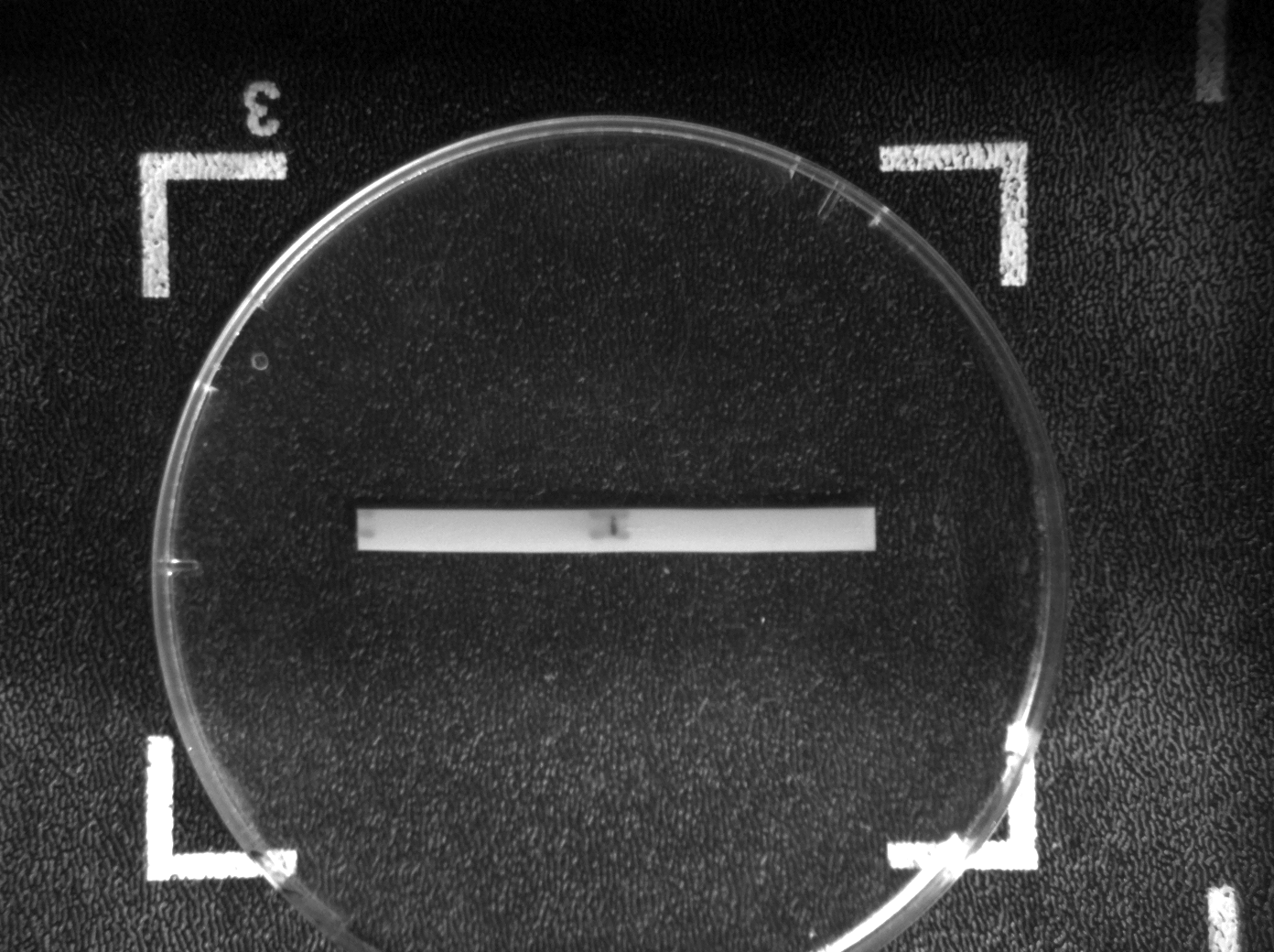

Supplement: Supplemental Information 1 [file peerj-11-14827-s001.zip › Western blot-original gels/Figure 3C-p62-white light.tif]

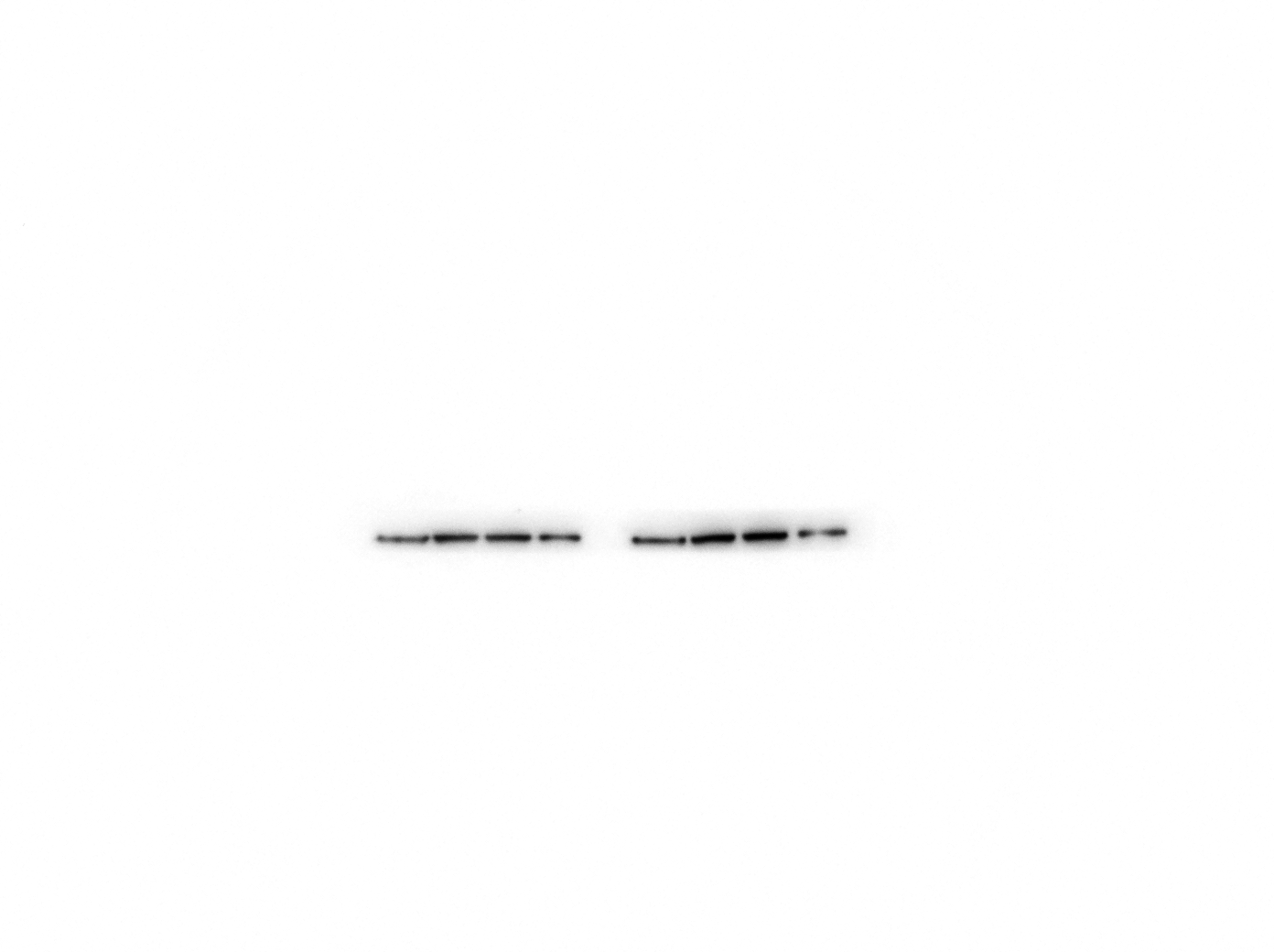

Supplement: Supplemental Information 1 [file peerj-11-14827-s001.zip › Western blot-original gels/Figure 3C-p62.tif]

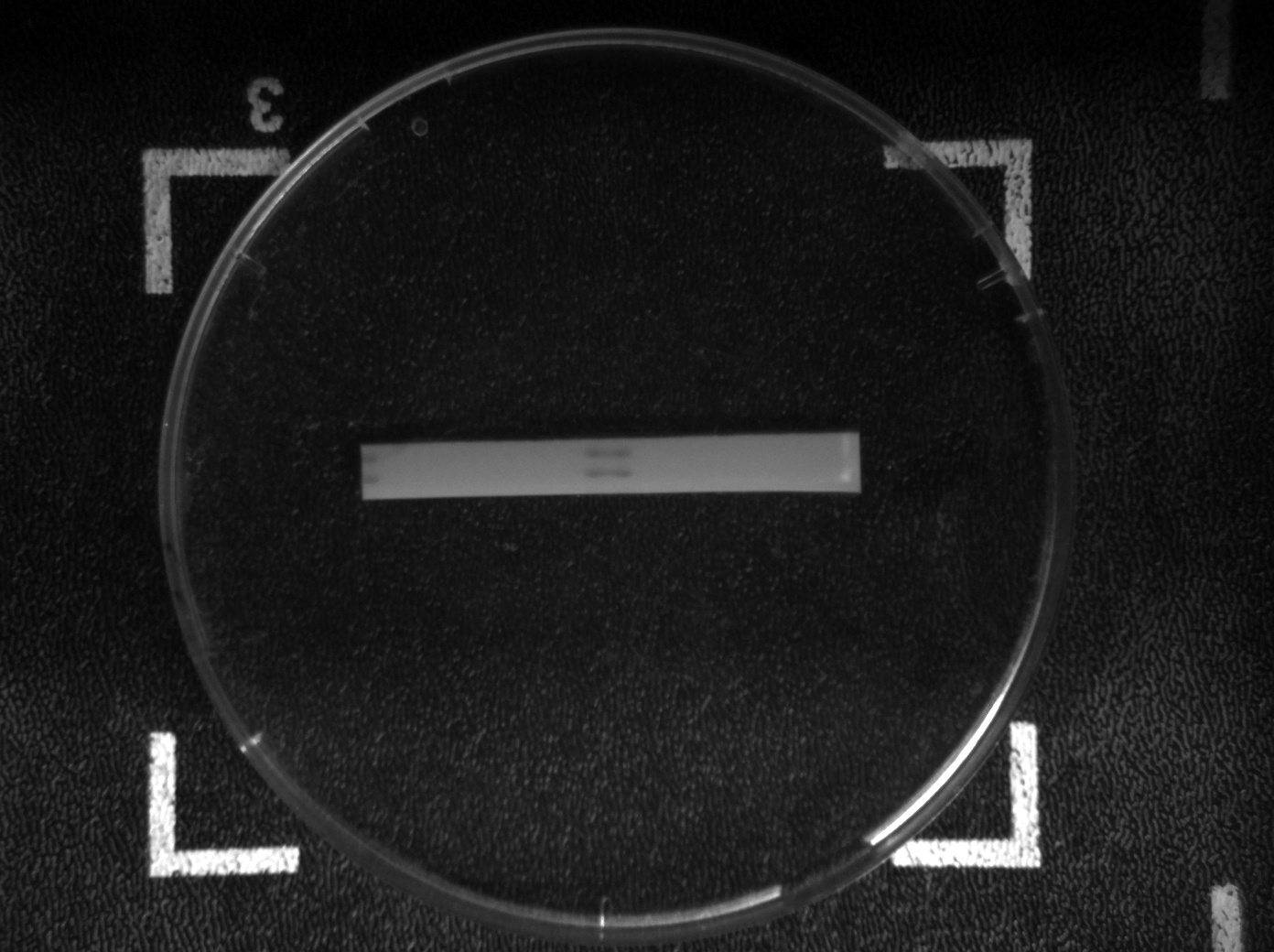

Supplement: Supplemental Information 1 [file peerj-11-14827-s001.zip › Western blot-original gels/Figure 4B-Beclin1-white light.tif]

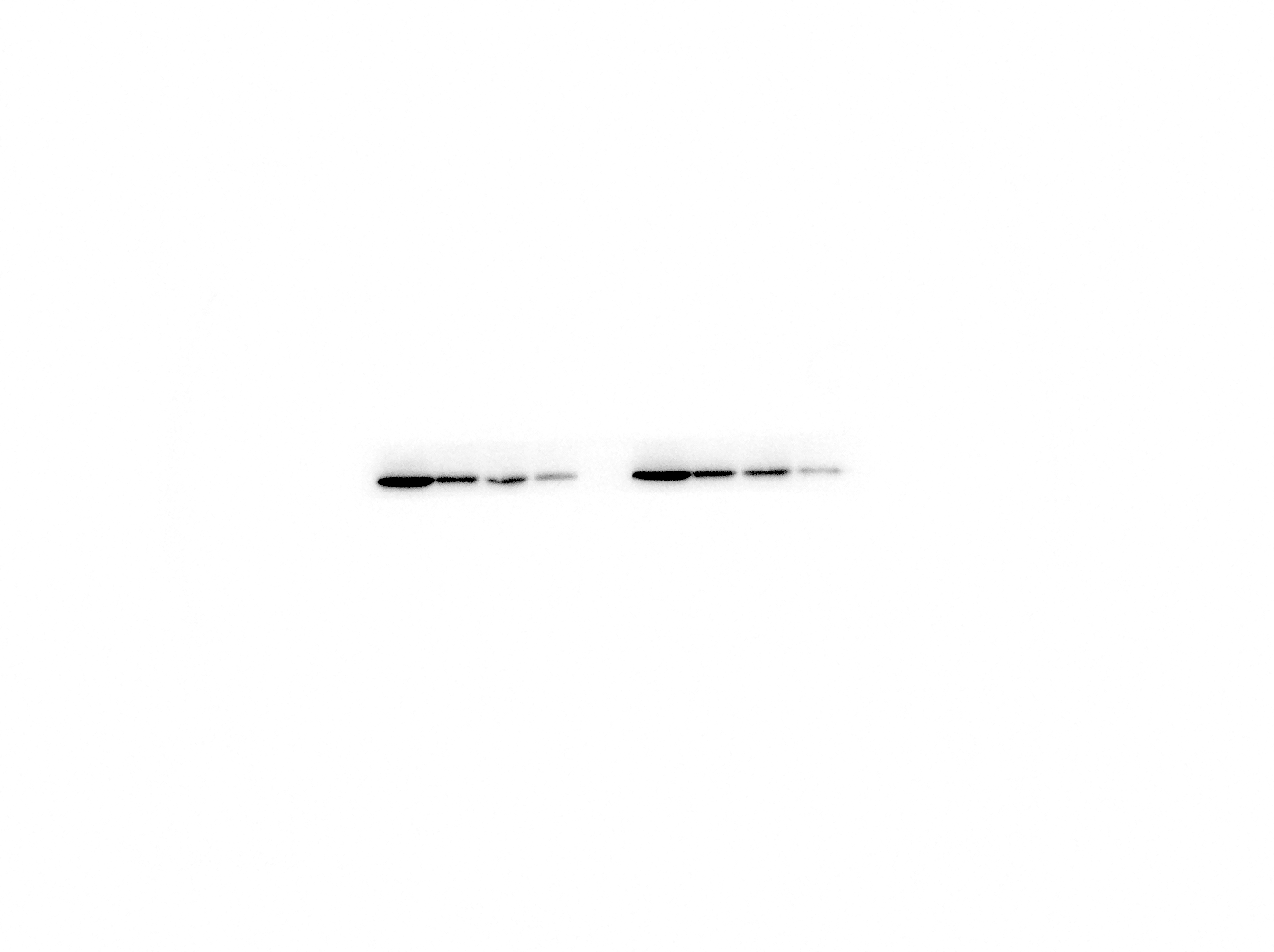

Supplement: Supplemental Information 1 [file peerj-11-14827-s001.zip › Western blot-original gels/Figure 4B-Beclin1.tif]

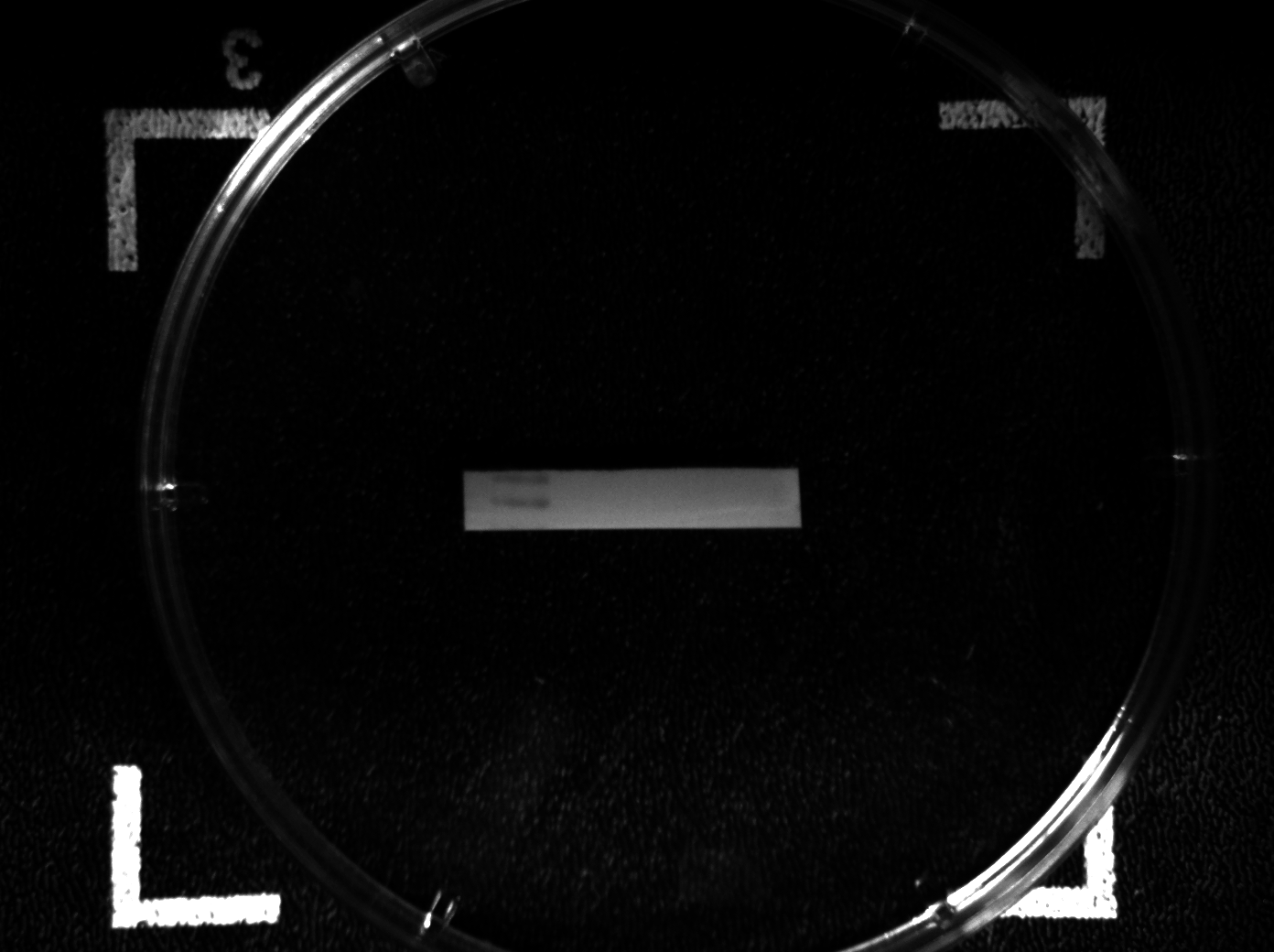

Supplement: Supplemental Information 1 [file peerj-11-14827-s001.zip › Western blot-original gels/Figure 4B-FGF19(HO8910pm)-white light.tif]

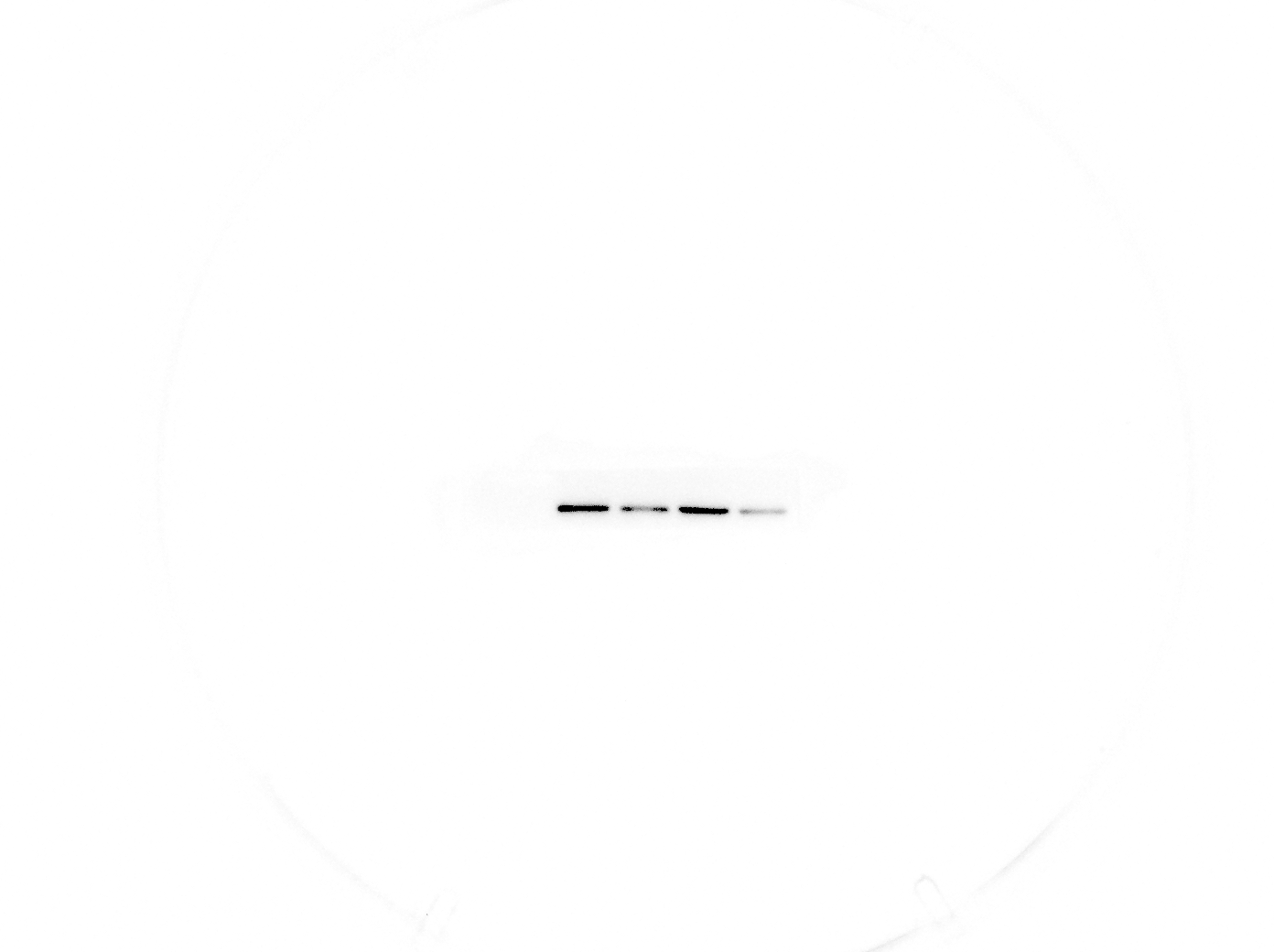

Supplement: Supplemental Information 1 [file peerj-11-14827-s001.zip › Western blot-original gels/Figure 4B-FGF19(HO8910pm).tif]

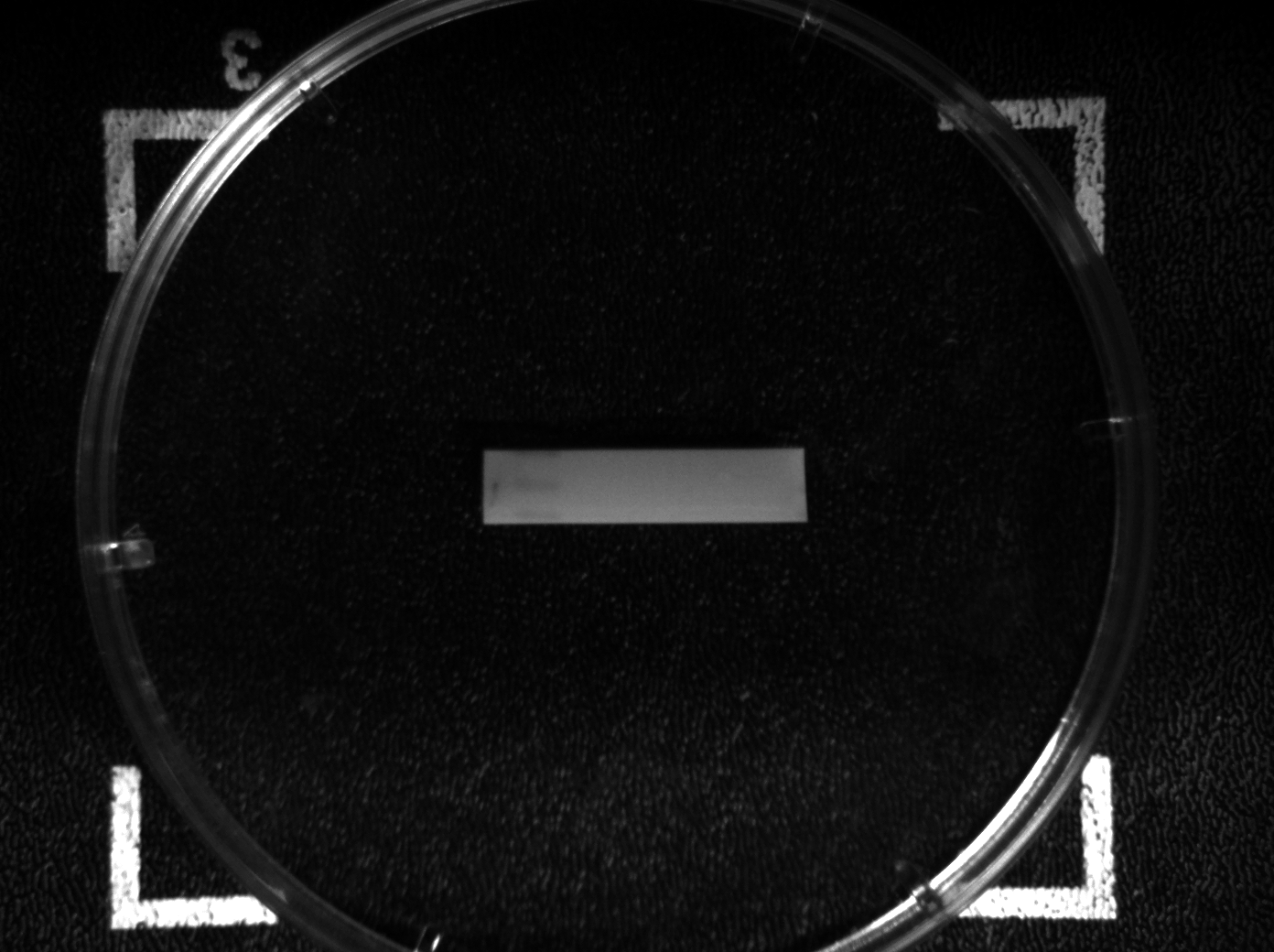

Supplement: Supplemental Information 1 [file peerj-11-14827-s001.zip › Western blot-original gels/Figure 4B-FGF19(SKOV3-IP)-white light.tif]

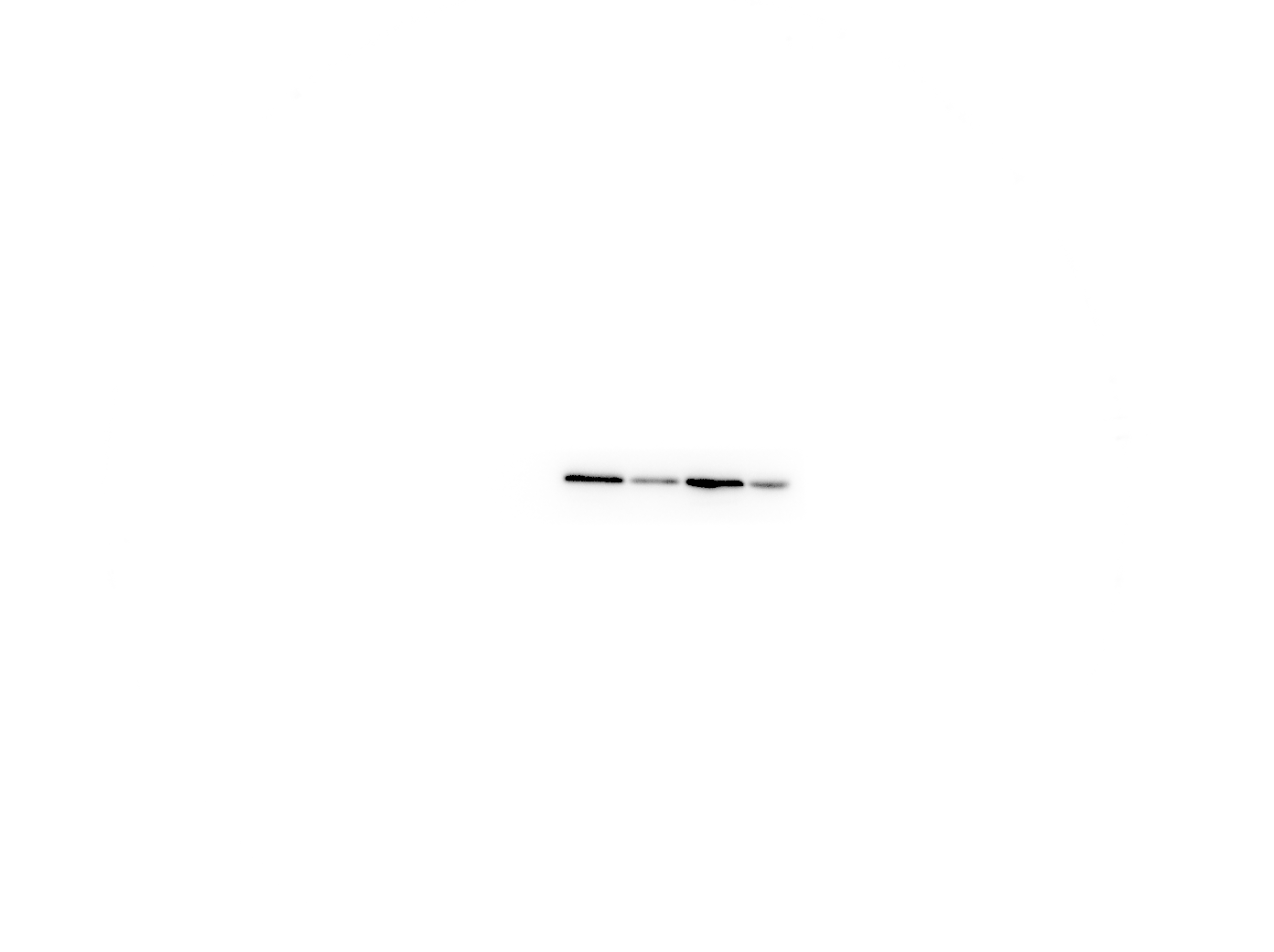

Supplement: Supplemental Information 1 [file peerj-11-14827-s001.zip › Western blot-original gels/Figure 4B-FGF19(SKOV3-IP).tif]

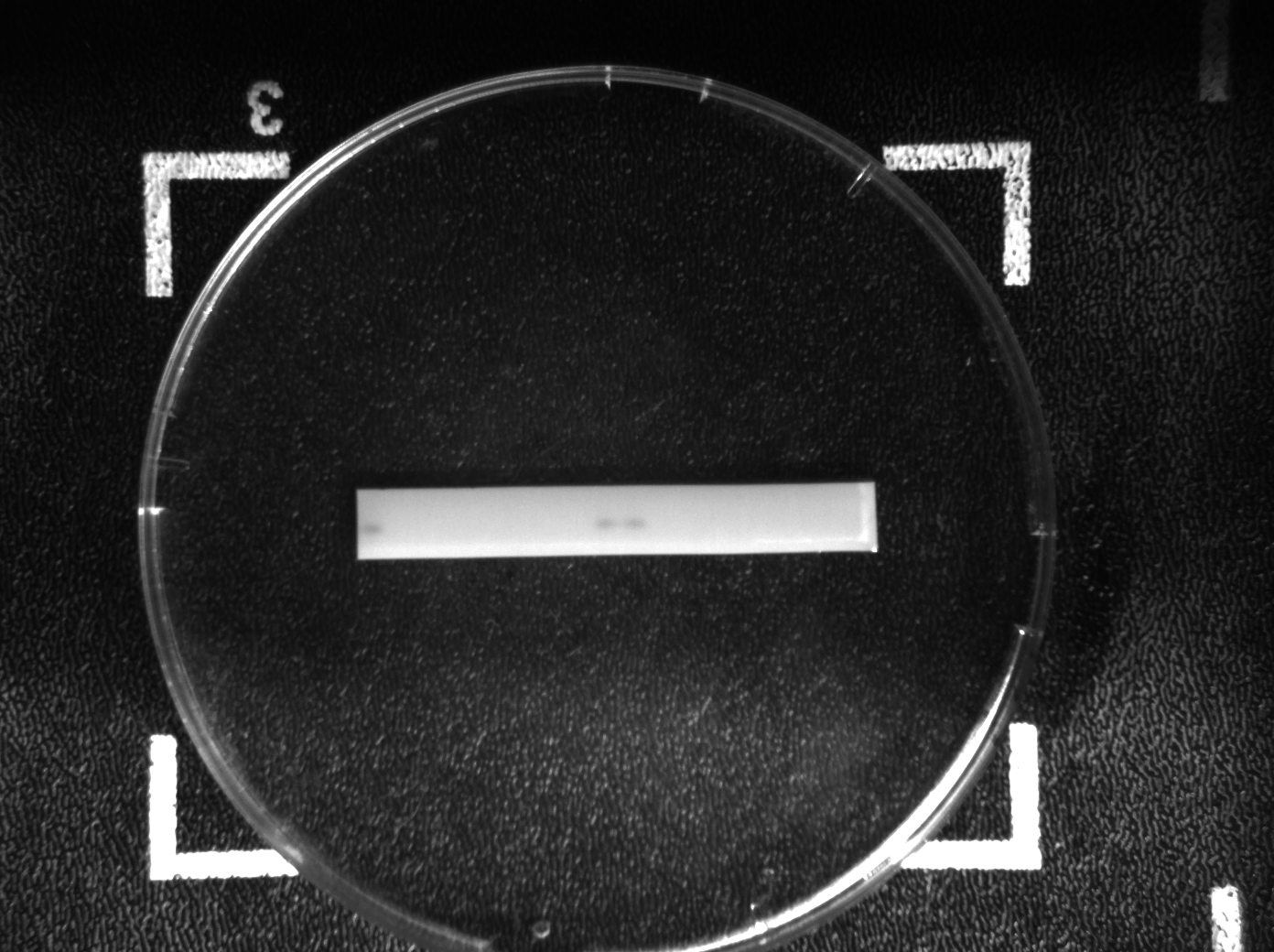

Supplement: Supplemental Information 1 [file peerj-11-14827-s001.zip › Western blot-original gels/Figure 4B-LC3-white light.tif]

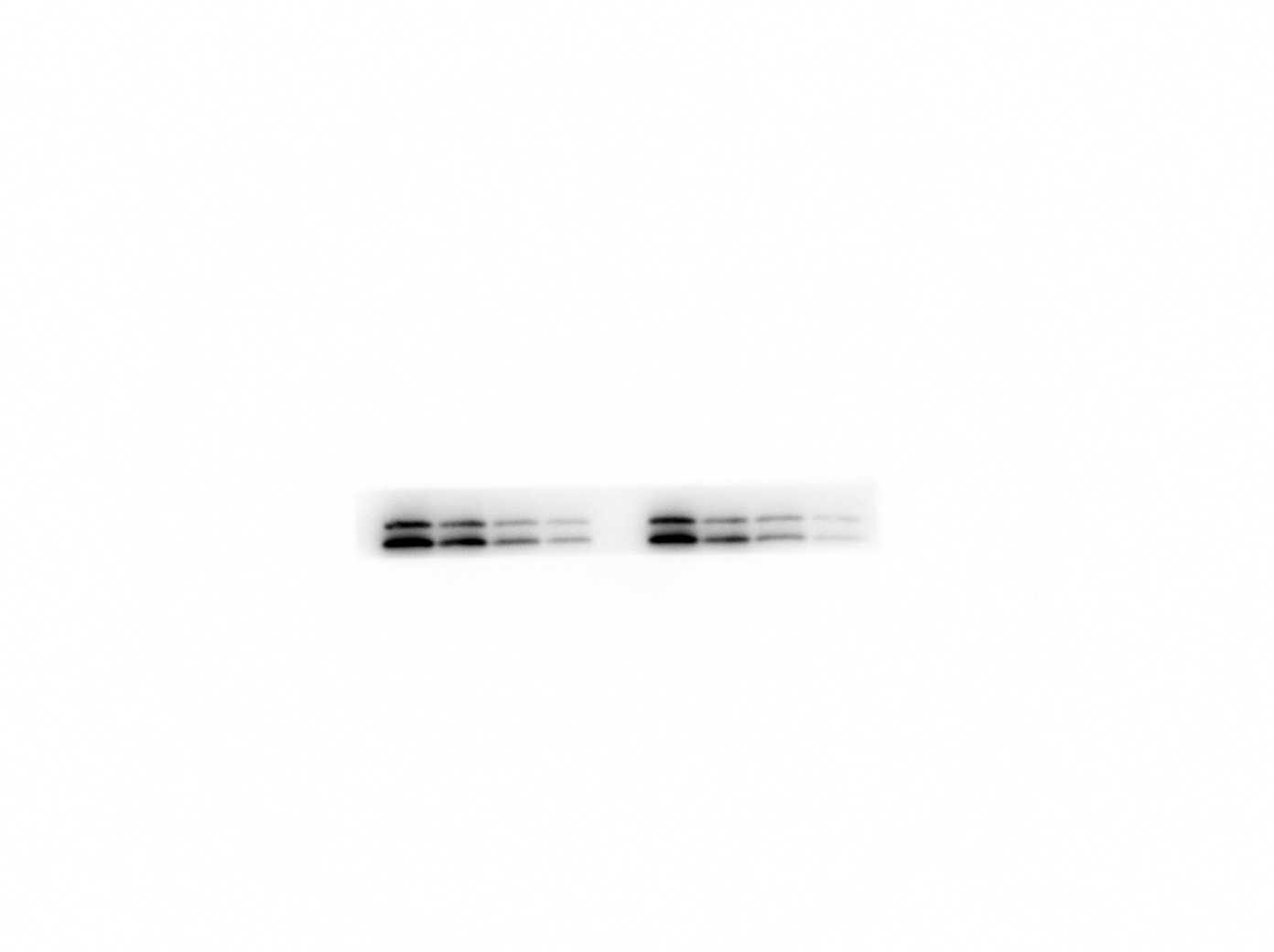

Supplement: Supplemental Information 1 [file peerj-11-14827-s001.zip › Western blot-original gels/Figure 4B-LC3.tif]

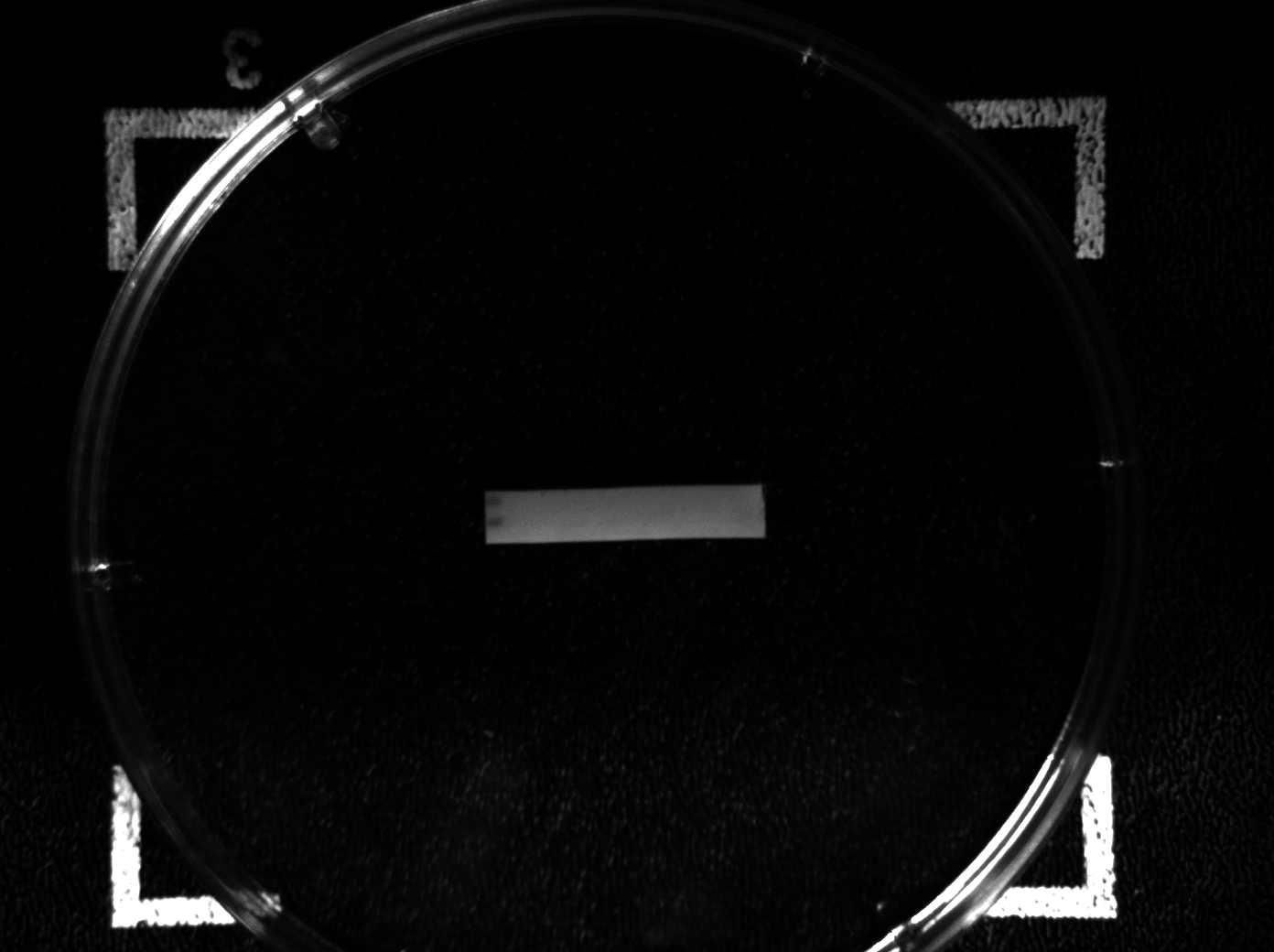

Supplement: Supplemental Information 1 [file peerj-11-14827-s001.zip › Western blot-original gels/Figure 4B-p-p38(HO8910pm)-white light.tif]

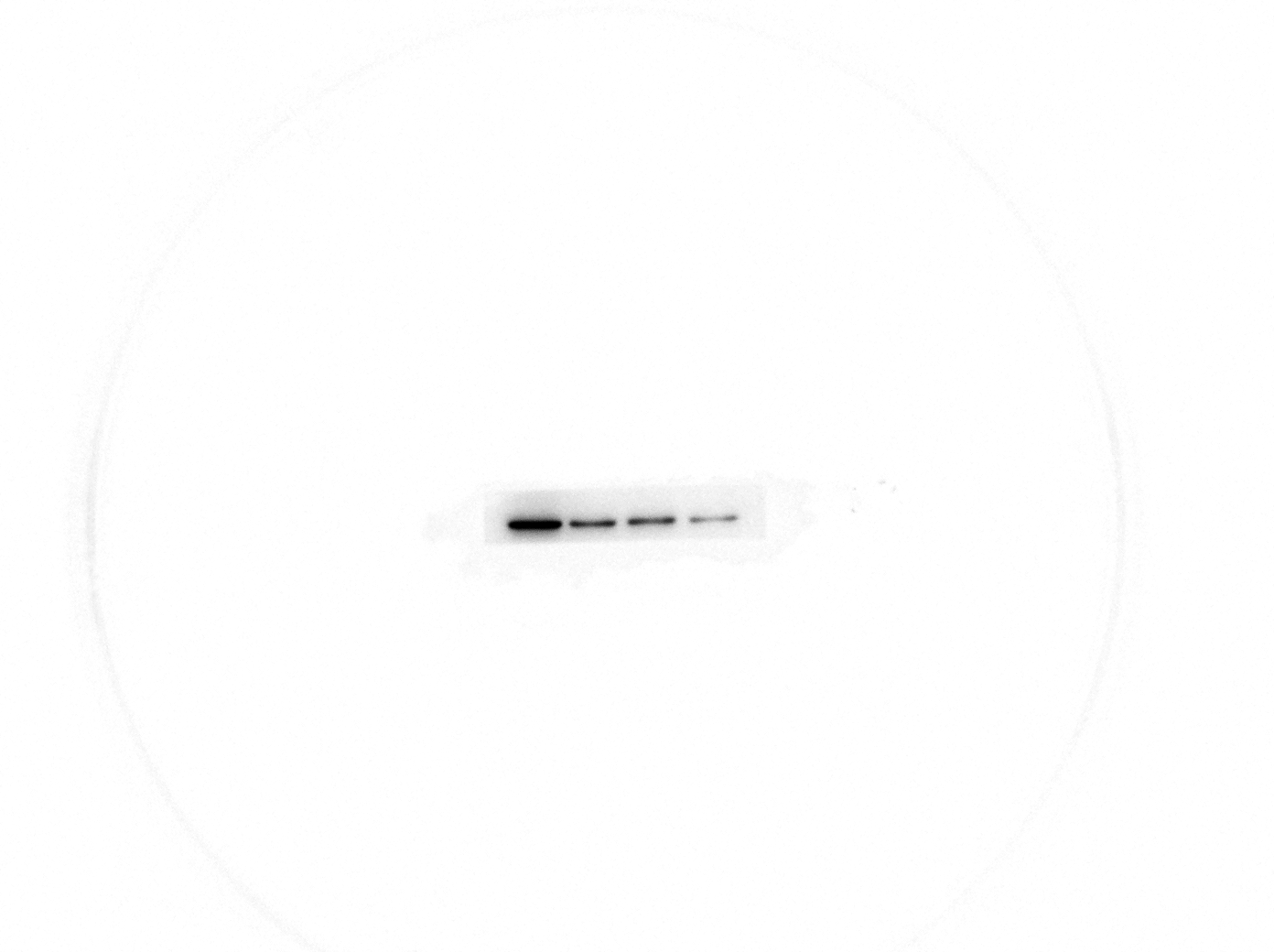

Supplement: Supplemental Information 1 [file peerj-11-14827-s001.zip › Western blot-original gels/Figure 4B-p-p38(HO8910pm).tif]

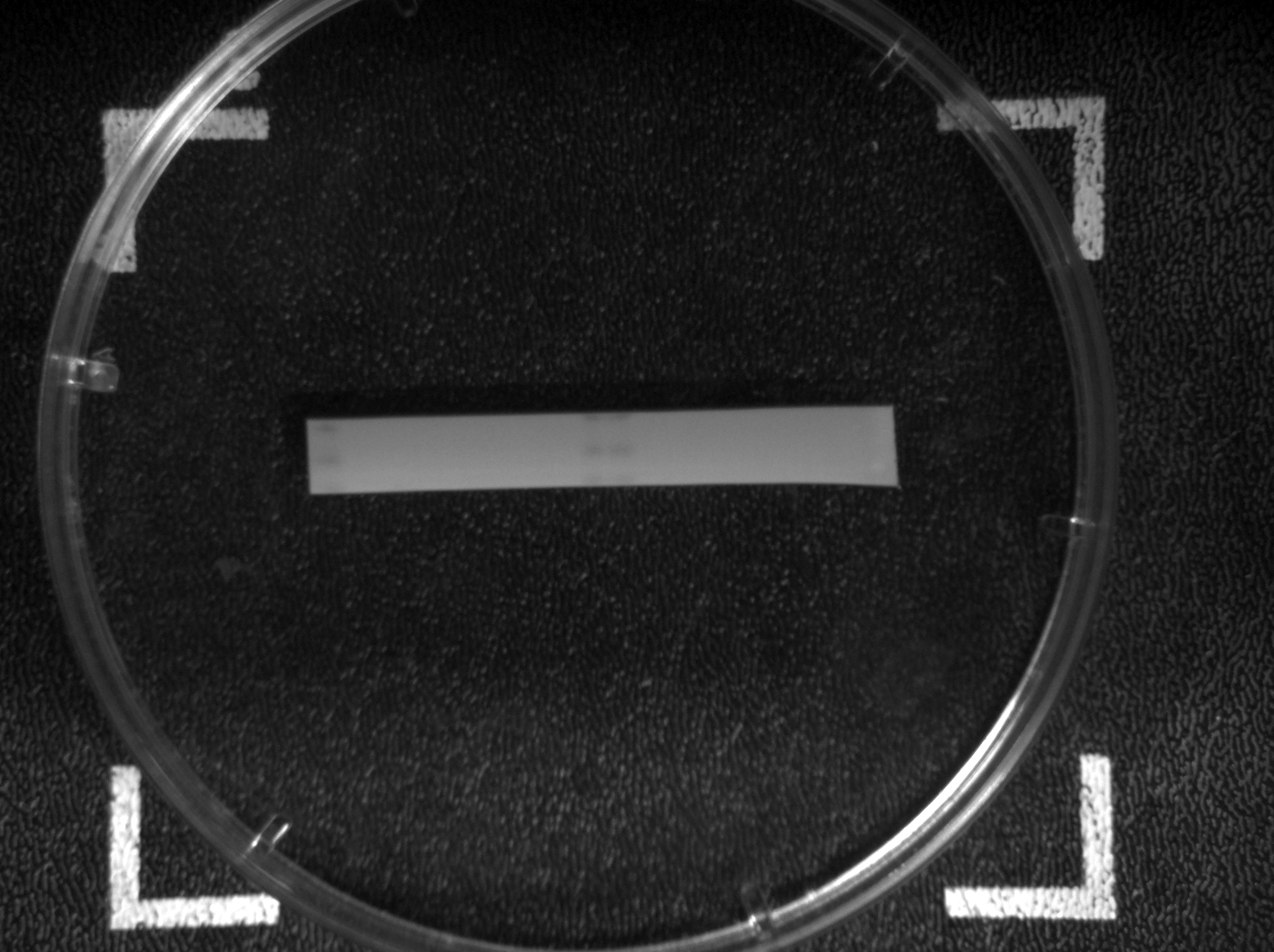

Supplement: Supplemental Information 1 [file peerj-11-14827-s001.zip › Western blot-original gels/Figure 4B-p-p38(SKOV3-IP)-white light.tif]

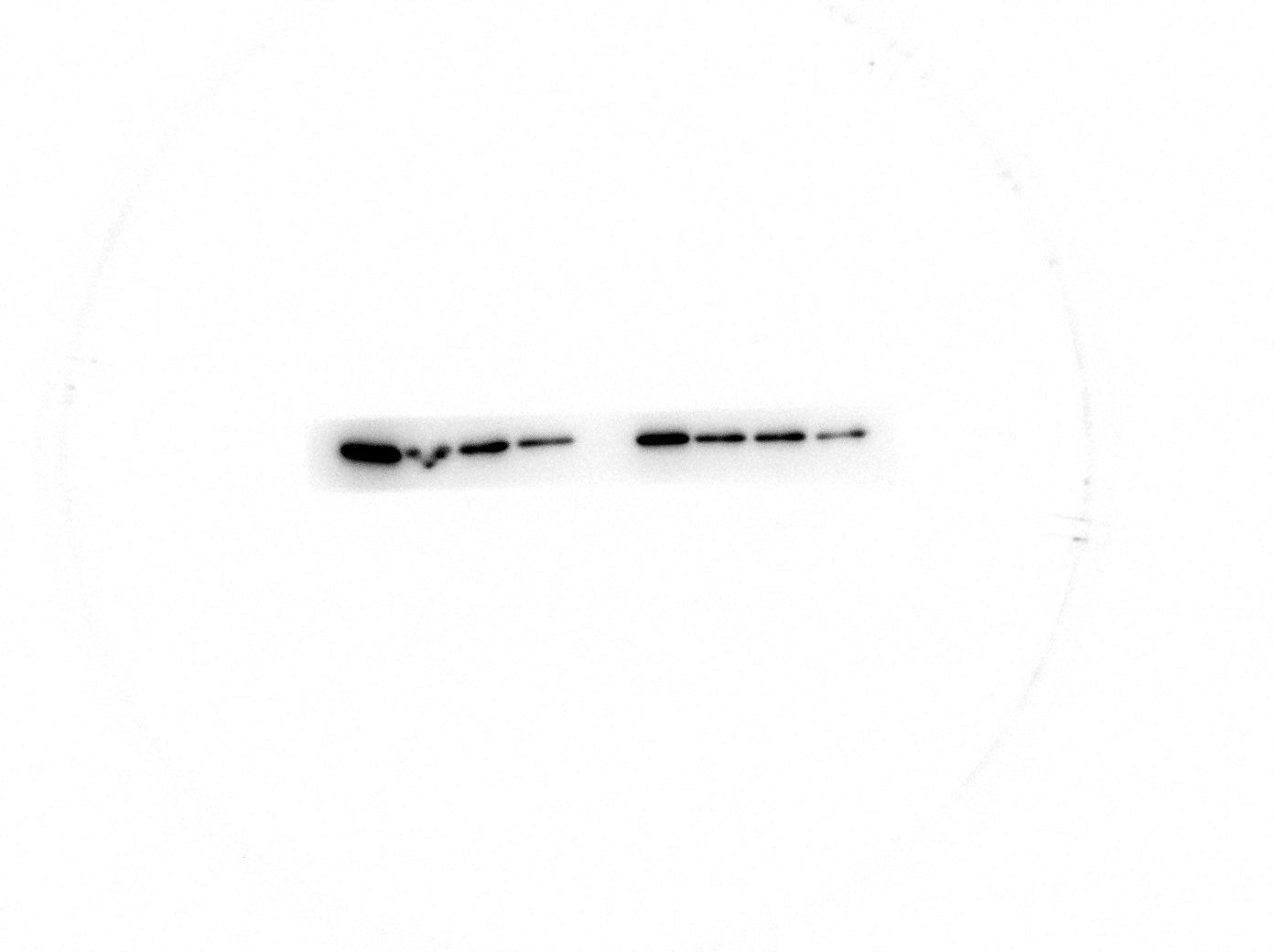

Supplement: Supplemental Information 1 [file peerj-11-14827-s001.zip › Western blot-original gels/Figure 4B-p-p38(SKOV3-IP).tif]

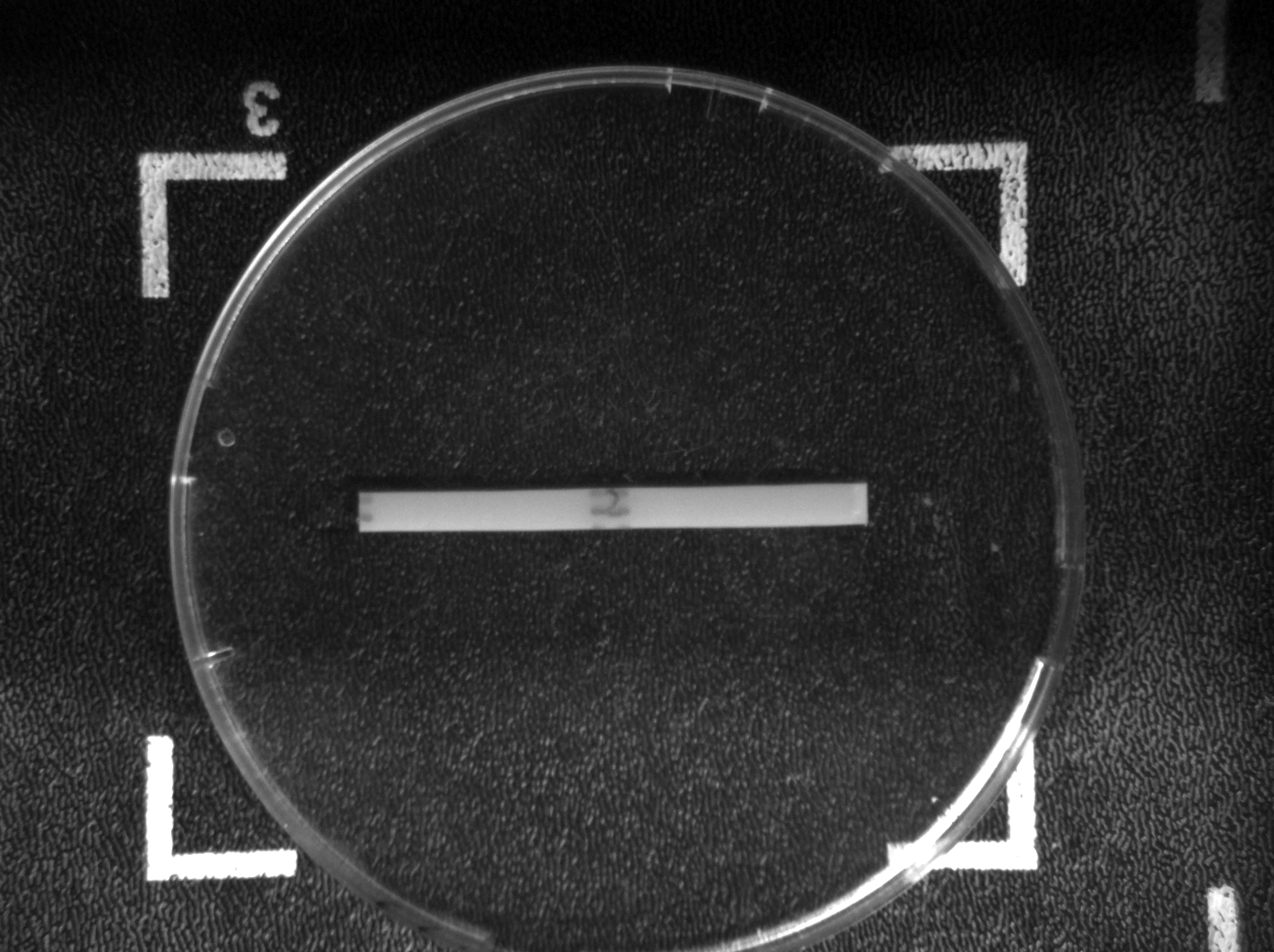

Supplement: Supplemental Information 1 [file peerj-11-14827-s001.zip › Western blot-original gels/Figure 4B-p38-white light.tif]

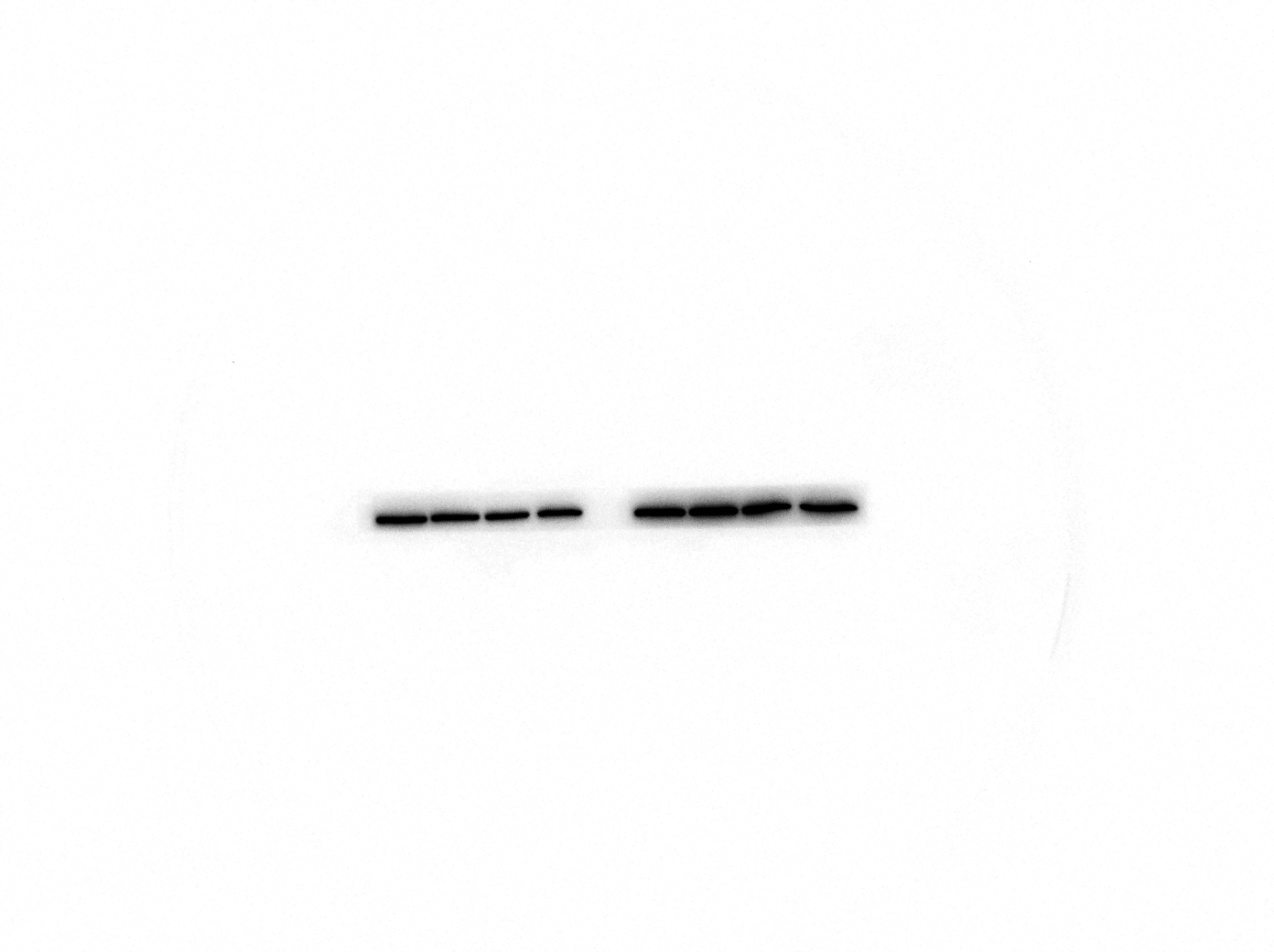

Supplement: Supplemental Information 1 [file peerj-11-14827-s001.zip › Western blot-original gels/Figure 4B-p38.tif]

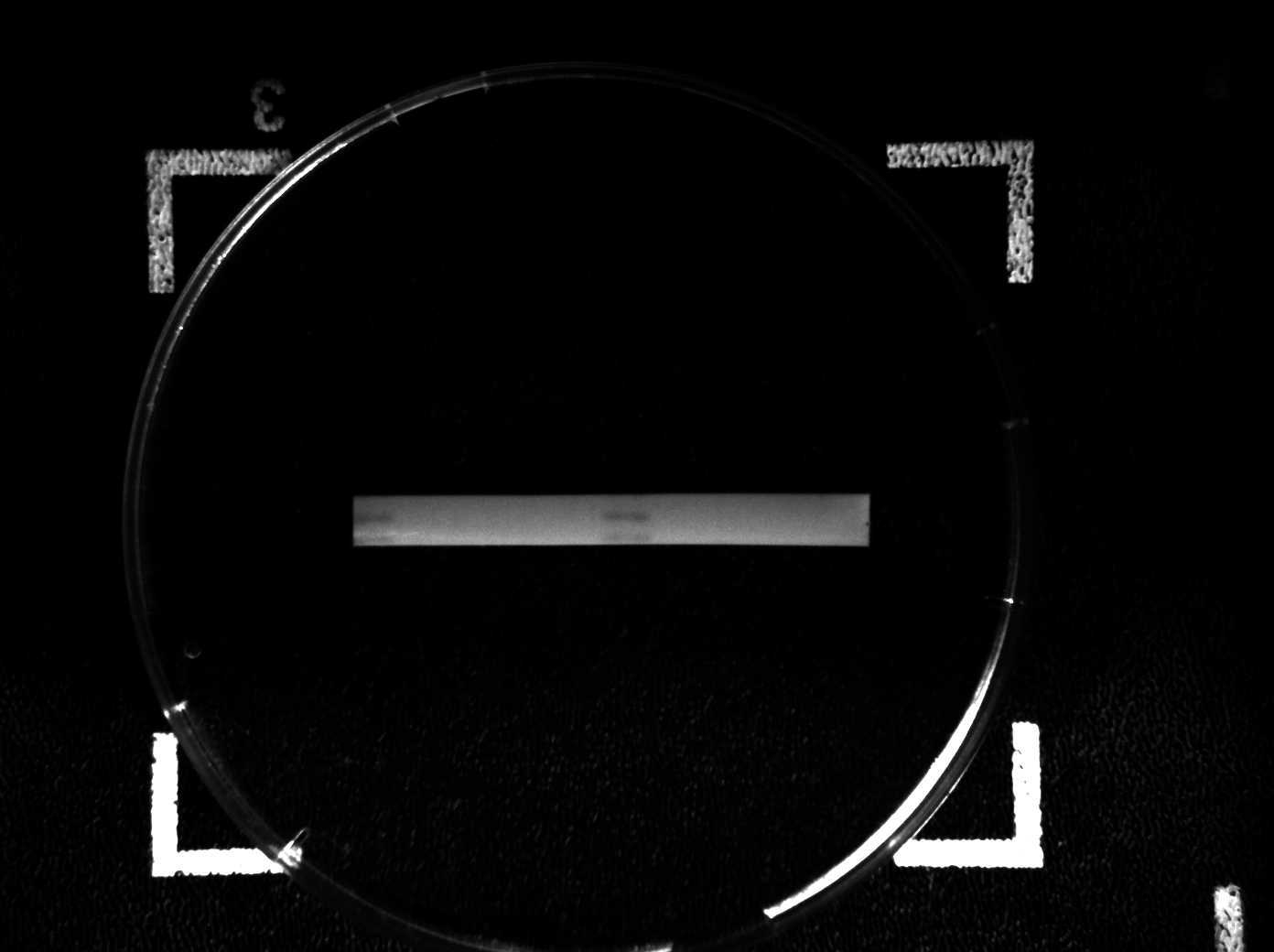

Supplement: Supplemental Information 1 [file peerj-11-14827-s001.zip › Western blot-original gels/Figure 4B-p62-white light.tif]

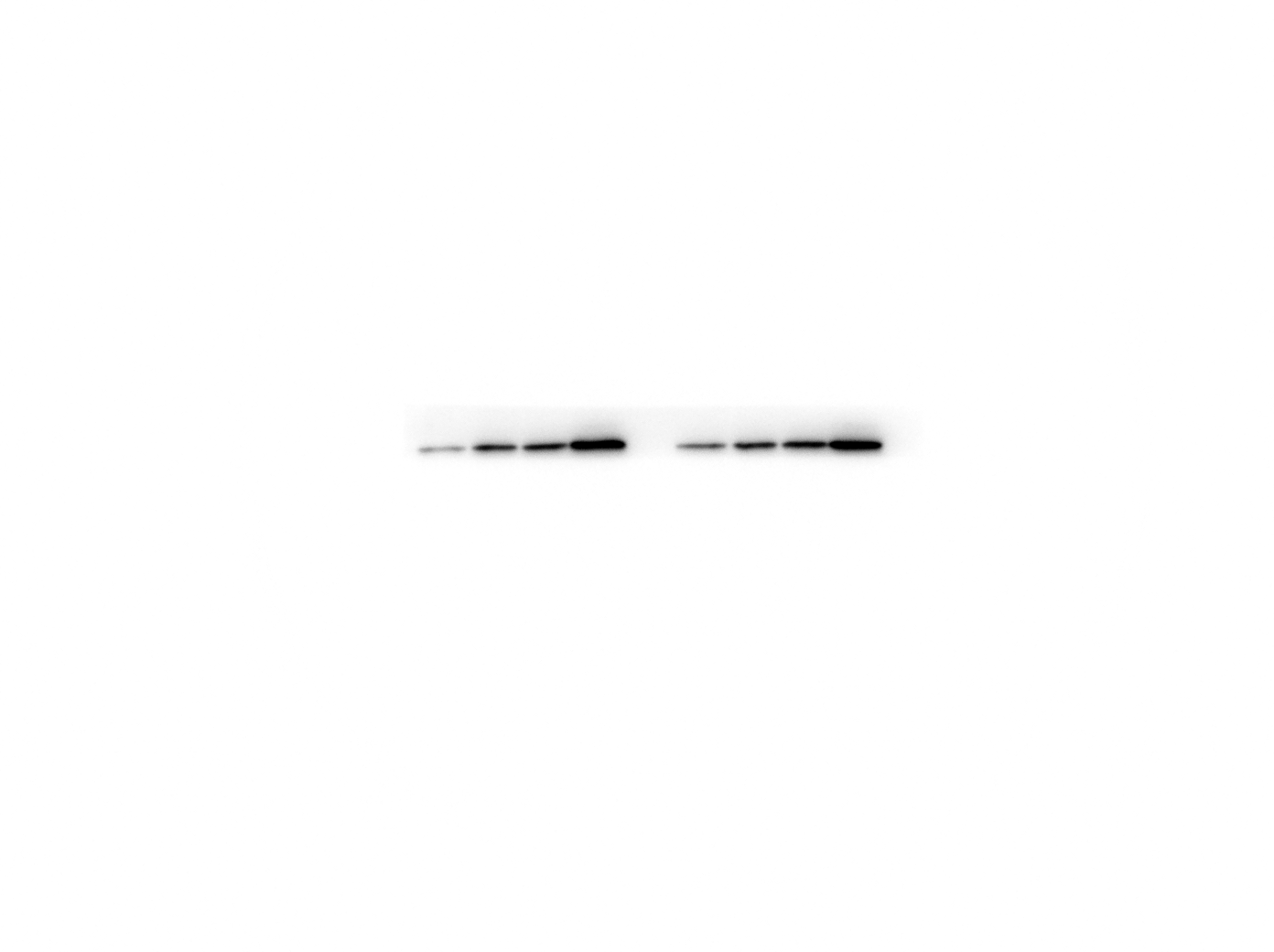

Supplement: Supplemental Information 1 [file peerj-11-14827-s001.zip › Western blot-original gels/Figure 4B-p62.tif]

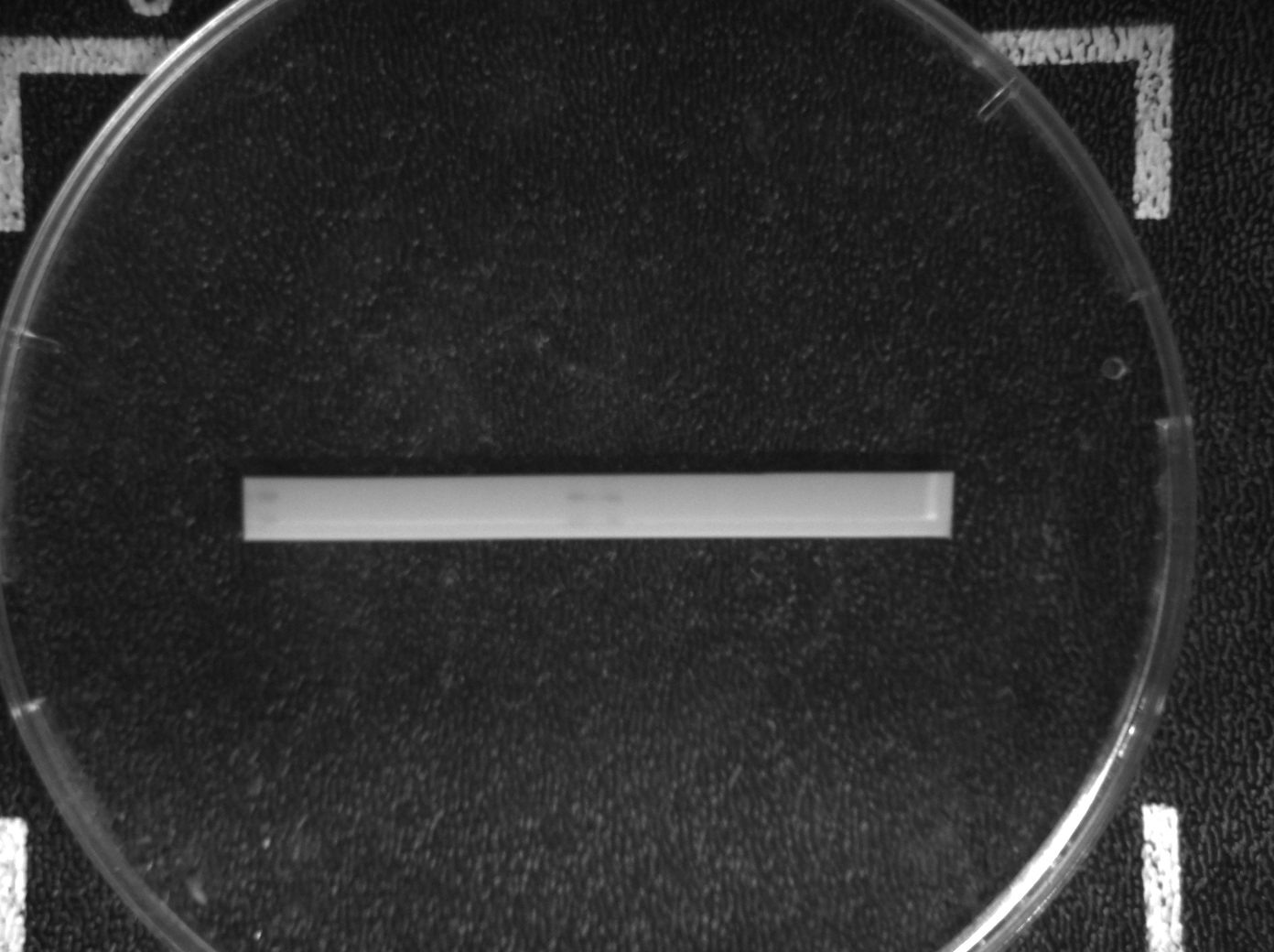

Supplement: Supplemental Information 1 [file peerj-11-14827-s001.zip › Western blot-original gels/Figure 4B-tubulin-white light.tif]

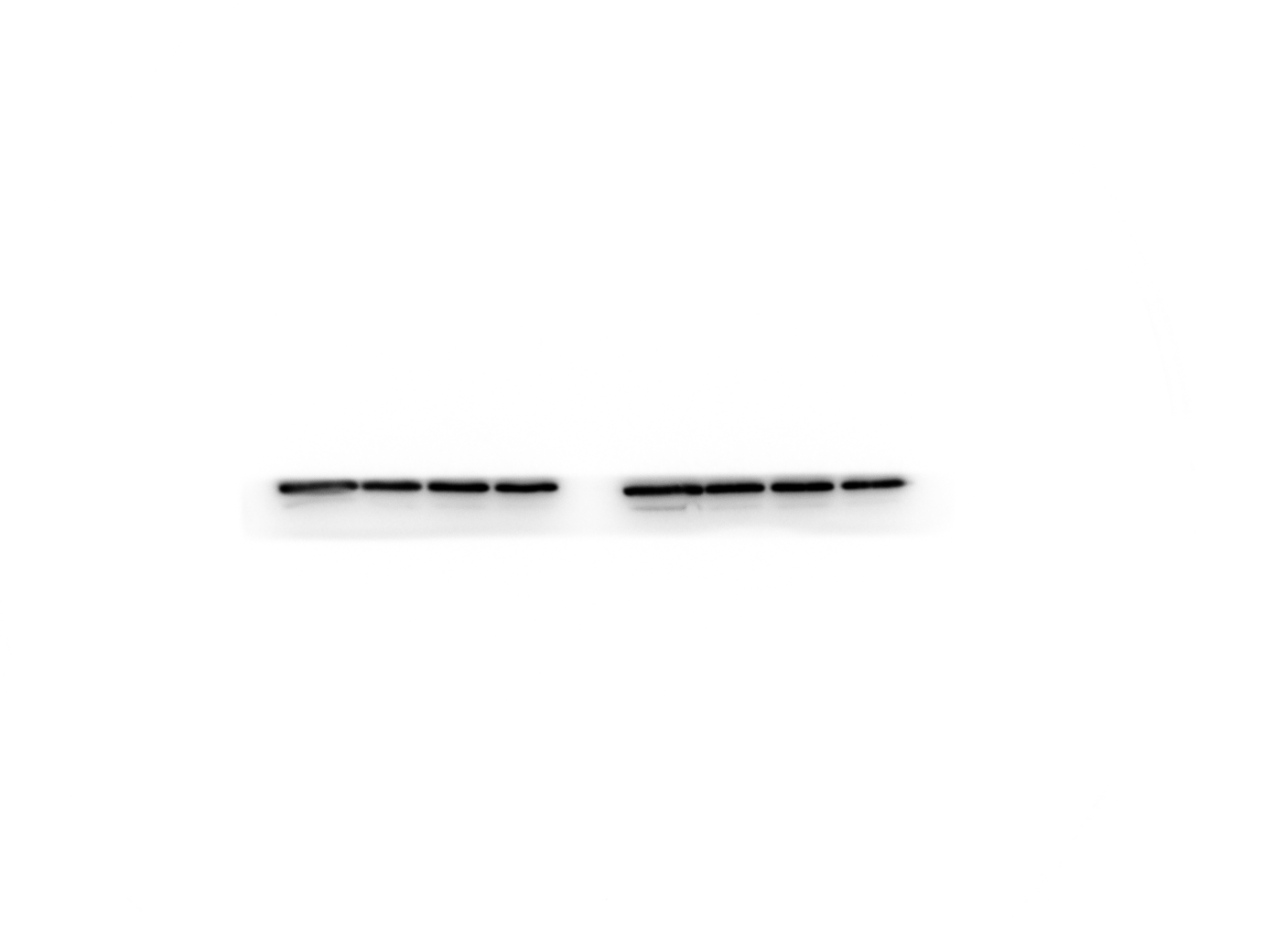

Supplement: Supplemental Information 1 [file peerj-11-14827-s001.zip › Western blot-original gels/Figure 4B-tubulin.tif]

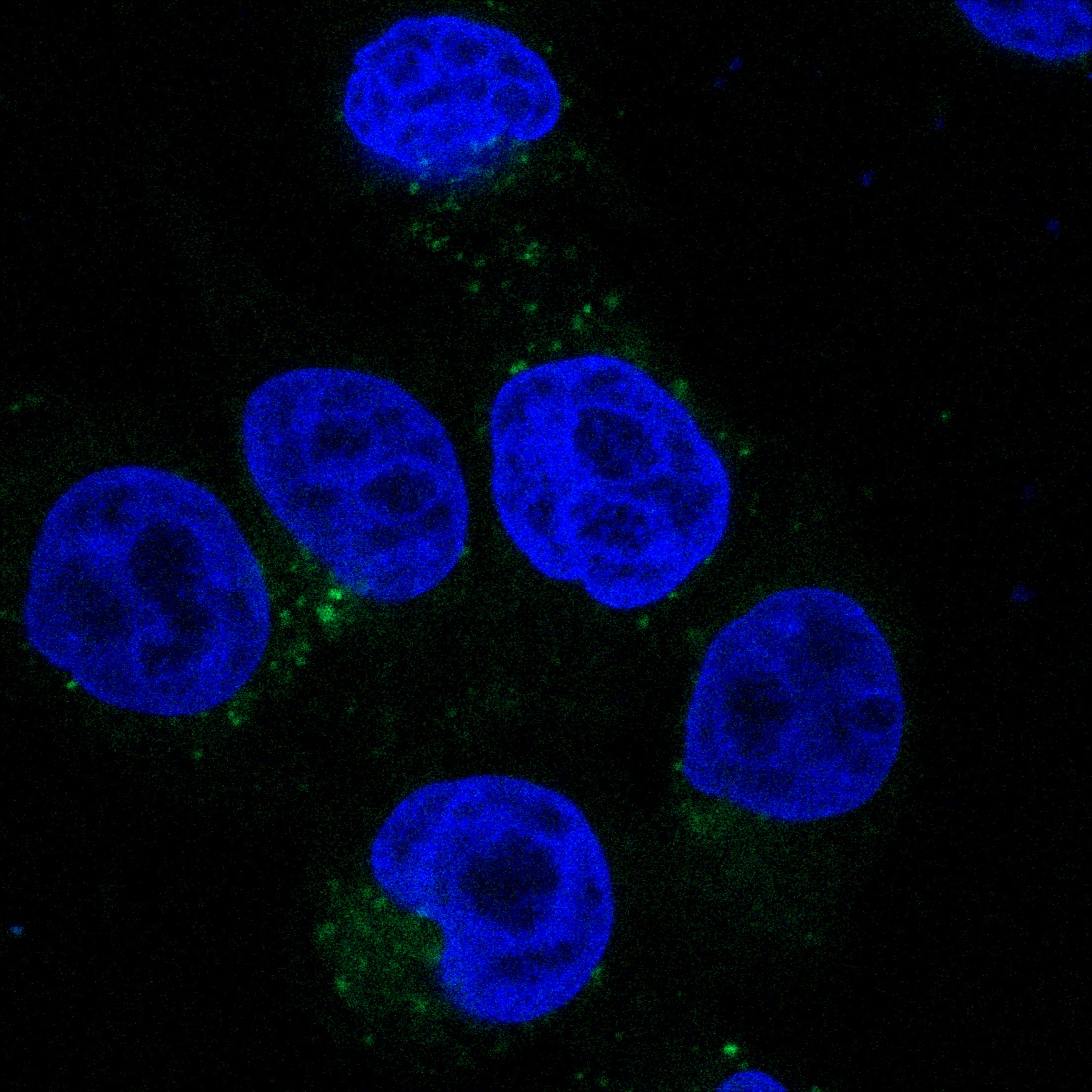

Supplement: Supplemental Information 2 [file peerj-11-14827-s002.zip › Immunofluorescence/Figure 3D-siCon.jpg]

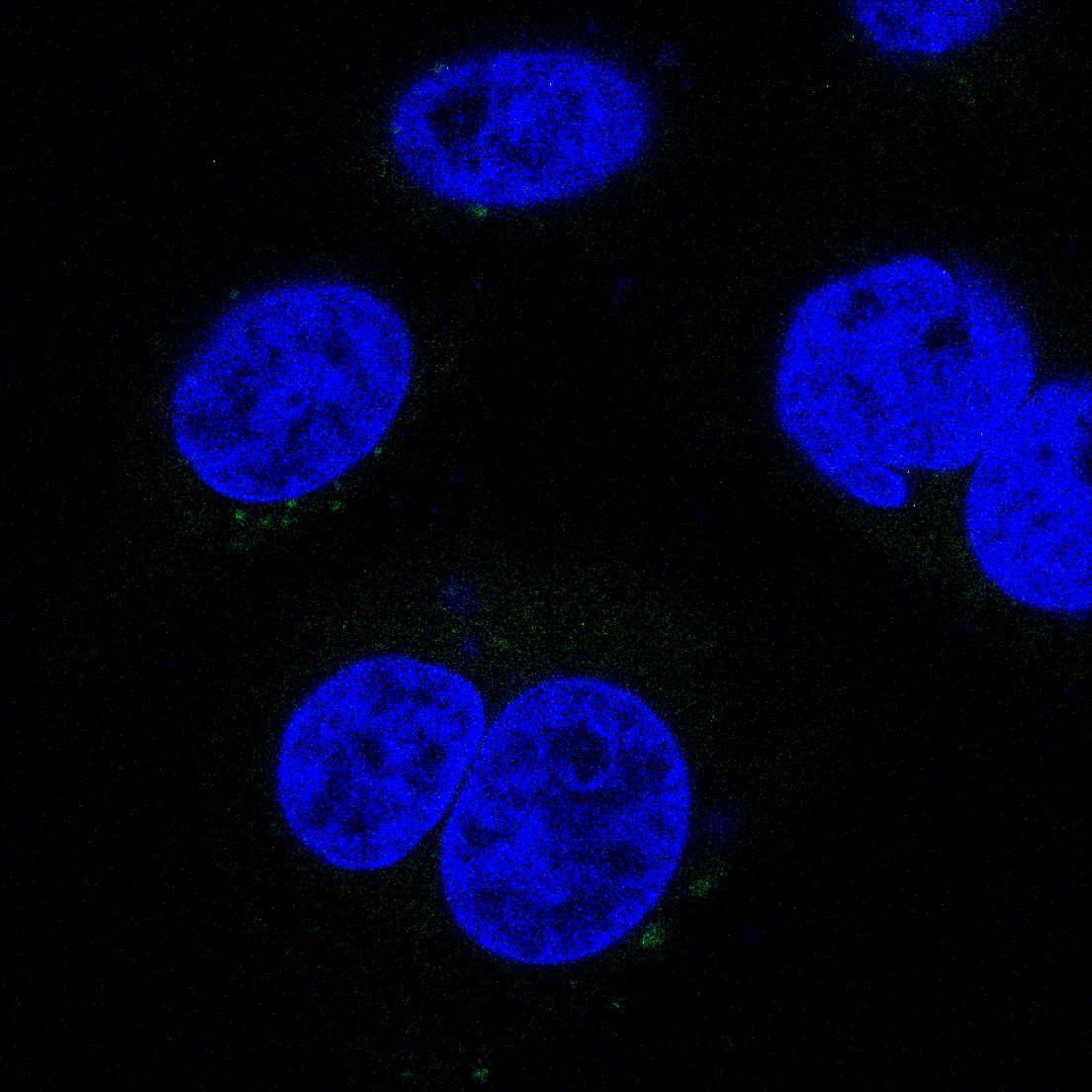

Supplement: Supplemental Information 2 [file peerj-11-14827-s002.zip › Immunofluorescence/Figure 3D-siFGF19.jpg]

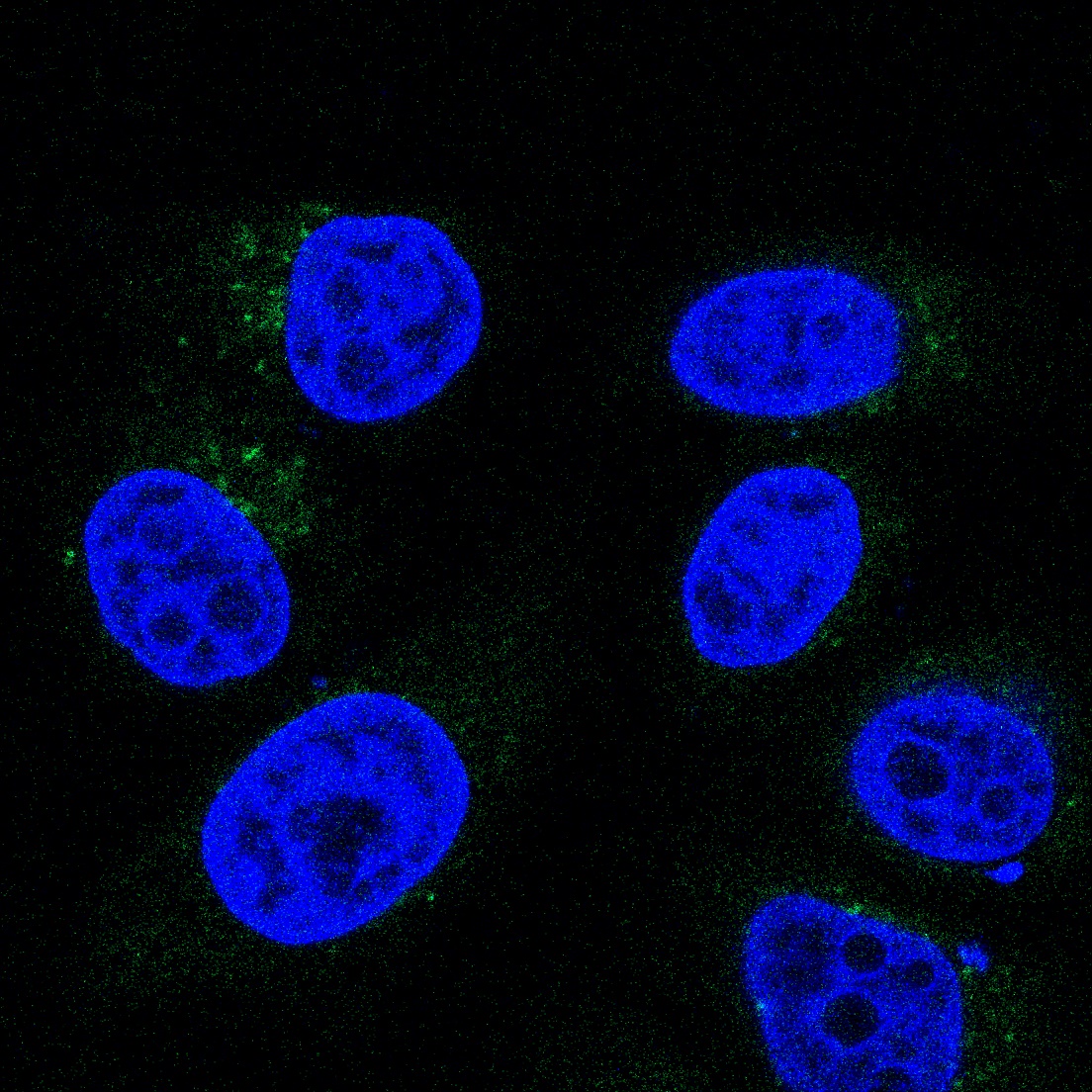

Supplement: Supplemental Information 2 [file peerj-11-14827-s002.zip › Immunofluorescence/Figure 3F-siCon.jpg]

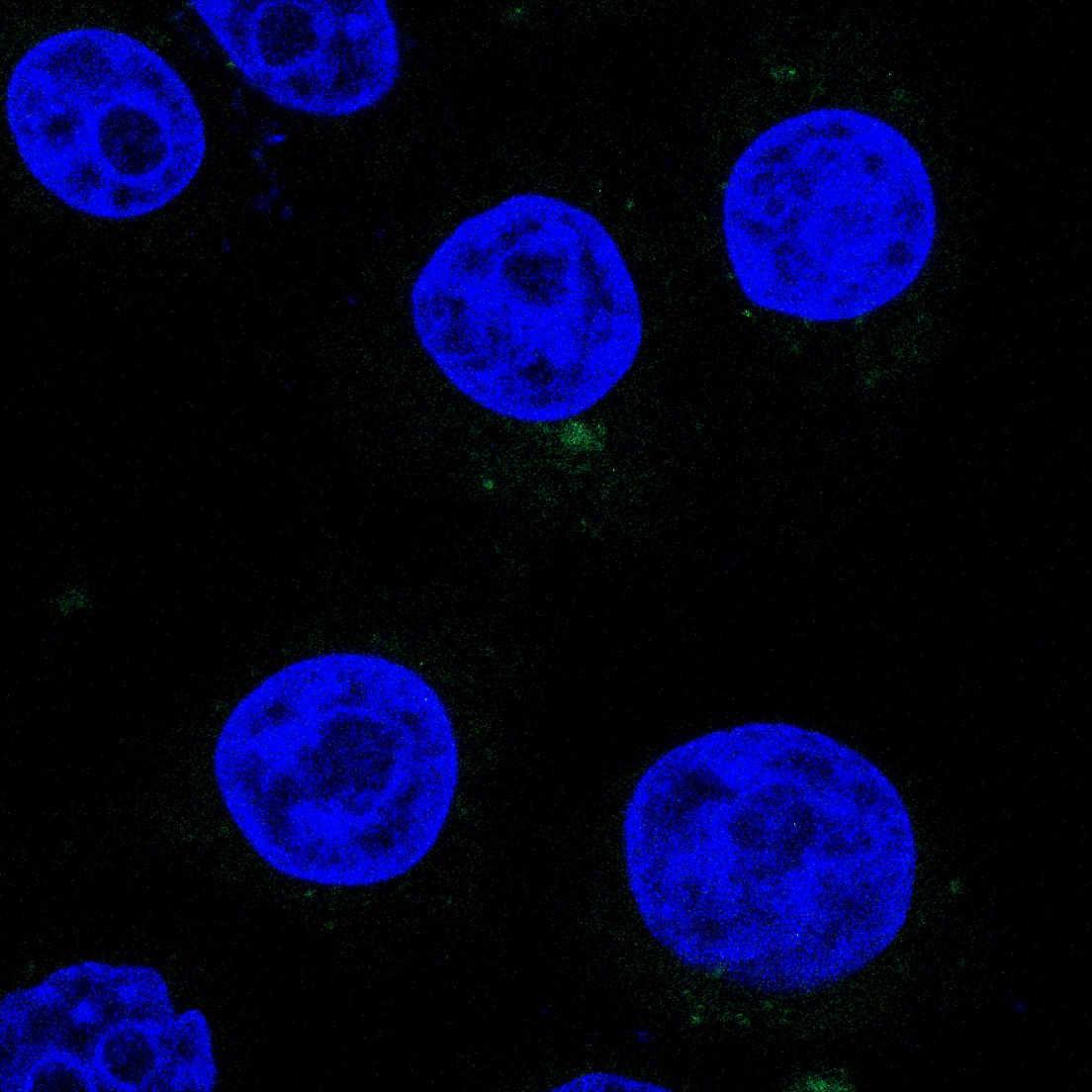

Supplement: Supplemental Information 2 [file peerj-11-14827-s002.zip › Immunofluorescence/Figure 3F-siFGF19.jpg]

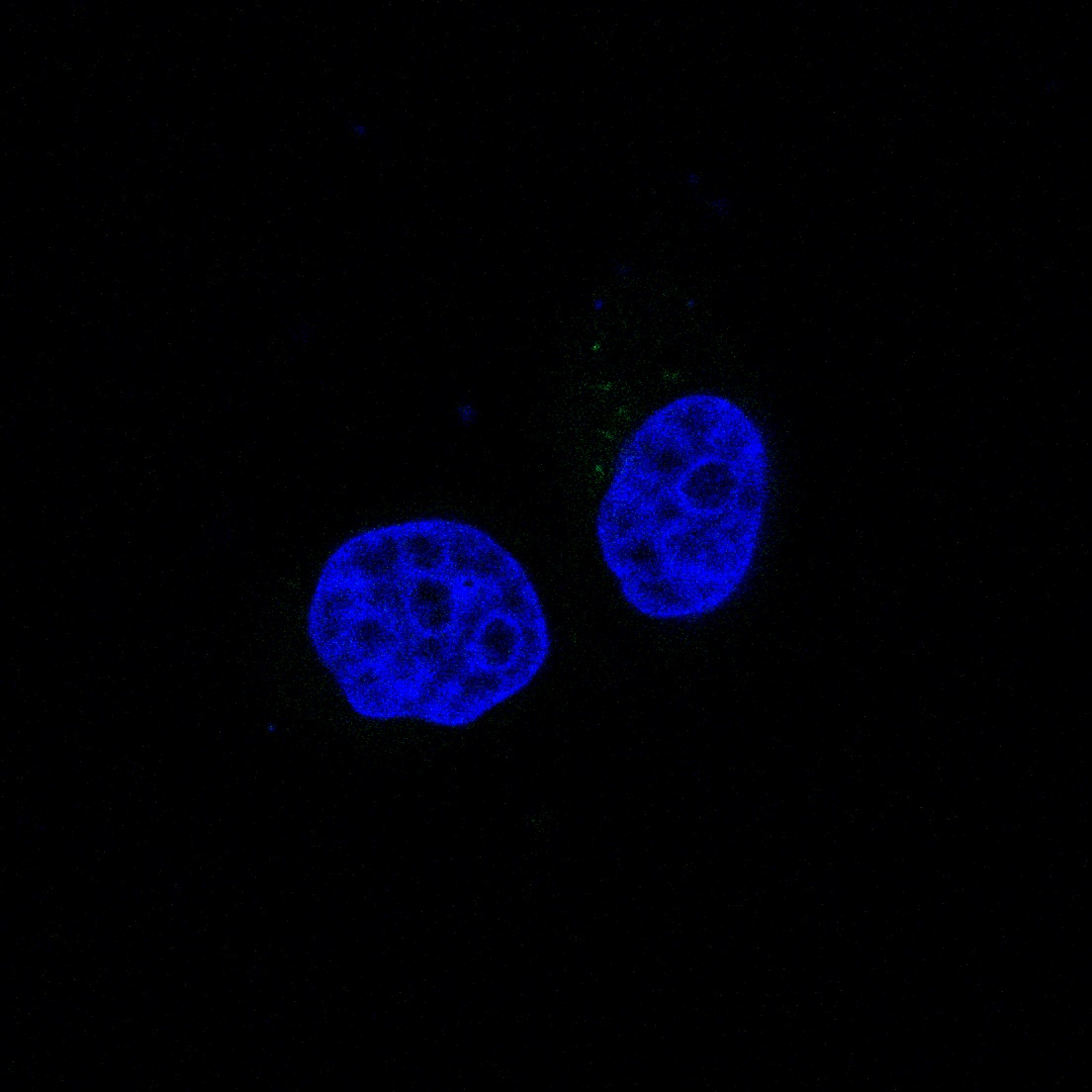

Supplement: Supplemental Information 2 [file peerj-11-14827-s002.zip › Immunofluorescence/Figure 4C(siFGF19+&SB202190+).jpg]

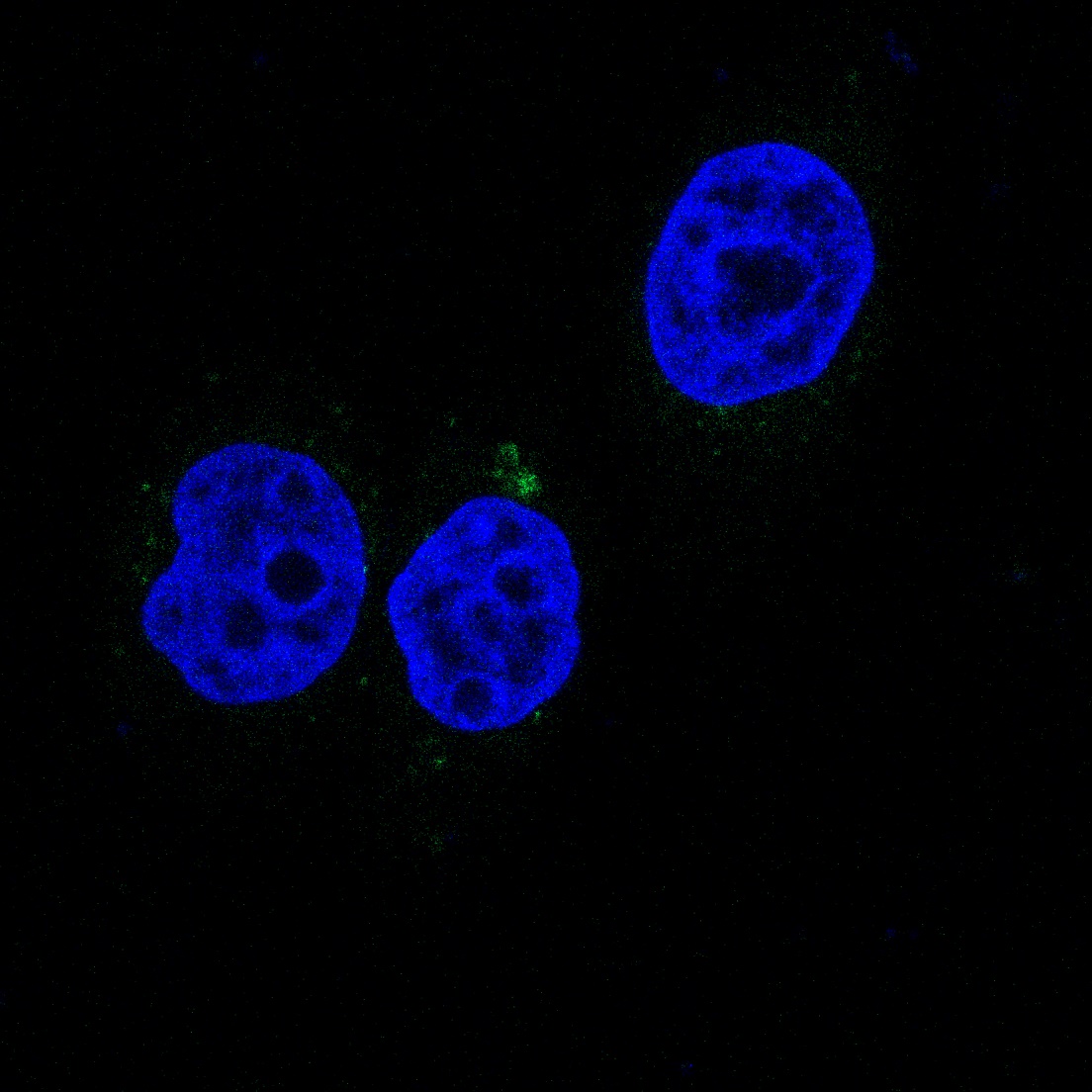

Supplement: Supplemental Information 2 [file peerj-11-14827-s002.zip › Immunofluorescence/Figure 4C(siFGF19+&SB202190-).jpg]

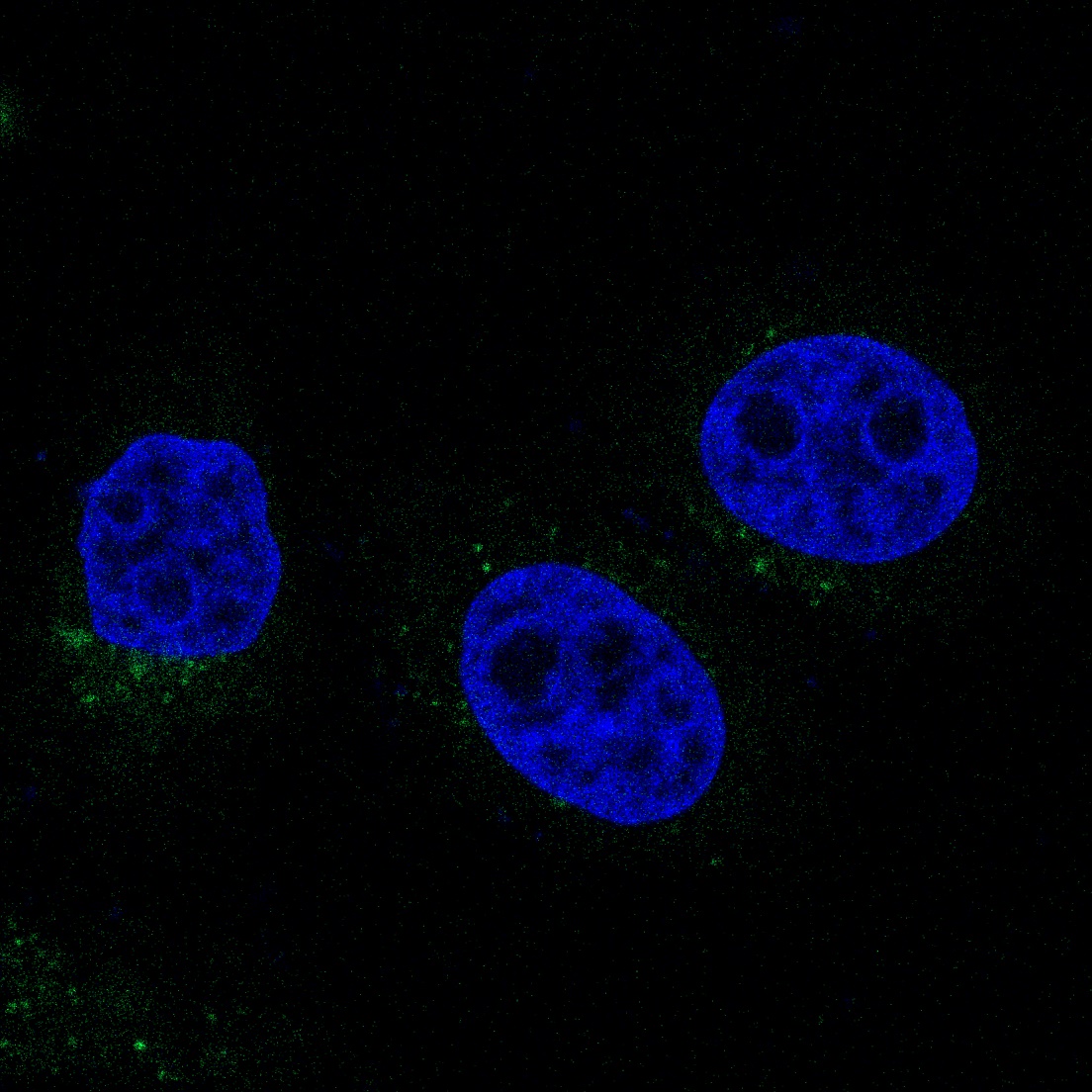

Supplement: Supplemental Information 2 [file peerj-11-14827-s002.zip › Immunofluorescence/Figure 4C(siFGF19-&SB202190+).jpg]

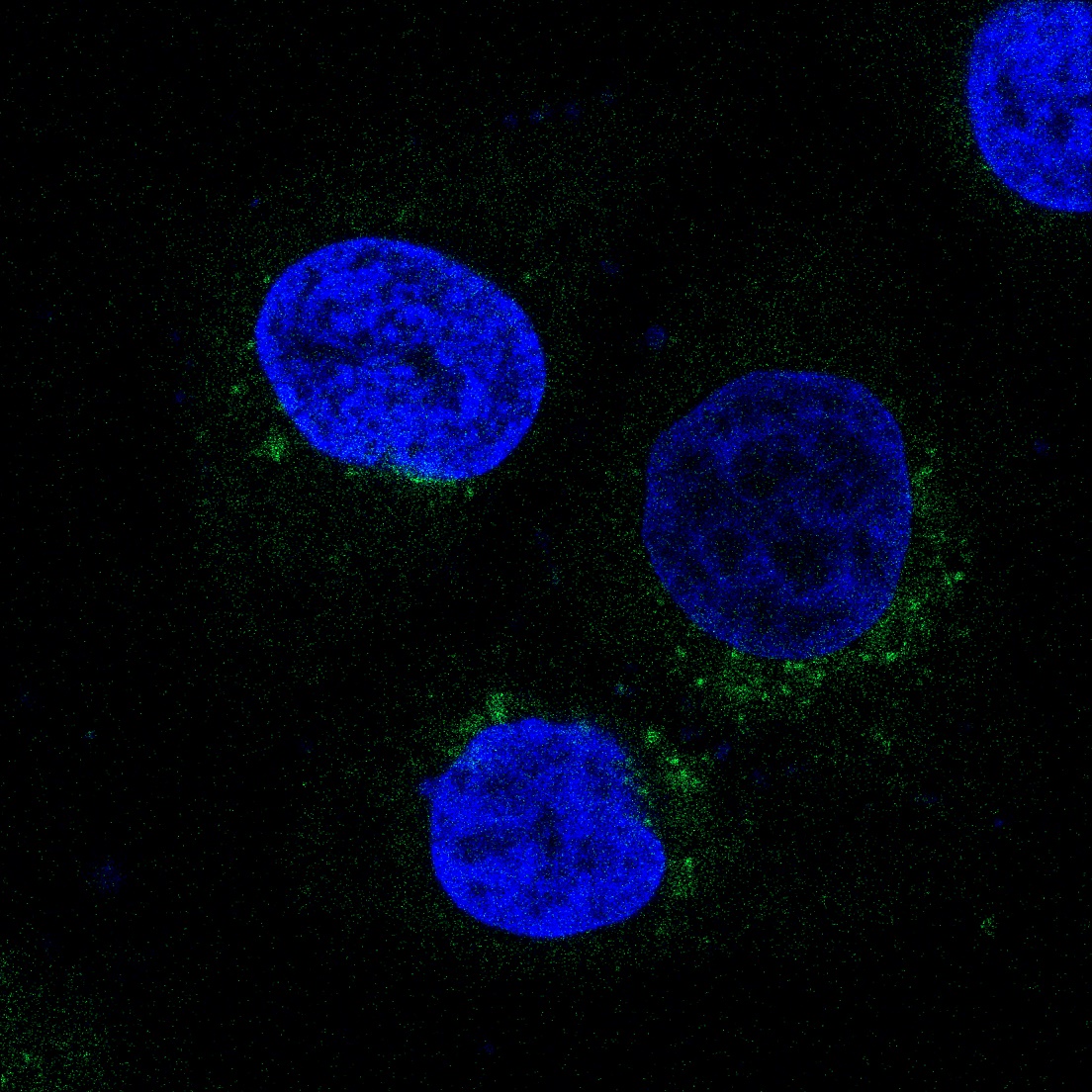

Supplement: Supplemental Information 2 [file peerj-11-14827-s002.zip › Immunofluorescence/Figure 4C(siFGF19-&SB202190-).jpg]

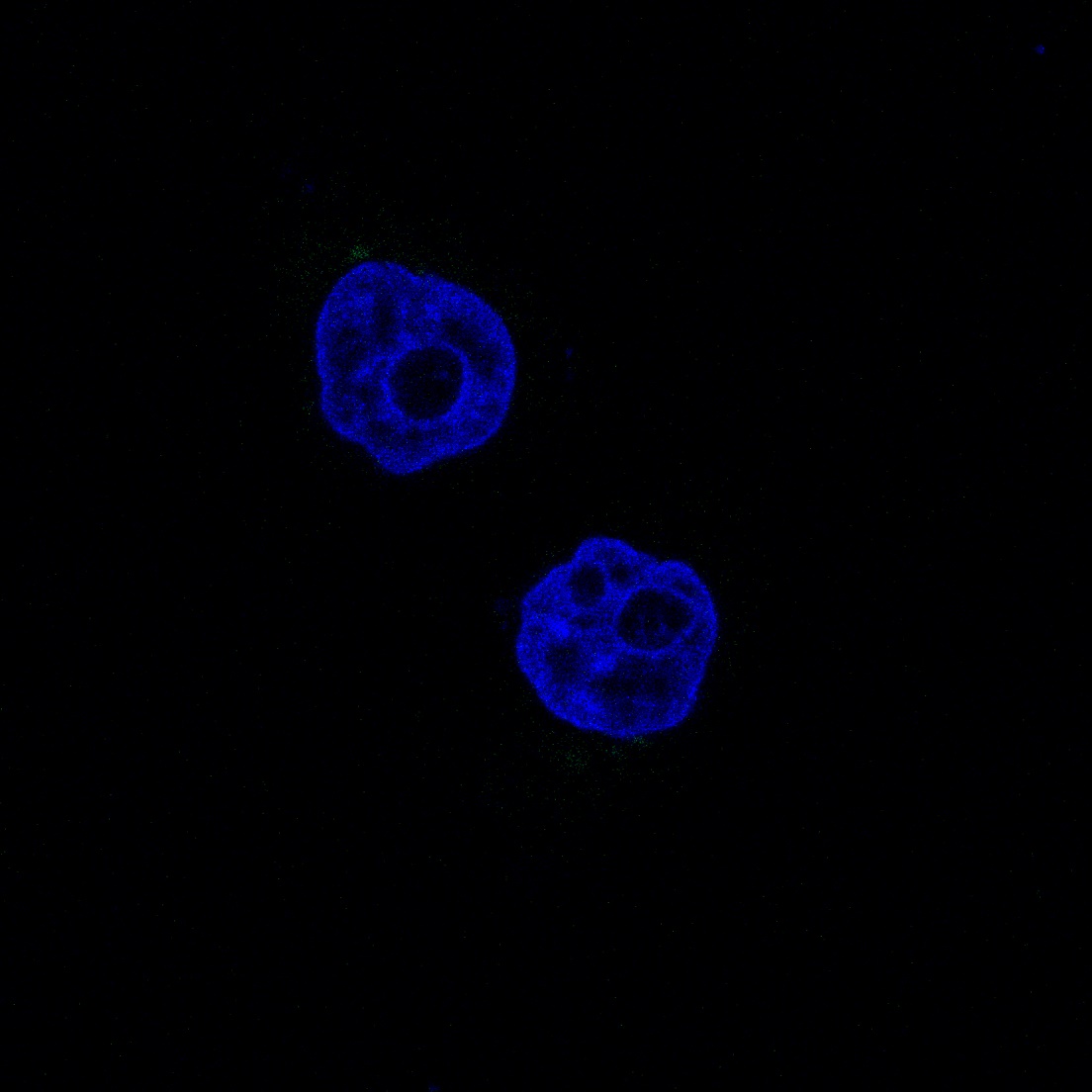

Supplement: Supplemental Information 2 [file peerj-11-14827-s002.zip › Immunofluorescence/Figure 4E(siFGF19+&SB202190+).jpg]

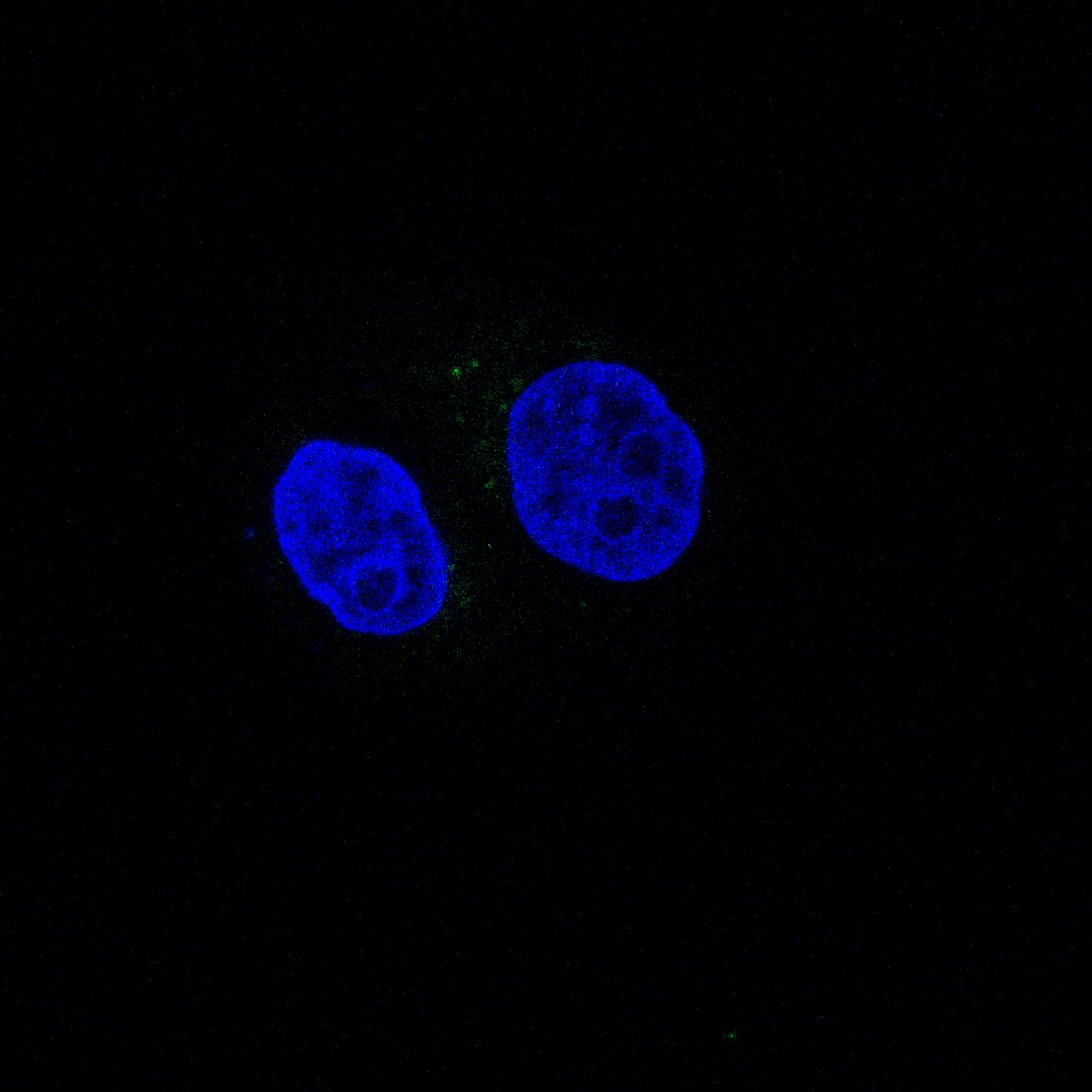

Supplement: Supplemental Information 2 [file peerj-11-14827-s002.zip › Immunofluorescence/Figure 4E(siFGF19+&SB202190-).jpg]

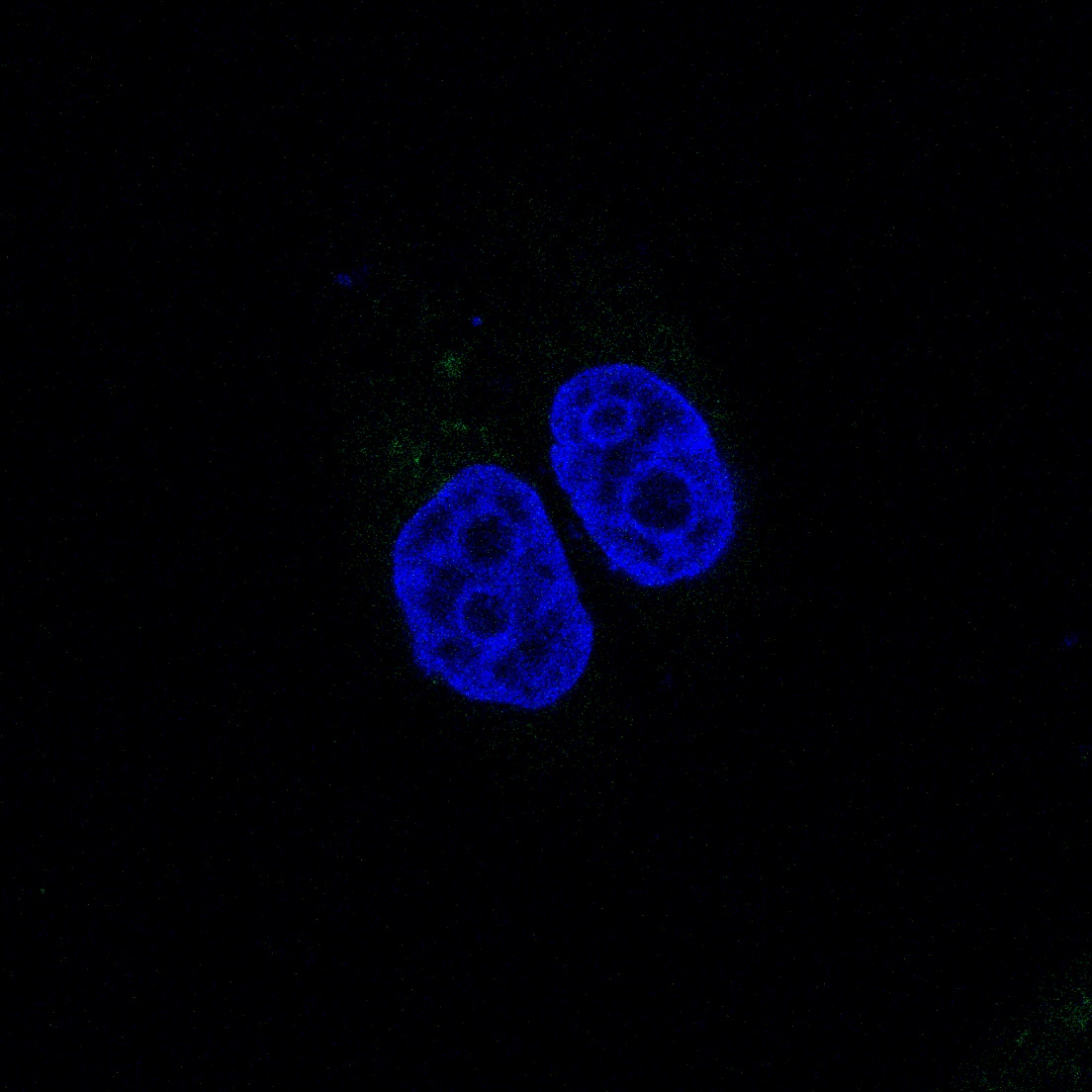

Supplement: Supplemental Information 2 [file peerj-11-14827-s002.zip › Immunofluorescence/Figure 4E(siFGF19-&SB202190+).jpg]

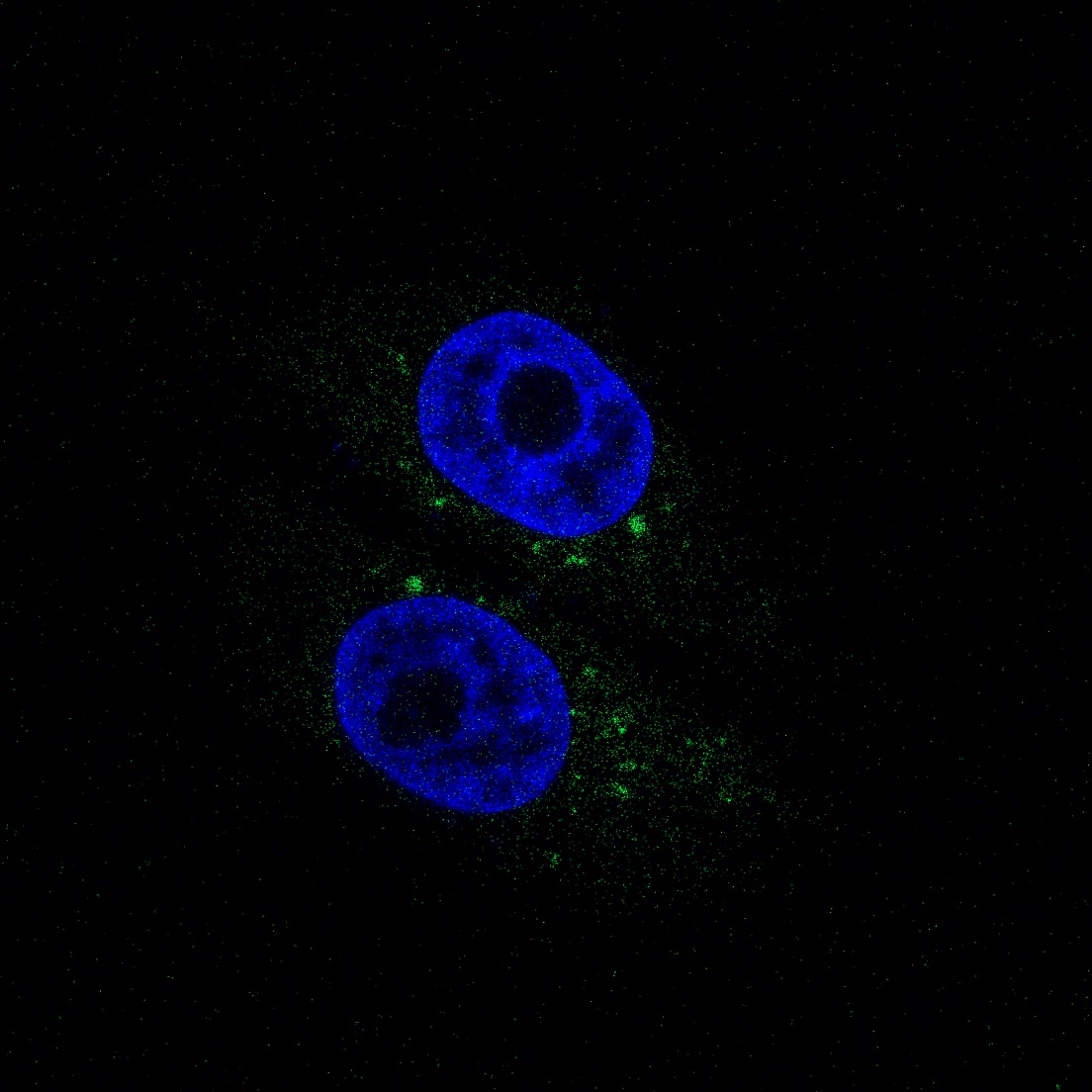

Supplement: Supplemental Information 2 [file peerj-11-14827-s002.zip › Immunofluorescence/Figure 4E(siFGF19-&SB202190-).jpg]

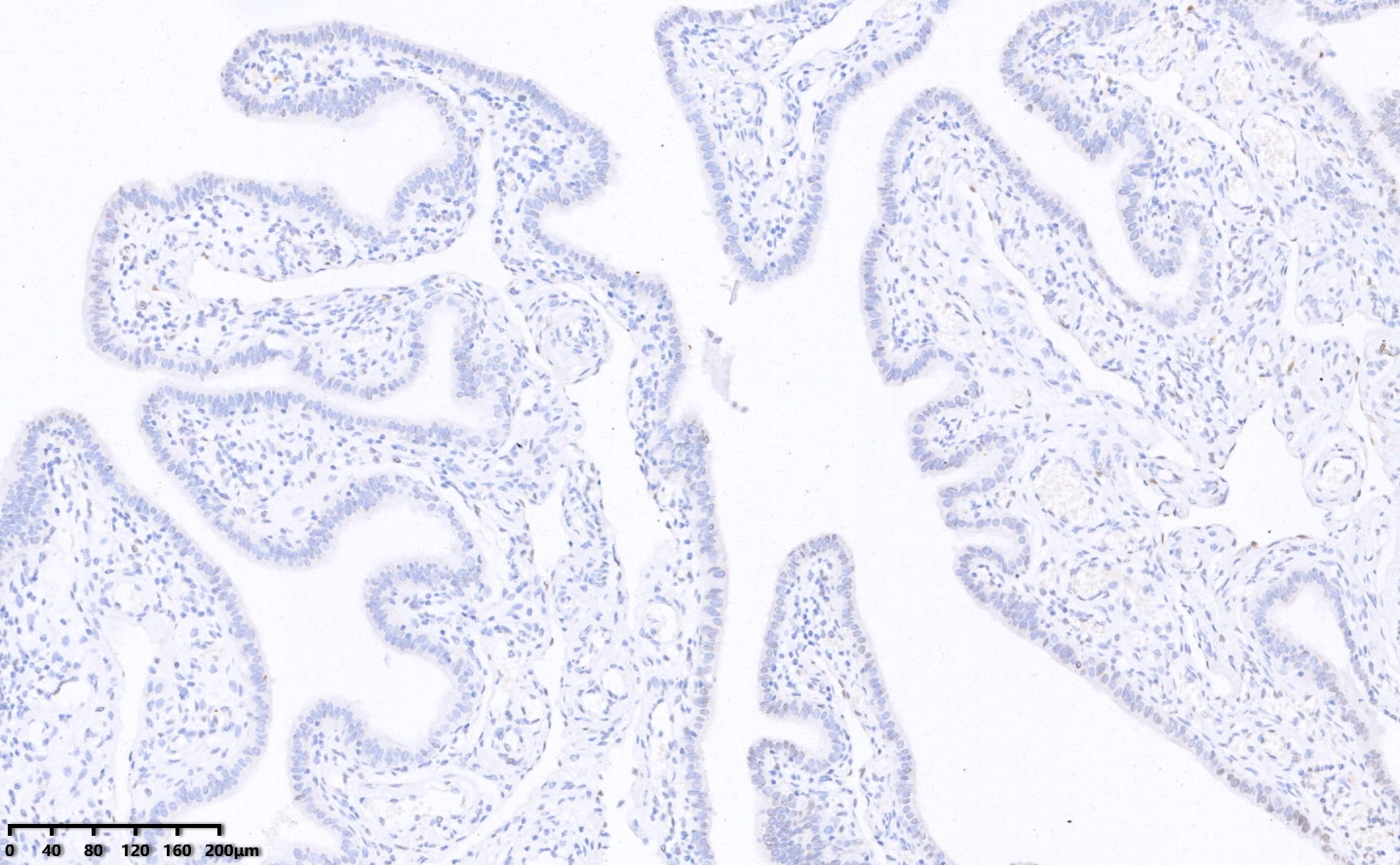

Supplement: Supplemental Information 3 [file peerj-11-14827-s003.zip › Immunohistochemical/Figure 2C-Normal fallopian tube.jpg]

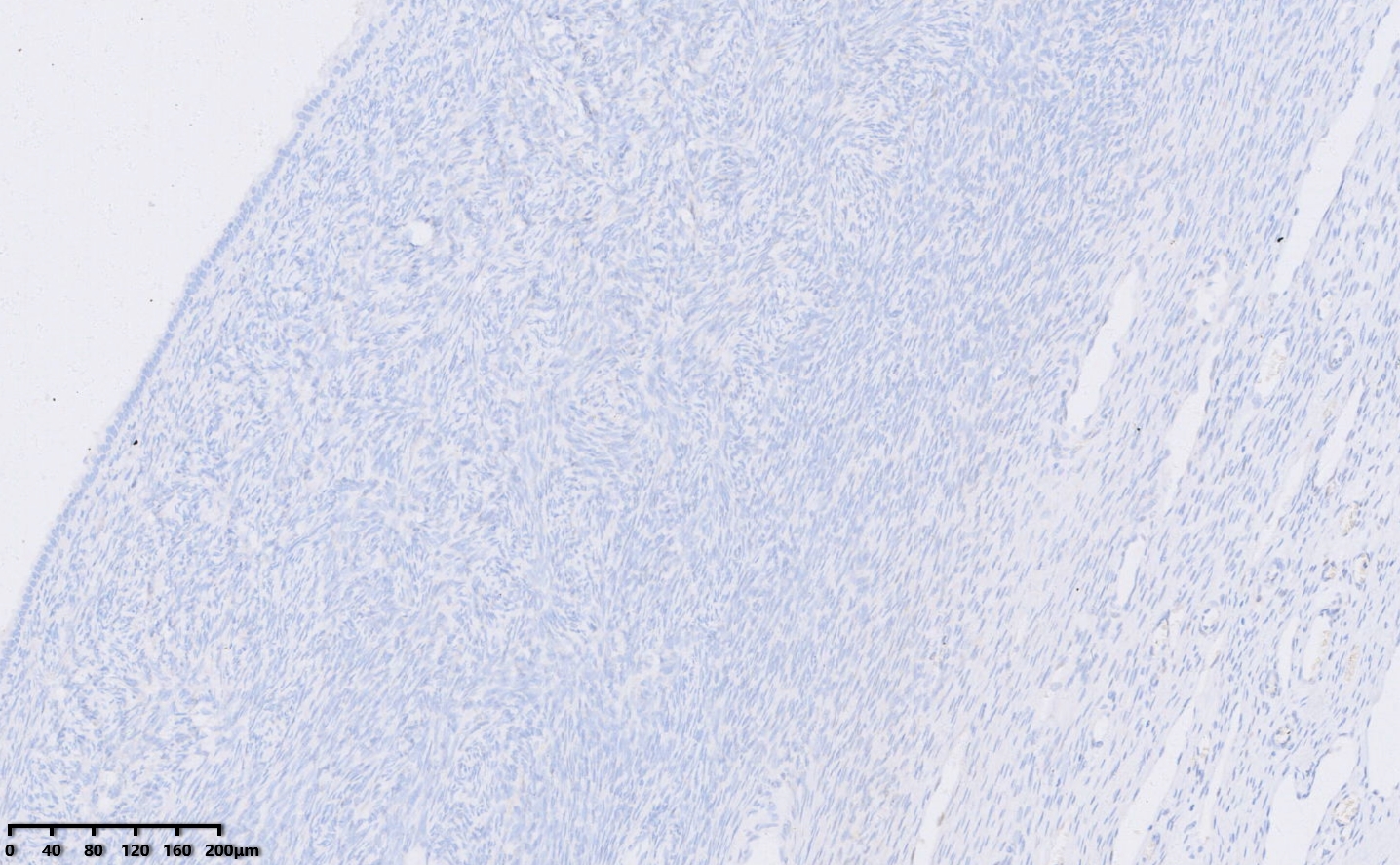

Supplement: Supplemental Information 3 [file peerj-11-14827-s003.zip › Immunohistochemical/Figure 2C-Normal ovarian.jpg]

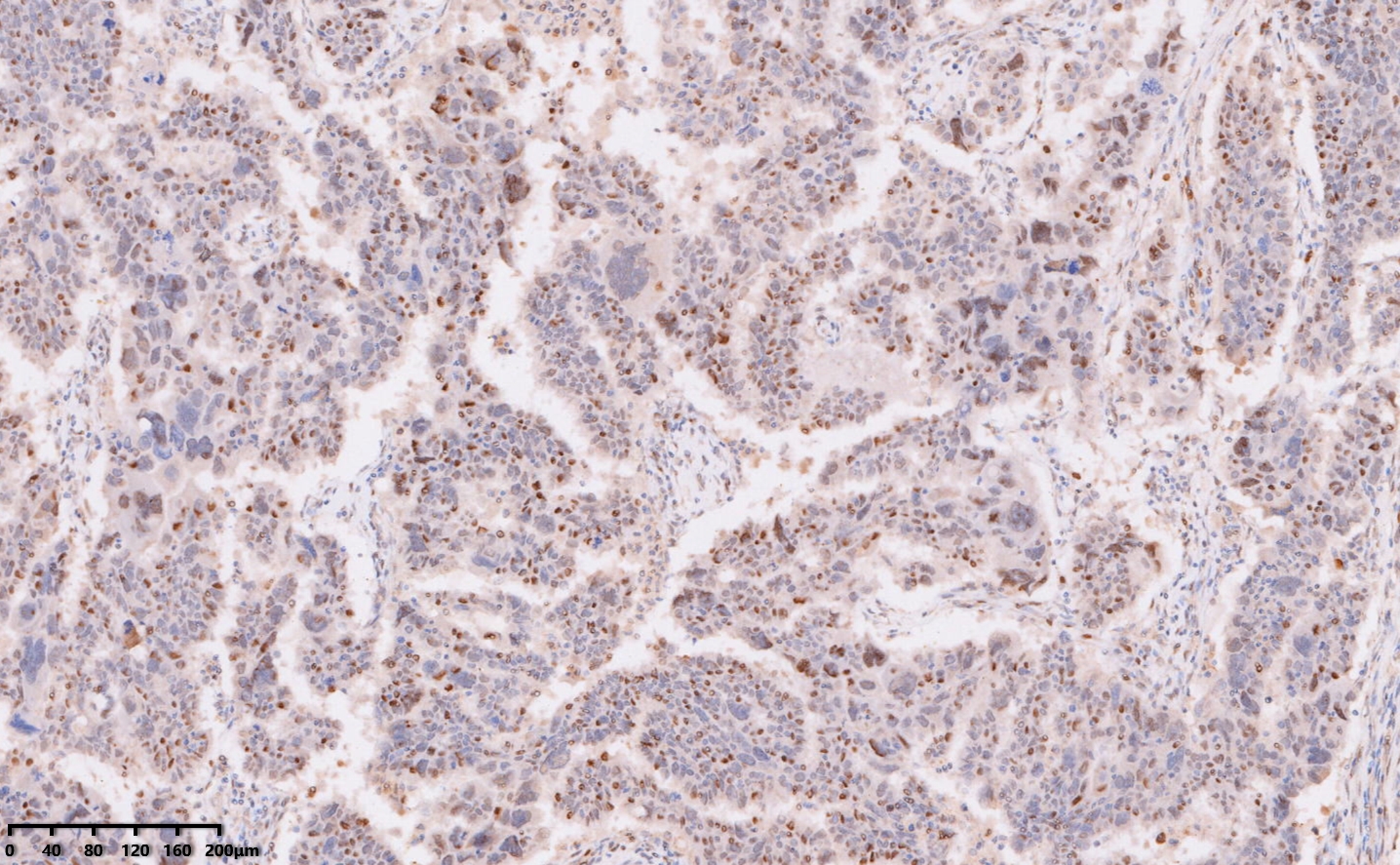

Supplement: Supplemental Information 3 [file peerj-11-14827-s003.zip › Immunohistochemical/Figure 2C-Tumor.jpg]

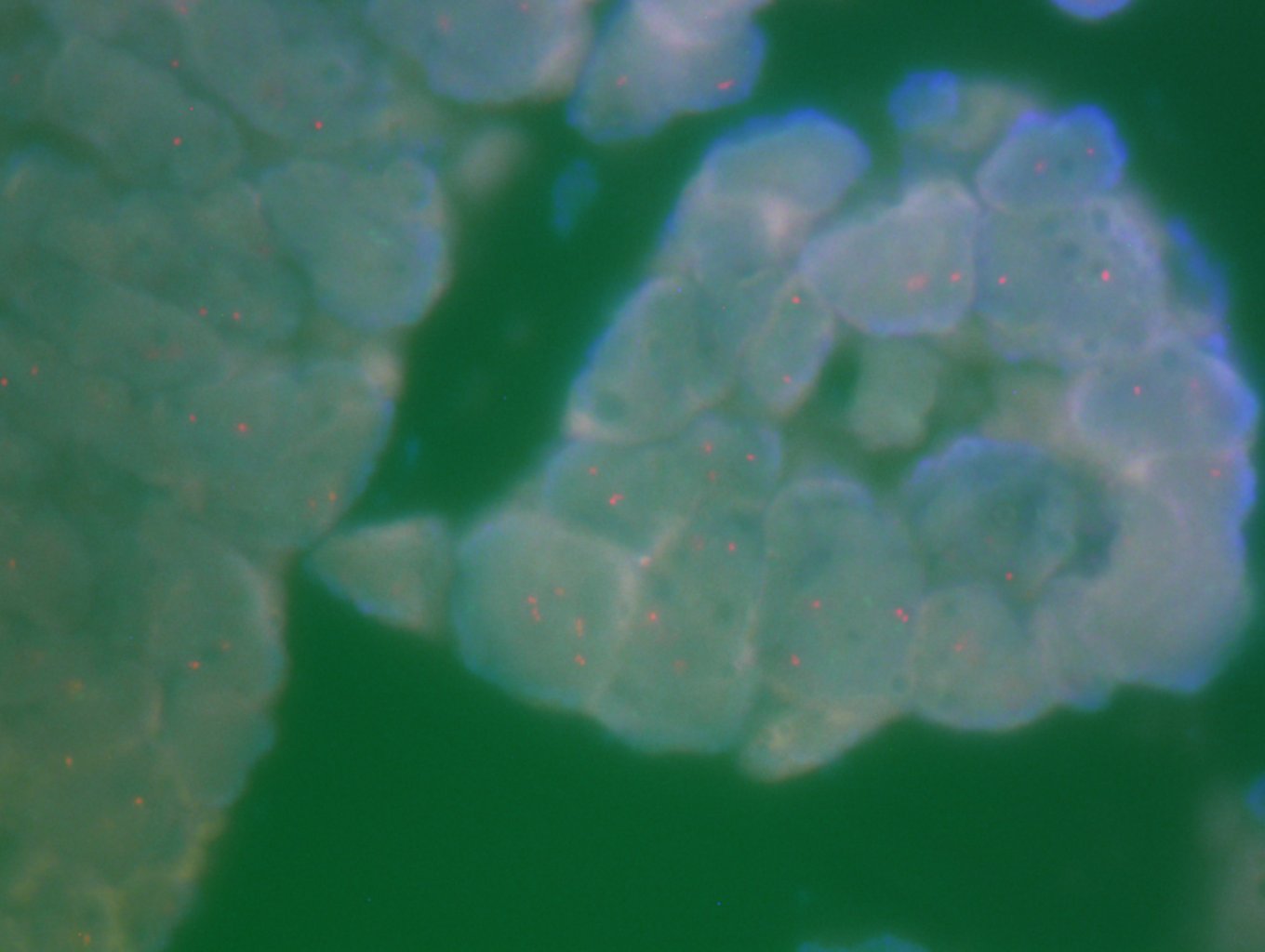

Supplement: Supplemental Information 6 [file peerj-11-14827-s006.zip › FISH/Figure1E-case1.jpg]

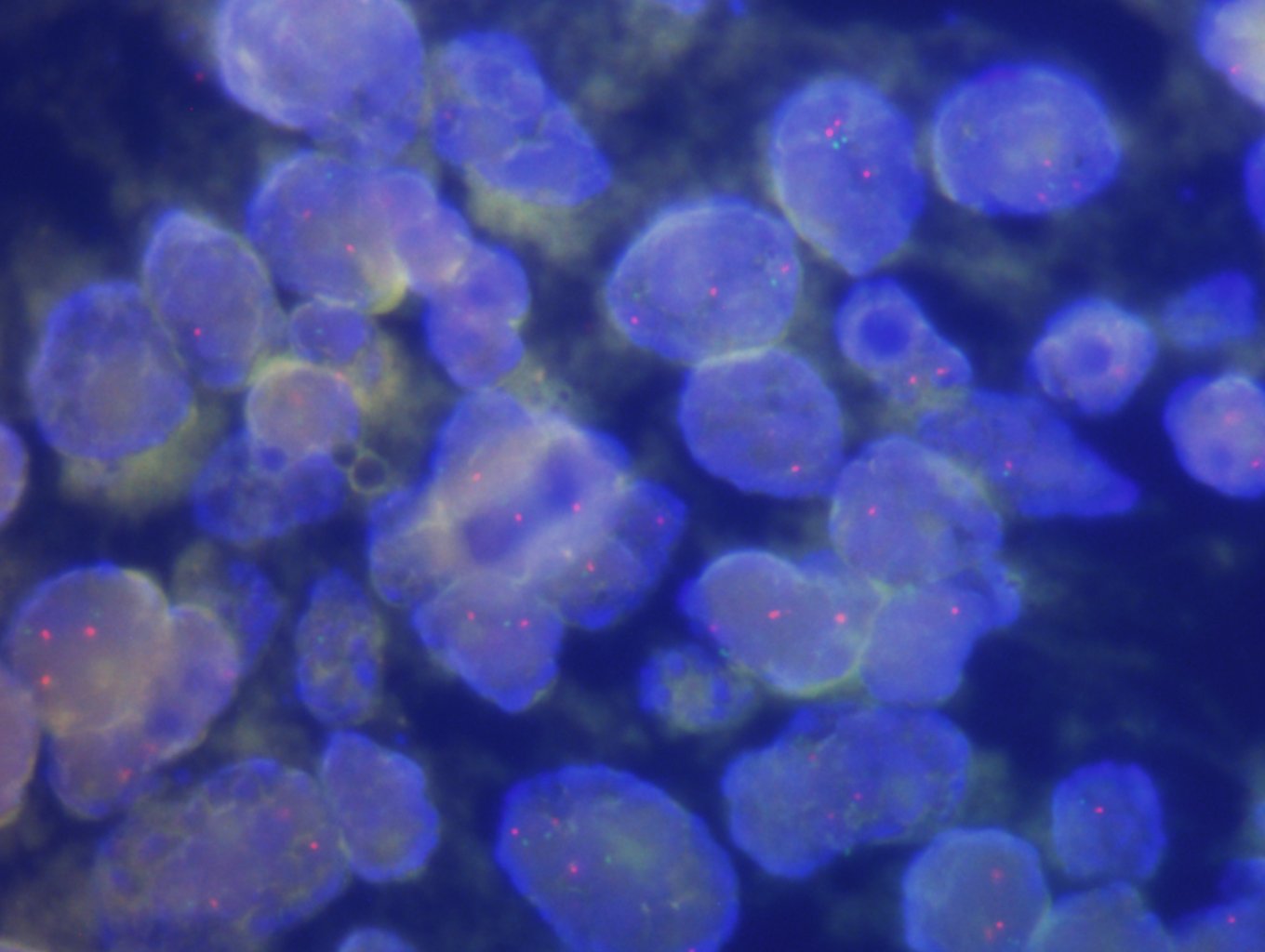

Supplement: Supplemental Information 6 [file peerj-11-14827-s006.zip › FISH/Figure1E-case2.jpg]

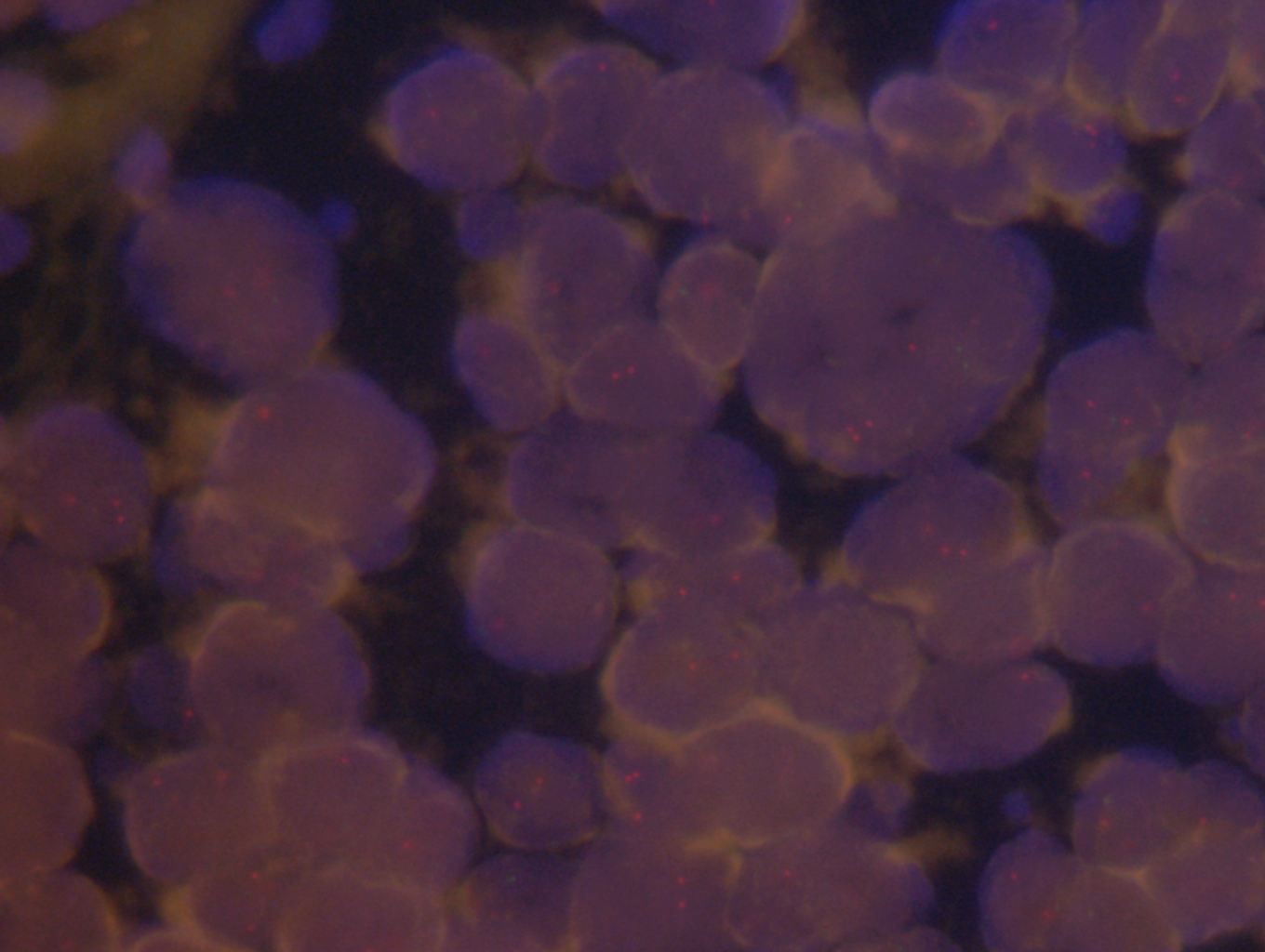

Supplement: Supplemental Information 6 [file peerj-11-14827-s006.zip › FISH/Figure1E-case3.tif]

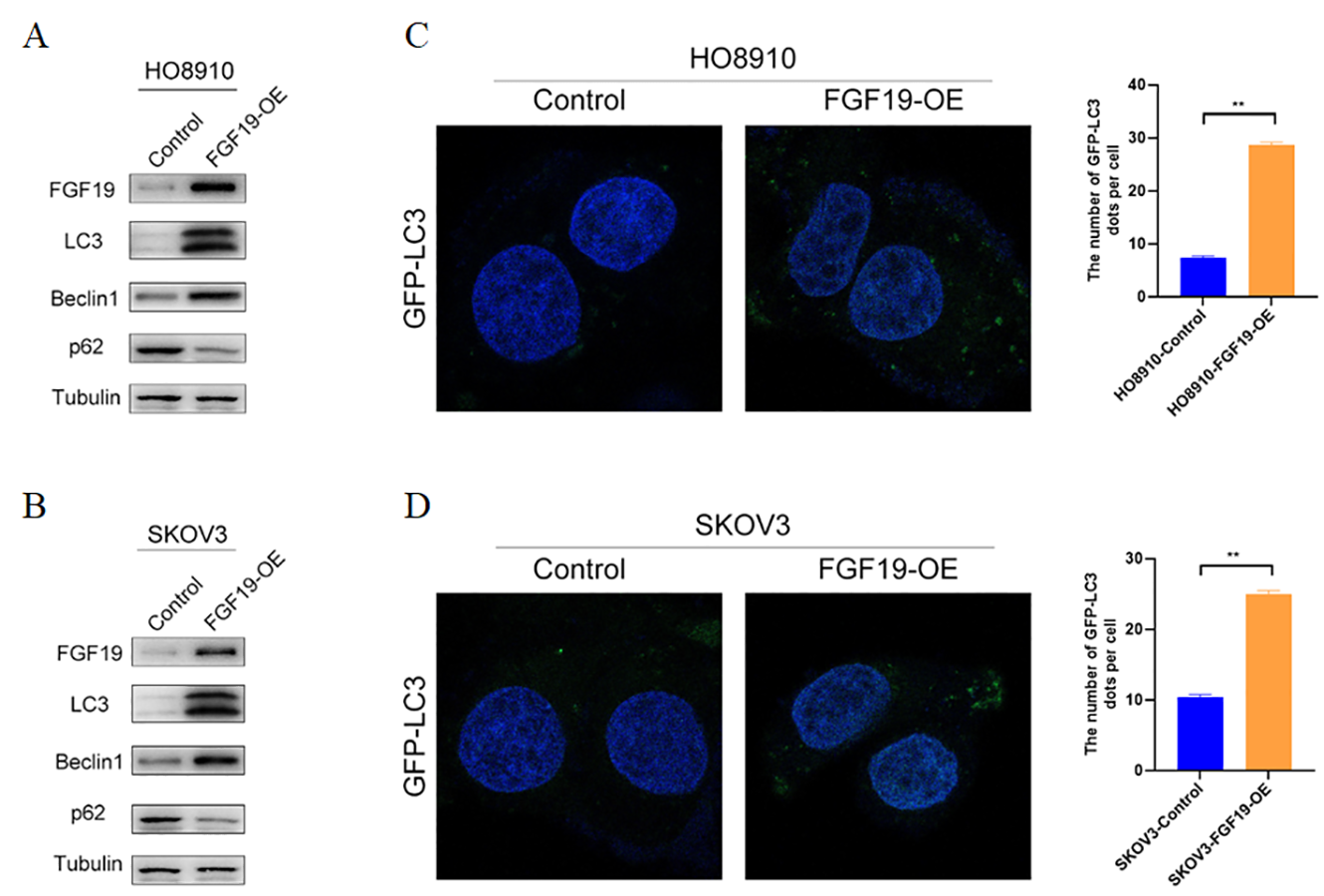

Supplement: Supplemental Information 15 — (A-B) Western blot analysis of LC3, Beclin 1, and p62 protein levels after FGF19 overexpression in HO8910 and SKOV3 cells. (C-D) Representative images of GFP-LC3 after F FGF19 overexpression in HO8910 and SKOV3 cells. *p < 0.05, **p < 0.01, ***p < 0.001 and ****p < 0.0001, ns, not significant. [file peerj-11-14827-s015.png]
