# Supplementary material for: FGF19 promotes cell autophagy and cisplatin chemoresistance by activating MAPK signaling in ovarian cancer
Source: PeerJ. 2023 Feb 2;11:e14827. doi: 10.7717/peerj.14827 (PMC9899438; doi:10.7717/peerj.14827)
Supplement: Supplemental Information 14 [file peerj-11-14827-s014.docx]

The bioinformatics data in Fig 1C,1D, 2A,2B, 2D and 2E, are automatically generated by the database. Please see the database links. Thank you.

Fig 1C

[cBioPortal for Cancer Genomics: FGF19 in MSK-IMPACT Clinical Sequencing Cohort (MSKCC, Nat Med 2017) and 9 other studies](https://www.cbioportal.org/results/cancerTypesSummary?cancer_study_list=msk_ch_2020%2Cpan_origimed_2020%2Cmsk_met_2021%2Cmsk_impact_2017%2Cmixed_allen_2018%2Cmetastatic_solid_tumors_mich_2017%2Cpancan_pcawg_2020%2Csummit_2018%2Ctmb_mskcc_2018%2Cntrk_msk_2019&Z_SCORE_THRESHOLD=2.0&RPPA_SCORE_THRESHOLD=2.0&profileFilter=mutations%2Cfusion%2Ccna%2Cgistic&case_set_id=all&gene_list=FGF19&geneset_list=%20&tab_index=tab_visualize&Action=Submit&plots_horz_selection=%7B%22dataType%22%3A%22clinical_attribute%22%2C%22selectedDataSourceOption%22%3A%22CANCER_TYPE%22%7D&plots_vert_selection=%7B%22selectedGeneOption%22%3A9965%2C%22dataType%22%3A%22clinical_attribute%22%2C%22selectedDataSourceOption%22%3A%22MUTATION_COUNT%22%7D&plots_coloring_selection=%7B%7D)

Fig 1D

[cBioPortal for Cancer Genomics: FGF19 in Ovarian Serous Cystadenocarcinoma (TCGA, Firehose Legacy) and 4 other studies](https://www.cbioportal.org/results/cancerTypesSummary?cancer_study_list=hgsoc_msk_2021%2Cov_tcga%2Cov_tcga_pub%2Cov_tcga_pan_can_atlas_2018%2Cscco_mskcc&Z_SCORE_THRESHOLD=2.0&RPPA_SCORE_THRESHOLD=2.0&profileFilter=mutations%2Cfusion%2Ccna%2Cgistic&case_set_id=all&gene_list=FGF19&geneset_list=%20&tab_index=tab_visualize&Action=Submit)

Fig 2A

<https://tnmplot.com/analysis/>

Fig 2B

<https://www.xiantao.love/products/apply/b18ef455-7ec2-4c63-9312-6473cbc9fe76/operation/11f7bf0d-2ed3-42a9-ab20-4cc7fb14b2e4>

Fig 2D

[Kaplan-Meier plotter [Ovarian] (kmplot.com)](http://kmplot.com/analysis/index.php?p=service)

Fig 2E

[[Kaplan-Meier plotter [Ovarian] (kmplot.com)](http://kmplot.com/analysis/index.php?p=service)](http://www.kmplot.com/analysis/index.php?p=service)
