# Supplementary material for: FGF19 promotes cell autophagy and cisplatin chemoresistance by activating MAPK signaling in ovarian cancer
Source: PeerJ. 2023 Feb 2;11:e14827. doi: 10.7717/peerj.14827 (PMC9899438; doi:10.7717/peerj.14827)
Supplement: Supplemental Information 17 [file peerj-11-14827-s017.docx]

**The codebook of numbers in SAV file**

1.Age group

1 represents the age≥50

0 represents the age<50

2. clinical stage

1 represents stage Ⅰ

2 represents stage Ⅱ

3 represents stage Ⅲ

4 represents stage Ⅵ

3.lymphatic metastasis

1 represents yes

0 represents no

4.Abdominal and distant metastases

1 represents yes

0 represents no

5.Ovarian cancer FGF19（IHC）

expression of FGF19 in ovarian cancer

0 represents “-“

1 represents ”+”

2 represents “++”

3 represents ”+++”

6.Normal ovarian FGF19（IHC）

expression of FGF19 in normal ovarian

0 represents “-“

1 represents ”+”

2 represents “++”

3 represents ”+++”

7. Normal fallopian tube FGF19(IHC)

expression of FGF19 in normal fallopian tube

0 represents “-“

1 represents ”+”

2 represents “++”

3 represents ”+++”

8. FGF19 group（cancer）

1 represents ”++” or ”+++”

0 represents “-“ or “+”
